# Six 1-halobenzoyl-4-(2-meth­oxy­phen­yl)piperazines having *Z*′ values of one, two or four; disorder, pseudosymmetry, twinning and supra­molecular assembly in one, two or three dimensions

**DOI:** 10.1107/S2056989020015649

**Published:** 2021-01-01

**Authors:** Chayanna Harish Chinthal, Channappa N. Kavitha, Hemmige S. Yathirajan, Sabine Foro, Christopher Glidewell

**Affiliations:** a Department of Studies in Chemistry, University of Mysore, Manasagangotri, Mysuru-570 006, India; b Department of Chemistry, Maharani’s Science College for Women, Mysuru-570 001, India; c Institute of Materials Science, Darmstadt University of Technology, Alarich-Weiss-Strasse 2, D-64287 Darmstadt, Germany; d School of Chemistry, University of St Andrews, St Andrews, Fife KY16 9ST, UK

**Keywords:** piperazines, synthesis, crystal structure, mol­ecular conformation, disorder, pseudosymmetry, inversion twinning, hydrogen bonding, supra­molecular assembly

## Abstract

Six 1-halobenzoyl-4-(2-meth­oxy­phen­yl)piperazines have been prepared using carbodi­imide-mediated coupling reactions between halo­benzoic acids and *N*-(2-meth­oxy­phen­yl)piperazine. The mol­ecules of 1-(4-fluoro­benzo­yl)-4-(2-meth­oxy­phen­yl)piperazine, C_18_H_19_FN_2_O_2_ (I), are linked into a chain of rings by a combination of C—H⋯O and C—H⋯π(arene) hydrogen bonds. 1-(4-Chloro­benzo­yl)-4-(2-meth­oxy­phen­yl)piperazine, C_18_H_19_ClN_2_O_2_ (II), crystallizes in the space group *Pca*2_1_ with *Z*′ = 4 and it exhibits both pseudosymmetry and inversion twinning: a combination of six C—H⋯O and two C—H⋯π(arene) hydrogen bonds generate a three-dimensional assembly. In 1-(4-bromo­benzo­yl)-4-(2-meth­oxy­phen­yl)piperazine, C_18_H_19_BrN_2_O_2_ (III), which also crystallizes in space group *Pca*2_1_ but with *Z*′ = 2, the bromo­benzoyl unit in one of the mol­ecules is disordered. Pseudosymmetry and inversion twinning are again present, and a combination of three C—H⋯O and one C—H⋯π(arene) hydrogen bonds generate a two-dimensional assembly. A single C—H⋯O hydrogen bond links the mol­ecules of 1-(4-iodo­benzo­yl)-4-(2-meth­oxy­phen­yl)piperazine, C_18_H_19_IN_2_O_2_ (IV), into simple chains but in the isomeric 3-iodo­benzoyl analogue (V), which crystallizes in space group *P*2_1_2_1_2_1_ with *Z*′ = 2, a two-dimensional assembly is generated by a combination of four C—H⋯O and two C—H⋯π(arene) hydrogen bonds; pseudosymmetry and inversion twinning are again present. A single C—H⋯O hydrogen bond links the mol­ecules of 1-(2-fluoro­benzo­yl)-4-(2-meth­oxy­phen­yl)piperazine, C_18_H_19_FN_2_O_2_ (VI), into simple chains. Comparisons are made with the structures of some related compounds.

## Abstract 2

Among six closely related 1-halobenzoyl-4-(2-meth­oxy­phen­yl)piperazines, those with *Z*′ = 1 form one-dimensional hydrogen-bonded assemblies, those with *Z*′ = 2 form two-dimensional hydrogen-bonded assemblies, and that with *Z*′ = 4 forms a three-dimensional hydrogen-bonded assembly. Pseudosymmetry and inversion twinning are apparent when *Z*′ > 1.

## Chemical context   

*N*-(2-Meth­oxy­phen­yl)piperazine (2-MeOPP) has been used as a building block in the synthesis of both 5-HT_1A_ receptor ligands (Orjales *et al.*, 1995 ▸) and dopamine D_2_ and D_3_ ligands (Hackling *et al.*, 2003 ▸), and also as a building block for the synthesis of derivatives exhibiting anti­depressant-like activity (Waszkielewicz *et al.*, 2015 ▸). We have recently reported the structures of a range of salts derived from 2-MeOPP (Harish Chinthal *et al.*, 2020*a* ▸) and here we report the syntheses and structures of six 1-haloaroyl-4-(2-meth­oxy­phen­yl)piperazines, (I)–(VI). The work reported here represents a continuation of an earlier study on the isomeric *N*-(4-meth­oxy­phen­yl)piperazine (4-MeOPP) (Kiran Kumar *et al.*, 2020 ▸) and a range of salts and *N*-aroyl derivatives derived from 4-MeOPP (Kiran Kumar, Yathirajan, Foro *et al.*, 2019 ▸; Kiran Kumar, Yathirajan, Sagar *et al.*, 2019 ▸; Kiran Kumar *et al.*, 2020 ▸). Compounds (I)–(VI) were prepared using carbodi­imide-mediated reactions between *N*-(2-meth­oxy­phen­yl)piperazine and a halogen-substituted benzoic acid.
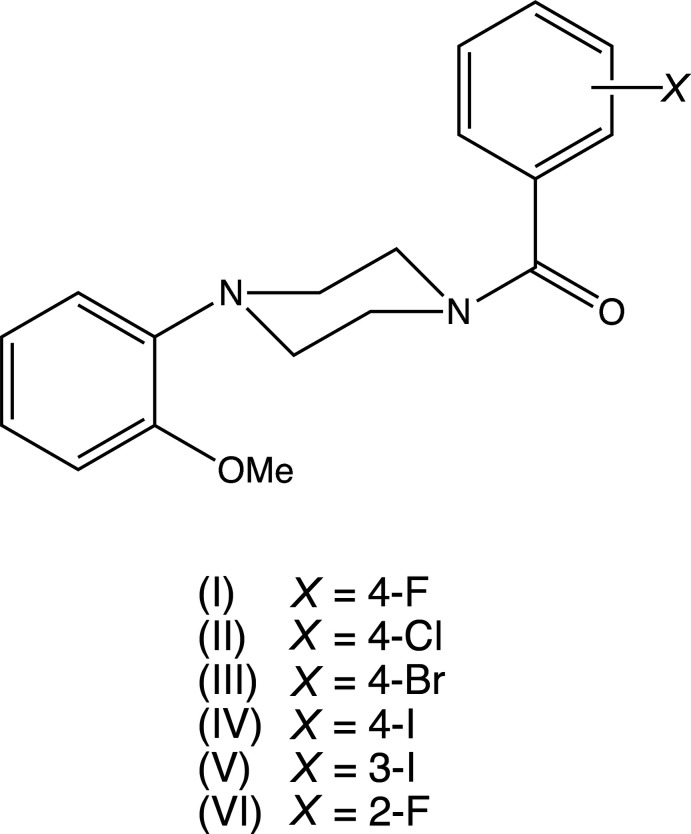


## Structural commentary   

**Figure 1:**
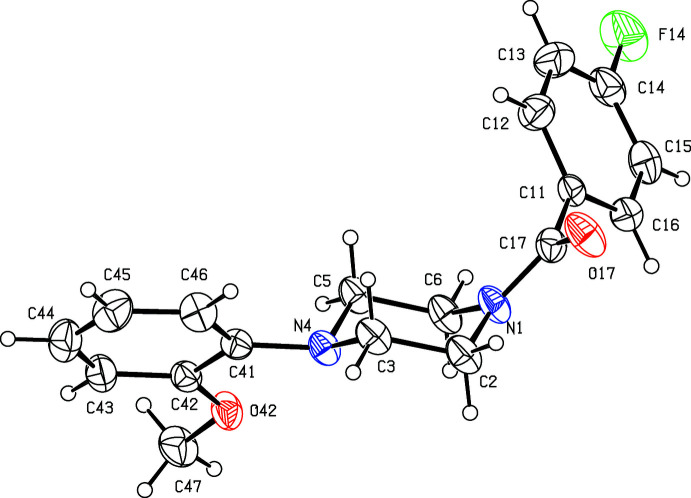
The mol­ecular structure of compound (I), showing the atom-labelling scheme. Displacement ellipsoids are drawn at the 30% probability level.

Despite differing only in the identity of their halogen substituents, no two of compounds (I)–(IV) are isomorphous (Figs. 1 ▸–6 ▸ ▸ ▸ ▸ ▸). The chloro and bromo compounds (II) and (III) both crystallize in space group *Pca*2_1_, but with *Z*′ values of 2 and 4, respectively; the unit-cell repeat vectors *b* and *c* for these two compounds are quite similar, but the *a* repeat vector for (II) is roughly twice that for (III). Compound (V) also crystallizes with *Z*′ = 2, but in space group *P*2_1_2_1_2_1_.

In none of the compounds reported here do the mol­ecules exhibit any inter­nal symmetry and hence they are conformationally chiral. The space groups for compounds (II), (III), (IV) and (VI) confirm the presence in the crystal of equal numbers of the two conformational enanti­omers. For each of (II), (III) and (V), having *Z*′ > 1, there is considerable flexibility available for the choice of the asymmetric unit: in each case, the asymmetric unit was selected such that the independent mol­ecules in it were linked by C—H⋯O hydrogen bonds (Table 1 ▸).

**Figure 2:**
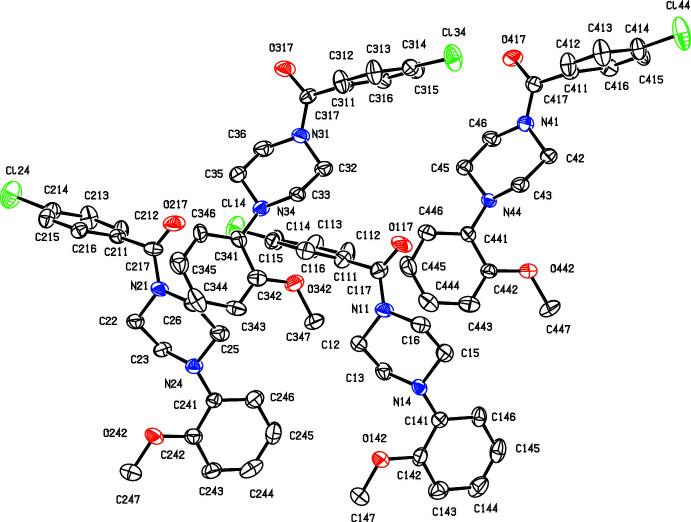
The structures of the four independent mol­ecules in the selected asymmetric unit of compound (II), viewed approximately along [001], showing the atom-labelling scheme, and the approximate spacial relationships between the mol­ecules. Displacement ellipsoids are drawn at the 30% probability level and, for the sake of clarity, the H atoms have been omitted.

**Figure 5:**
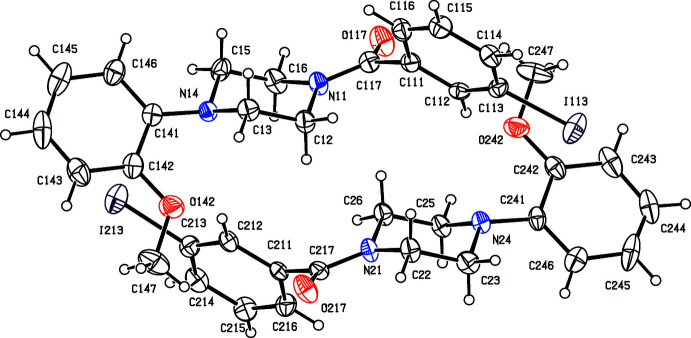
The structures of the two independent mol­ecules in the selected asymmetric unit of compound (V), showing the atom-labelling scheme and the approximate inversion symmetry relating the two mol­ecules. Displacement ellipsoids are drawn at the 30% probability level.

For compound (I), which crystallizes in space group *P*2_1_2_1_2_1_ with *Z*′ = 1, it was not possible to establish the absolute configuration of the mol­ecules in the crystal selected for data collection (see Section 6). In compound (V), the two independent mol­ecules in the selected asymmetric unit have opposite conformations and they are related by an approximate, but non-crystallographic, inversion close to (0.25, 0.60, 0.25) (*cf.* Fig. 5 ▸), and so (V) may be regarded as a kryptoracemate (Fábián & Brock, 2010 ▸). Pseudosymmetry is also apparent in compounds (II) and (III). In (III), where *Z*′ = 2, mol­ecule 1 containing atom Br14 and the major disorder component of mol­ecule 2 containing atom Br24 are related by an approximate, but non-crystallographic *b*-glide plane at *x* = *ca* 0.62 (*cf.* Fig. III). The arrangement of the mol­ecules in compound (II) is slightly more complex: mol­ecules 1 and 3, containing atoms Cl14 and Cl34, respectively, are related by an approximate, but non-crystallographic, 2_1_ screw axis along (0.56, *y*, 0.68), as also are mol­ecules 2 and 4, containing atoms Cl24 and Cl44 (*cf.* Fig. 2 ▸). In addition, mol­ecules 1 and 2 are approximately related by the translation (*x* − 0.25, *y* + 0.06, *z*), while mol­ecules 3 and 4 are approximately related by the translation (*x* + 0.25, *y* + 0.06, *z*). Compounds (II), (III) and (V) all exhibit a measure of inversion twinning (Section 6, below) and it seems likely that this is underpinned by the pseudosymmetry in these structures.

All of the piperazine rings in compounds (I)–(VI) adopt chair type conformations, with values of the ring-puckering angle θ (Cremer & Pople, 1975 ▸) close to zero, as calculated for the atom sequences (N1,C2,C3,N4,C5,C6) in (I), (IV) and (VI), or (N*x*1,C*x*2,C*x*3,N*x*4,C*x*5,C*x*6) where *x* = 1 or 2 in (III) and (V) and *x* = 1, 2, 3 or 4 in (II). For an ideal chair conformation, the value of θ is zero (Boeyens, 1978 ▸). The substituents at the N atoms all occupy equatorial sites.

In each of (I)–(IV), the meth­oxy C atom is close to coplanar with the adjacent aryl ring, with displacements from the plane of the ring ranging from 0.024 (7) Å in mol­ecule 4 of (II) to 0.130 (3) Å in (I): for (V) and (VI) the displacements are rather larger, up to 0.447 (1) Å in mol­ecule 2 of (V). However, in every mol­ecule the two exocyclic C—C—O angles differ by *ca* 10°, as typically found in planar, or near-planar, alk­oxy­arenes (Seip & Seip, 1973 ▸; Ferguson *et al.*, 1996 ▸).

## Supra­molecular features   

In assessing the inter­molecular inter­actions, we have discounted hydrogen bonds having *D*—H⋯*A* angles that are significantly less than 140°, as the inter­action energies associated with such contacts are likely to be very low, so that these cannot be regarded as structurally significant (Wood *et al.*, 2009 ▸). We have also discounted short contacts involving the H atoms of the methyl groups, as such groups are likely to be undergoing rapid rotation about the adjacent C—O bonds (Riddell & Rogerson, 1996 ▸, 1997 ▸). The C—H⋯π(arene) contacts have been included only where the H⋯*Cg* distances are less than 2.85 Å. It should perhaps be conceded here that these are somewhat arbitrary judgments, made with the primary aim of avoiding over-inter­pretation of the longer contacts and over-complication of the crystal-structure descriptions. It is convenient to consider first the supra­molecular assembly in compounds (I), (IV) and (VI) where Z′ = 1 and the aggregation is one-dimensional, followed by (III) and (V) where *Z*′ = 2 and the aggregation is two-dimensional, and finally (II) where *Z*′ = 4 and the aggregation is three-dimensional.

**Figure 7:**
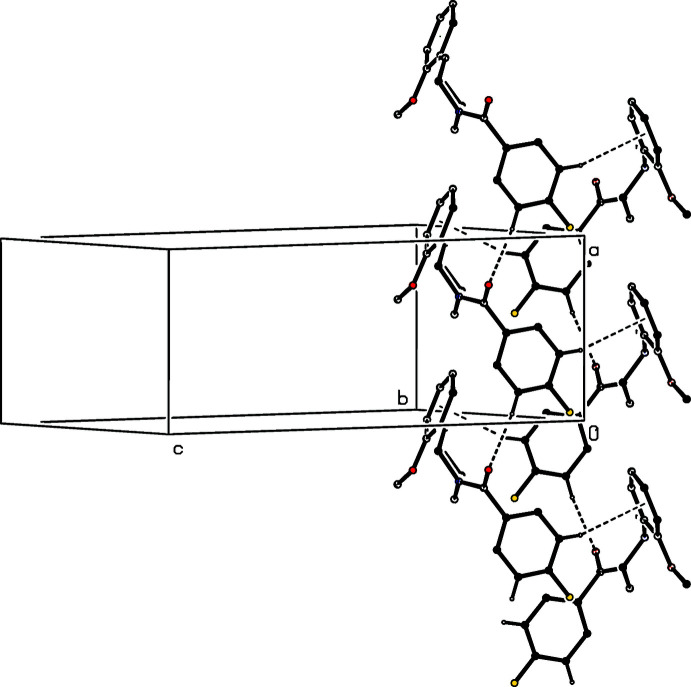
Part of the crystal structure of compound (I), showing the formation of a hydrogen-bonded chain of rings running parallel to [100]. Hydrogen bonds are drawn as dashed lines and, for the sake of clarity, the H atoms not involved in the motif shown have been omitted.

**Figure 8:**
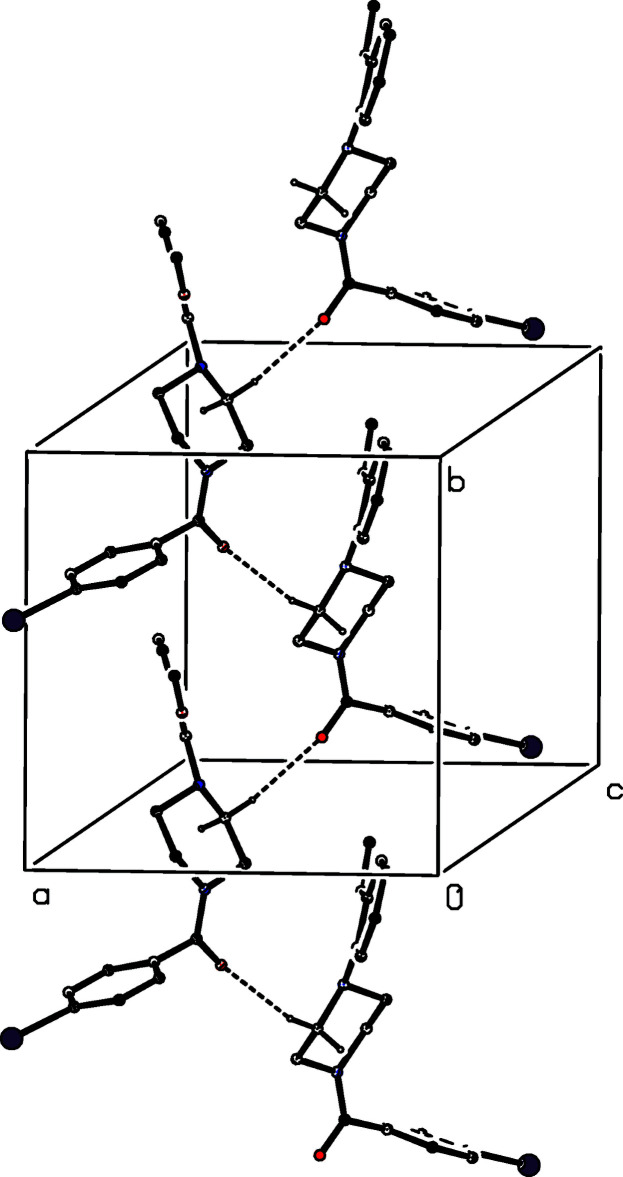
Part of the crystal structure of compound (IV), showing the formation of a hydrogen-bonded chain running parallel to [010]. Hydrogen bonds are drawn as dashed lines and, for the sake of clarity, the H atoms bonded to the C atoms which are not involved in the motif shown have been omitted.

**Figure 9:**
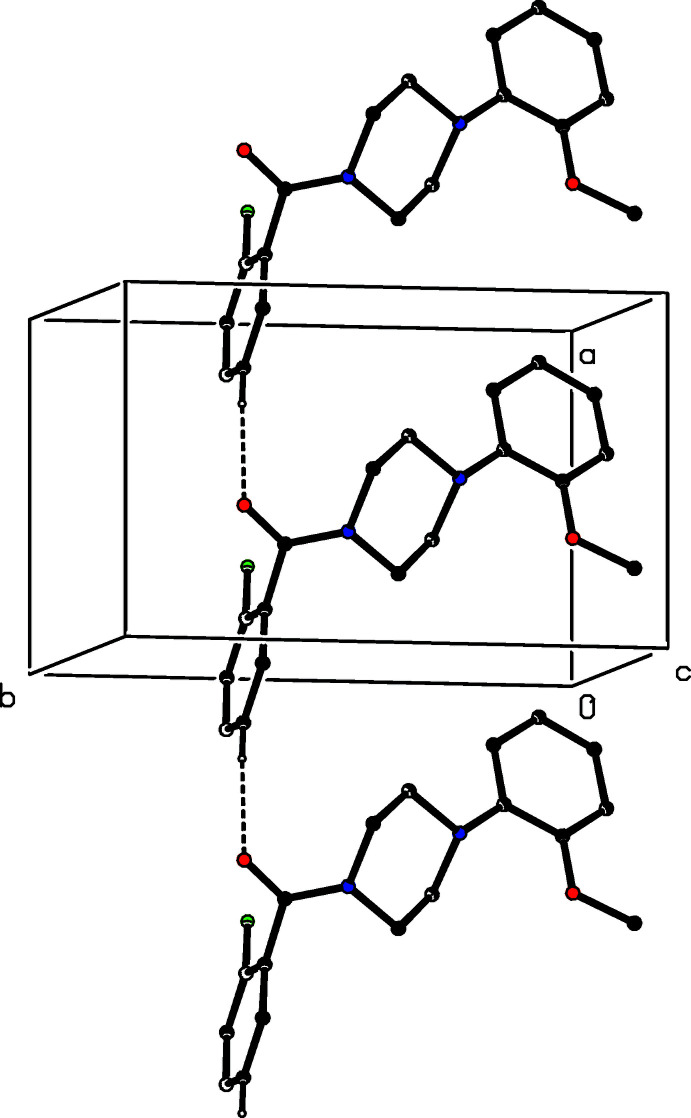
Part of the crystal structure of compound (VI), showing the formation of a hydrogen-bonded chain running parallel to [100]. Hydrogen bonds are drawn as dashed lines and, for the sake of clarity, the H atoms which are not involved in the motif shown have been omitted.

The assembly in compounds (I), (IV) and (VI) is very simple. In (I), a single C—H⋯O hydrogen bond (Table 1 ▸) links mol­ecules which are related by translation to form a *C*(6) (Etter, 1990 ▸; Etter *et al.*, 1990 ▸; Bernstein *et al.*, 1995 ▸) chain, which is weakly reinforced by a C—H⋯π(arene) hydrogen bond to form a chain of rings running along (*x*, 0.25, 0) (Fig. 7 ▸). Simple *C*(6) chains are also formed in compounds (IV) and (VI), although these involve different donors. The chain in (IV) is built from mol­ecules related by the 2_1_ screw axis along (0.5, *y*, 0.25) (Fig. 8 ▸), while that in (VI) contains mol­ecules related by translation along [100] (Fig. 9 ▸), analogous to that in (I). In none of (I), (IV) and (VI) are there any direction-specific inter­actions between adjacent chains so that, in each case, the assembly is one-dimensional.

**Figure 10:**
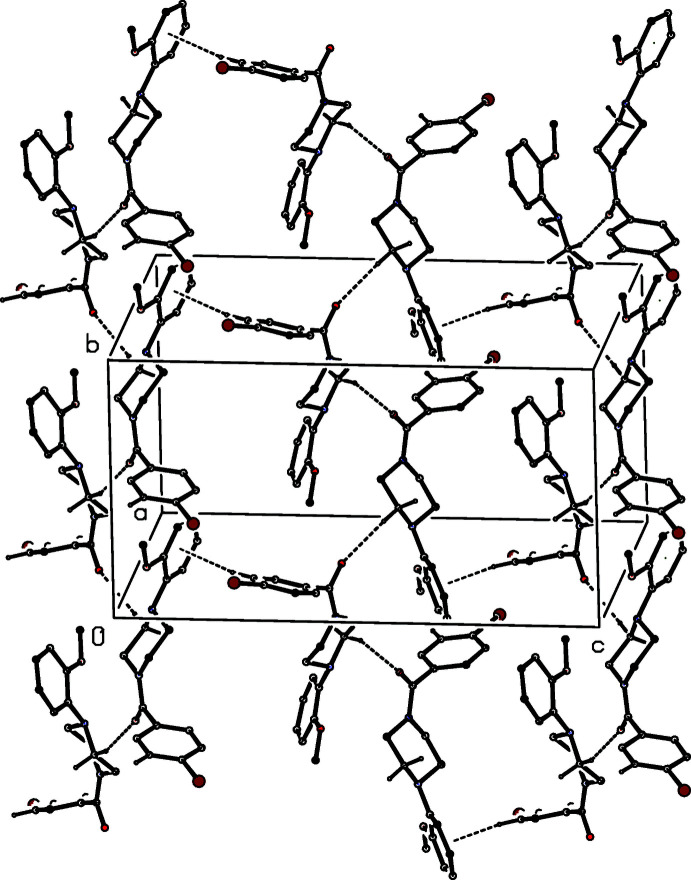
Part of the crystal structure of compound (III), showing the formation of a hydrogen-bonded sheet lying parallel to (100). Hydrogen bonds are drawn as dashed lines and, for the sake of clarity, the minor disorder component and the H atoms bonded to the C atoms which are not involved in the motif shown have been omitted.

**Figure 11:**
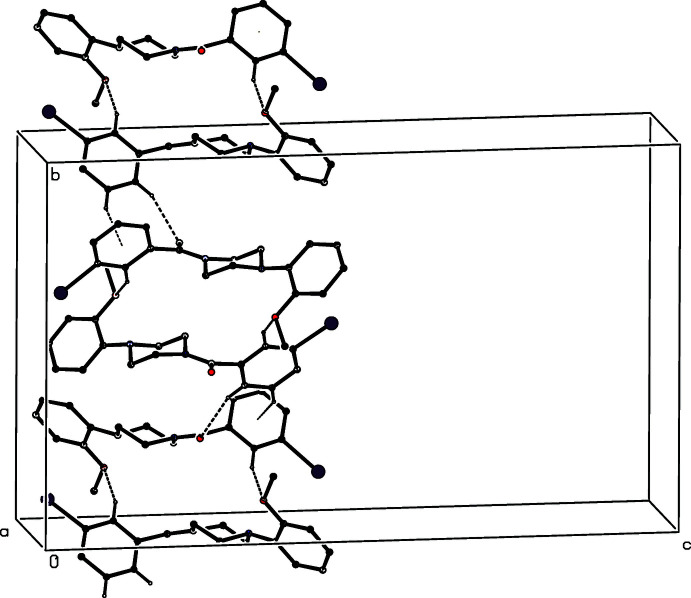
Part of the crystal structure of compound (V), showing the formation of a hydrogen-bonded chain of rings running along (1/2, *y*, 1/4). Hydrogen bonds are drawn as dashed lines and, for the sake of clarity, the H atoms which are not involved in the motif shown have been omitted.

**Figure 12:**
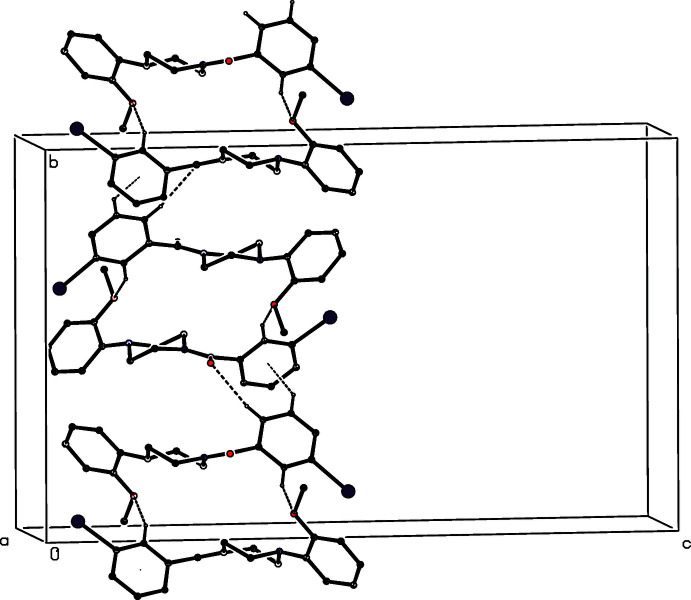
Part of the crystal structure of compound (V), showing the formation of a hydrogen-bonded chain of rings running along (0, *y*, 1/4). Hydrogen bonds are drawn as dashed lines and, for the sake of clarity, the H atoms which are not involved in the motif shown have been omitted.

Because of the very low occupancy of the minor disorder component in (III), it is necessary to consider only the inter­actions involving the major disorder component, where a combination of C—H⋯O and C—H⋯π(arene) hydrogen bonds links the mol­ecules into a sheet lying parallel to (100) (Fig. 10 ▸). The assembly in (V) is also two-dimensional, but it is rather more complex than that in (III); however, it is possible to analyse the sheet formation in (V) in terms of three simpler sub-structures (Ferguson *et al.*, 1998*a* ▸,*b* ▸; Gregson *et al.*, 2000 ▸). The first of these sub-structures, which can be regarded as the basic building block in the structure, consists of the two mol­ecules within the selected asymmetric unit (Fig. 5 ▸), which are linked by two C—H⋯O hydrogen bonds to form a cyclic dimeric unit containing an 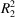
(22) motif, and dimers of this type are linked to form two types of chains of rings. One of these chains contains dimers which are related by the 2_1_ screw axis along (0.5, *y*, 0.25) (Fig. 11 ▸) and the other is built from dimers related by the 2_1_ screw axis along (0, *y*, 0.25) (Fig. 12 ▸). Within these two chains, the hydrogen bonds are directed in opposite directions (Table 1 ▸), and the combination of the two chains generates a complex sheet lying parallel to (001). There are no direction-specific inter­actions between adjacent sheets in either (III) or (V).

**Figure 13:**
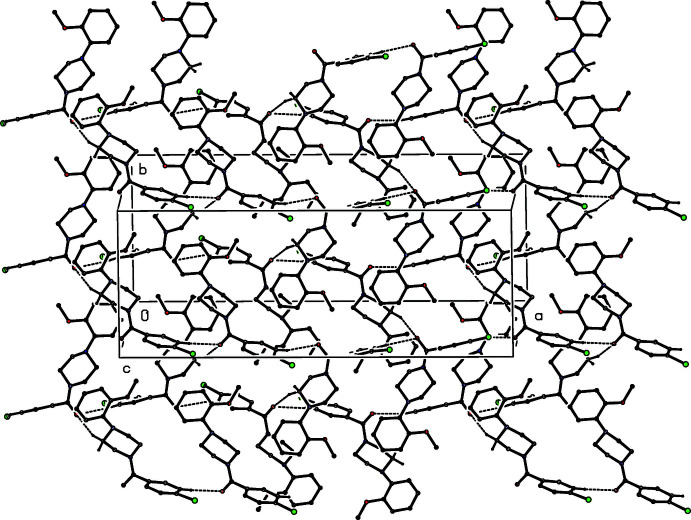
Part of the crystal structure of compound (II), showing the formation of a hydrogen-bonded sheet lying parallel to (001). Hydrogen bonds are drawn as dashed lines and, for the sake of clarity, the H atoms bonded to those C atoms which are not involved in the motif shown have been omitted.

**Figure 14:**
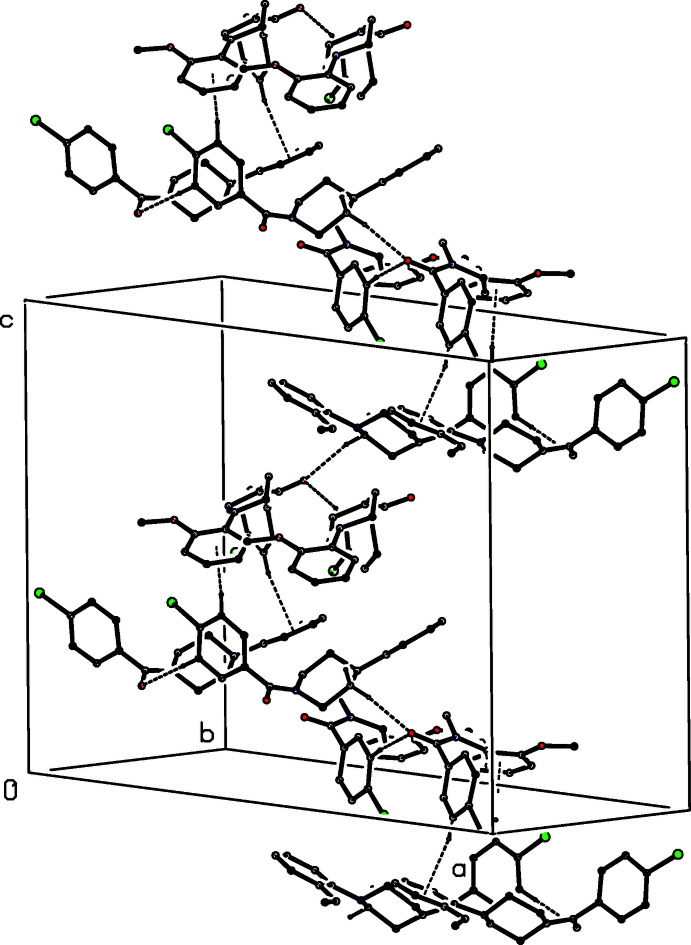
Part of the crystal structure of compound (II), showing the formation of a hydrogen-bonded chain running parallel to [001]. Hydrogen bonds are drawn as dashed lines and, for the sake of clarity, the H atoms bonded to those C atoms which are not involved in the motif shown have been omitted.

No fewer than six independent C—H⋯O hydrogen bonds, three of them within the selected asymmetric unit, link the mol­ecules of compound (II) into a complex sheet lying parallel to (001) (Fig. 13 ▸). In addition, two independent C—H⋯π(arene) hydrogen bonds link mol­ecules related by the 2_1_ screw axis along (0.5, 0.5, *z*) to generate a chain running parallel to the [001] direction (Fig. 14 ▸) and chains of this type link the (001) sheets to form a continuous three-dimensional network.

## Database survey   

Here we briefly compare the structures of compounds (I)–(VI) with those of some analogous compounds. In the structure of 1-(2-fluoro­benzo­yl)-4-(4-meth­oxy­phen­yl)piperazine (VII), which is isomeric with compound (VI) reported here, the mol­ecules are linked by two C—H⋯O hydrogen bonds to form a chain of centrosymmetric rings containing two distinct types of 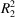
(10) ring (Kiran Kumar, Yathirajan, Sagar *et al.*, 2019 ▸). The 2-chloro, 2-bromo and 2-iodo analogues of (VII), [compounds (VIII)–(X)], are isomorphous in space group *Pbca*, all with *Z*′ = 1 (Kiran Kumar, Yathirajan, Sagar *et al.*, 2019 ▸), whereas no two of compounds (I)–(IV) reported here are isomorphous. In each of (VIII)–(X), the mol­ecules are linked into sheets by two C—H⋯π(arene) hydrogen bonds: the assembly in (VIII)–(X) thus differs markedly from that in the isomeric compounds (I)–(IV). By contrast with the assembly in (VIII)–(X), there are no significant hydrogen bonds in the structure of the unsubstituted analogue 1-benzoyl-4-(4-meth­oxy­phen­yl)piperazine (XI) (Kiran Kumar, Yathirajan, Sagar *et al.*, 2019 ▸), just as there are none in the structure of 1-(3,5-di­nitro­benzo­yl)-4-(2-meth­oxy­phen­yl)piperazine (XII) (Harish Chinthal *et al.*, 2020*b* ▸). Finally, we note that structures have been reported recently for 1-(2-iodo­benzo­yl)-4-(pyrimidin-2-yl)piperazine (Mahesha, Yathirajan *et al.*, 2019 ▸) and for three 1-(1,3-benzodioxol-5-yl)methyl-4-(halobenzo­yl)piperazines (Mahesha, Sagar *et al.*, 2019 ▸).

## Synthesis and crystallization   

All reagents were commercially available and all were used as received. For the synthesis of compounds (I)–(VI), 1-(3-di­methyl­amino­prop­yl)-3-ethyl­carbodi­imide (134 mg, 0.7 mmol), 1-hy­droxy­benzotriazole (68 mg, 0.5 mmol) and tri­ethyl­amine (0.5 ml, 1.5 mmol) were added to a solution of the appropriately substituted benzoic acid (0.52 mmol) in methanol (10 ml), thus 4-fluoro­benzoic acid (73 mg) for (I), 4-chloro­benzoic acid (82 mg) for (II), 4-bromo­benzoic acid (103 mg) for (III), 4-iodo­benzoic acid (129 mg) for (IV), 3-iodo­benzoic acid (129 mg) for (V) and 2-fluoro­benzoic acid (73 mg) for (VI). Each mixture was stirred at 323 K for a few minutes and then set aside for two days at room temperature. A solution of *N*-(2-meth­oxy­phen­yl)piperazine (100 mg, 0.52 mmol) in *N*,*N*-di­methyl­formamide (5 ml) was then added to each of the mixtures prepared as above, followed by stirring that was continued overnight at room temperature. When the reactions were confirmed to be complete using thin-layer chromatography, each mixture was then quenched with water (10 ml) and extracted with ethyl acetate (20 ml). Each organic fraction was separated and washed successively with an aqueous hydro­chloric acid solution (1 *M*), a saturated solution of sodium hydrogencarbonate and then with brine. The organic phases were dried over anhydrous sodium sulfate and the solvent was then removed under reduced pressure. The resulting solid products were then crystallized from acetone–ethyl acetate (1:1, *v*/*v*) for (I) or methanol–ethyl acetate (1:1. *v*/*v*) solvent mixtures for (II)–(VI): m.p. (I) 375–377 K, (II) 383–387 K, (III) 377–379 K, (IV) 378–381 K, (V) 379–381 K and (VI) 341–345 K. Crystals suitable for single-crystal X-ray diffraction were grown by slow evaporation, at ambient temperature and in the presence of air, of solutions in ethyl acetate.

## Refinement   

Crystal data, data collection and structure refinement details are summarized in Table 2 ▸. One bad outlier reflection (2,0,2) was omitted from the final refinement for compound (IV), and two bad outlier reflections, (1,5,18) and (1,18,15), were omitted from the final refinement for compound (V). All H atoms, apart from those in the minor disorder component of compound (III), were located in difference maps and subsequently treated as riding atoms in geometrically idealized positions, with C—H distances 0.93 Å (aromatic), 0.96 Å (CH_3_) and 0.97 Å (CH_2_), and with *U*_iso_(H) = *kU*_eq_(C), where *k* = 1.5 for the methyl groups, which were allowed to rotate but not to tilt, and 1.2 for all other H atoms. For the minor disorder component in (III), the bonded distances and the 1,3 non-bonded distances were restrained to be the same as the corresponding distances in the major disorder component, subject to s.u. values of 0.01 and 0.02 Å, respectively. In addition, the anisotropic displacement parameters for pairs of atoms occupying essentially the same physical space were constrained to be identical. Subject to these conditions, the refined disorder occupancies were 0.939 (4) and 0.061 (4). In the absence of significant resonant scattering, it was not possible to determine the absolute configuration of the mol­ecules of (I) in the crystal selected for data collection. The value of the Flack *x* parameter [Flack (1983 ▸), *x* = −0.2 (8), calculated (Parsons *et al.*, 2013 ▸) using 612 quotients of the type [(*I* ^+^) − (*I* ^−^)]/[(*I* ^+^) + (*I* ^−^)], means that the absolute structure is indeterminate (Flack & Bernardinelli, 2000 ▸), although this has no chemical significance. For each of (II), (III) and (V), the Flack *x* parameter indicated the occurrence of inversion twinning (Flack & Bernardinelli, 2000 ▸), thus: for (II), *x* = 0.22 (8) calculated using 1164 quotients; for (III), *x* = 0.300 (6) calculated using 1164 quotients; and for (V), *x* = 0.456 (12) calculated using 1728 quotients. The structure of (I) contains two void spaces, each of volume 65 Å^3^ and centred close to (0, 0.25, 0) and (0, 0.75, 0.5); however, examination of the refined structure using SQUEEZE (Spek, 2015 ▸) showed that these voids contained negligible electron density. There are four small voids in the structure of (II), each of volume *ca* 32 Å^3^, and all too small to accommodate even a water mol­ecule (Hofmann, 2002 ▸).

## Supplementary Material

Crystal structure: contains datablock(s) global, I, II, III, IV, V, VI. DOI: 10.1107/S2056989020015649/hb7954sup1.cif

Structure factors: contains datablock(s) I. DOI: 10.1107/S2056989020015649/hb7954Isup2.hkl

Structure factors: contains datablock(s) II. DOI: 10.1107/S2056989020015649/hb7954IIsup3.hkl

Structure factors: contains datablock(s) III. DOI: 10.1107/S2056989020015649/hb7954IIIsup4.hkl

Structure factors: contains datablock(s) IV. DOI: 10.1107/S2056989020015649/hb7954IVsup5.hkl

Structure factors: contains datablock(s) V. DOI: 10.1107/S2056989020015649/hb7954Vsup6.hkl

Structure factors: contains datablock(s) VI. DOI: 10.1107/S2056989020015649/hb7954VIsup7.hkl

Supporting information file. DOI: 10.1107/S2056989020015649/hb7954Isup8.cml

Supporting information file. DOI: 10.1107/S2056989020015649/hb7954IIsup9.cml

Supporting information file. DOI: 10.1107/S2056989020015649/hb7954IIIsup10.cml

Supporting information file. DOI: 10.1107/S2056989020015649/hb7954IVsup11.cml

Supporting information file. DOI: 10.1107/S2056989020015649/hb7954Vsup12.cml

Supporting information file. DOI: 10.1107/S2056989020015649/hb7954VIsup13.cml

CCDC references: 2046985, 2046984, 2046983, 2046982, 2046981, 2046980

Additional supporting information:  crystallographic information; 3D view; checkCIF report

## Figures and Tables

**Figure 3:**
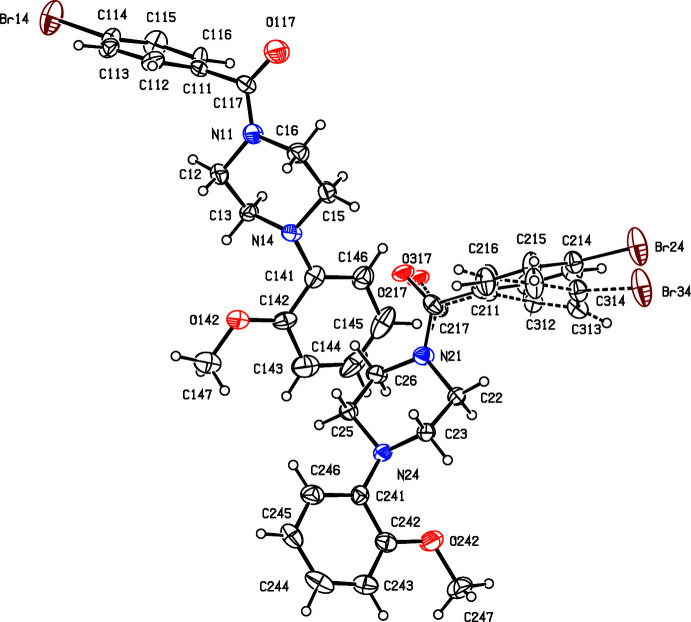
The structures of the two independent mol­ecules in the selected asymmetric unit of compound (III), viewed approximately along [001], showing the atom-labelling scheme, the disorder in one of the mol­ecules and the approximate glide relationship between the two mol­ecules. The major disorder component is drawn using full lines and the minor disorder component is drawn using broken lines: displacement ellipsoids are drawn at the 30% probability level and, for the sake of clarity, a few of the atom labels have been omitted.

**Figure 4:**
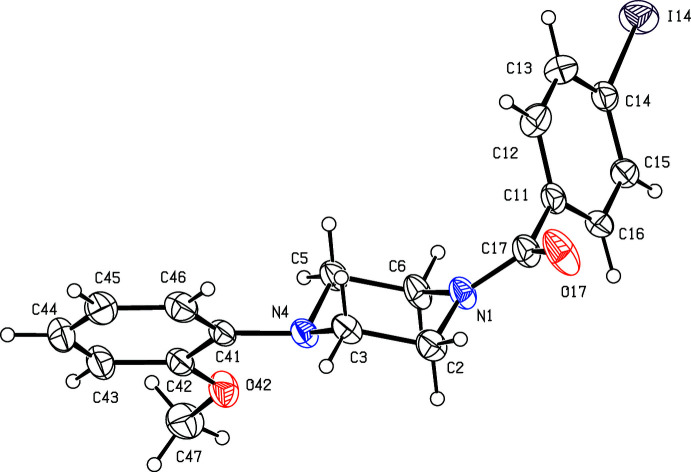
The mol­ecular structure of compound (IV), showing the atom-labelling scheme. Displacement ellipsoids are drawn at the 30% probability level.

**Figure 6:**
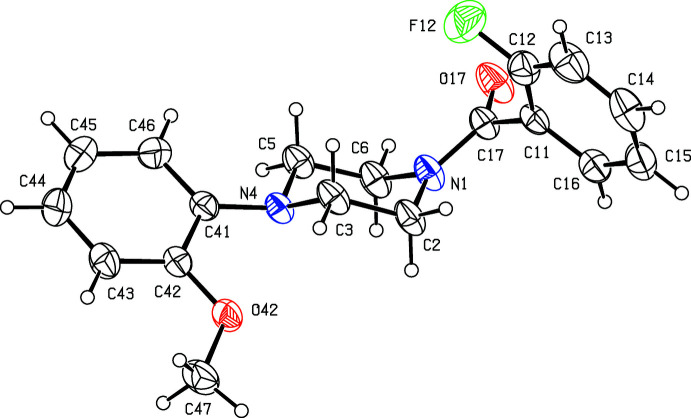
The mol­ecular structure of compound (VI), showing the atom-labelling scheme. Displacement ellipsoids are drawn at the 30% probability level.

**Table 1:** Hydrogen bonds (Å, °) *Cg*1–*Cg*7 represent the centroids of the rings (C41–C46), (C441–C446), (C241–C246), (C241–C246), (C141–C146), (C211–C216) and (C111–C116), respectively.

Compound	*D*—H⋯*A*	*D*—H	H⋯*A*	*D*⋯*A*	*D*—H⋯*A*
(I)	C15—H15⋯O17^i^	0.93	2.48	3.409 (4)	173
	C13—H13⋯*Cg*1^ii^	0.93	2.82	3.559 (4)	151
					
(II)	C15—H15*B*⋯O417^iii^	0.97	2.39	3.314 (9)	160
	C35—H365B⋯O217	0.97	2.41	3.333 (9)	159
	C115—H115⋯O217	0.93	2.60	3.522 (9)	174
	C215—H215⋯O117^iv^	0.93	2.56	3.482 (8)	170
	C315—H315⋯O417	0.93	2.56	3.486 (9)	177
	C415—H415⋯O317^v^	0.93	2.52	3.428 (8)	165
	C213—H213⋯*Cg*2^vi^	0.93	2.71	3.604 (8)	161
	C313—H313⋯*Cg*3^vii^	0.93	2.79	3.633 (8)	151
					
(III)	C15—H15*B*⋯O217	0.97	2.56	3.483 (10)	159
	C25—H25*B*⋯O115^iii^	0.97	2.55	3.483 (11)	160
	C213—H213⋯O217^viii^	0.93	2.54	3.425 (10)	158
	C312—H312⋯N14^viii^	0.93	2.59	3.45 (9)	154
	C115—H115⋯*Cg*4^ix^	0.93	2.65	3.549 (9)	162
	C315—H315⋯*Cg*5^*x*^	0.93	2.74	3.59 (10)	151
					
(IV)	C3—H3*A*⋯O17^xi^	0.97	2.50	3.422 (4)	159
					
(V)	C112—H112⋯O242	0.93	2.55	3.388 (9)	150
	C116—H116⋯O217^xii^	0.93	2.41	3.301 (10)	159
	C212—H212⋯O142	0.93	2.55	3.363 (9)	147
	C216—H216⋯O117^xiii^	0.93	2.49	3.407 (11)	169
	C115—H115⋯*Cg*6^xii^	0.93	2.67	3.505 (9)	149
	C215—H215⋯*Cg*7^xiii^	0.93	2.81	3.566 (9)	140
					
(VI)	C15—H15⋯O17^i^	0.93	2.58	3.510 (3)	177

Symmetry codes: (i) −1 + *x*, *y*, *z*; (ii) −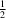 + *x*, 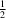 − *y*, −*z*; (iii) *x*, −1 + *y*, *z*; (iv) −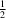 + *x*, 1 − *y*, *z*; (v) 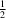 + *x*, 2 − *y*, *z*; (vi) 1 − *x*, 1 − *y*, −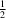 + *z*; (vii) 1 − *x*, 1 − *y*, 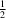 + *z*; (viii) 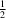 + *x*, 1 − *y*, *z*; (ix) 1 − *x*, 1 − *y*, −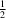 + *z*; (*x*) 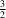 − *x*, *y*, 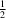 + *z*; (xi) 1 − *x*, 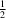 + *y*, 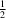 − *z*; (xii) 1 − *x*, −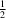 + *y*, 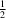 − *z*; (xiii) −*x*, 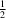 + *y*, 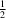 − *z*.

**Table:** 

	(I)	(II)	(III)
Crystal data			
Chemical formula	C_18_H_19_FN_2_O_2_	C_18_H_19_ClN_2_O_2_	C_18_H_19_BrN_2_O_2_
*M*_r_	314.35	330.80	375.26
Crystal system, space group	Orthorhombic, *P*2_1_2_1_2_1_	Orthorhombic, *P* *c* *a*2_1_	Orthorhombic, *P* *c* *a*2_1_
Temperature (K)	296	296	293
*a*, *b*, *c* (Å)	7.3286 (6), 11.3388 (7), 20.304 (1)	29.769 (1), 11.3173 (4), 20.4028 (8)	15.0779 (7), 11.2868 (6), 20.5297 (9)
α, β, γ (°)	90, 90, 90	90, 90, 90	90, 90, 90
*V* (Å^3^)	1687.21 (19)	6873.8 (4)	3493.8 (3)
*Z*	4	16	8
Radiation type	Mo *K*α	Mo *K*α	Mo *K*α
μ (mm^−1^)	0.09	0.23	2.36
Crystal size (mm)	0.44 × 0.14 × 0.14	0.48 × 0.38 × 0.28	0.50 × 0.48 × 0.24

Data collection			
Diffractometer	Oxford Diffraction Xcalibur CCD	Oxford Diffraction Xcalibur CCD	Oxford Diffraction Xcalibur CCD
Absorption correction	Multi-scan (*CrysAlis RED*; Oxford Diffraction, 2009 ▸)	Multi-scan (*CrysAlis RED*; Oxford Diffraction, 2009 ▸)	Multi-scan (*CrysAlis RED*; Oxford Diffraction, 2009 ▸)
*T*_min_, *T*_max_	0.938, 0.988	0.874, 0.937	0.294, 0.567
No. of measured, independent and observed [*I* > 2σ(*I*)] reflections	6377, 3377, 1918	17985, 8328, 4822	13342, 5910, 3300
*R*_int_	0.036	0.030	0.031
(sin θ/λ)_max_ (Å^−1^)	0.628	0.606	0.606

Refinement			
*R*[*F* ^2^ > 2σ(*F* ^2^)], *wR*(*F* ^2^), *S*	0.044, 0.088, 0.92	0.055, 0.143, 0.95	0.051, 0.130, 0.93
No. of reflections	3377	8328	5910
No. of parameters	209	833	445
No. of restraints	0	1	21
H-atom treatment	H-atom parameters constrained	H-atom parameters constrained	H-atom parameters constrained
Δρ_max_, Δρ_min_ (e Å^−3^)	0.12, −0.14	0.52, −0.19	0.85, −0.49
Absolute structure	–	Flack *x* determined using 1164 quotients [(*I* ^+^)−(*I* ^−^)]/[(*I* ^+^)+(*I* ^−^)] (Parsons *et al.*, 2013 ▸)	Flack *x* determined using 1109 quotients [(*I* ^+^)−(*I* ^−^)]/[(*I* ^+^)+(*I* ^−^)] (Parsons *et al.*, 2013 ▸)
Absolute structure parameter	–	0.22 (6)	0.300 (6)

**Table:** 

	(IV)	(V)	(VI)
Crystal data			
Chemical formula	C_18_H_19_IN_2_O_2_	C_18_H_19_IN_2_O_2_	C_18_H_19_FN_2_O_2_
*M*_r_	422.25	422.25	314.35
Crystal system, space group	Monoclinic, *P*2_1_/*c*	Orthorhombic, *P*2_1_2_1_2_1_	Monoclinic, *P*2_1_/*n*
Temperature (K)	296	296	296
*a*, *b*, *c* (Å)	10.9626 (5), 11.3258 (6), 14.8234 (7)	7.4528 (4), 17.1306 (9), 27.903 (1)	7.451 (1), 11.199 (3), 19.138 (5)
α, β, γ (°)	90, 104.520 (5), 90	90, 90, 90	90, 99.59 (2), 90
*V* (Å^3^)	1781.69 (16)	3562.4 (3)	1574.6 (6)
*Z*	4	8	4
Radiation type	Mo *K*α	Mo *K*α	Mo *K*α
μ (mm^−1^)	1.81	1.81	0.10
Crystal size (mm)	0.42 × 0.40 × 0.28	0.36 × 0.22 × 0.18	0.48 × 0.48 × 0.24

Data collection			
Diffractometer	Oxford Diffraction Xcalibur CCD	Oxford Diffraction Xcalibur CCD	Oxford Diffraction Xcalibur CCD
Absorption correction	Multi-scan (*CrysAlis RED*; Oxford Diffraction, 2009 ▸)	Multi-scan (*CrysAlis RED*; Oxford Diffraction, 2009 ▸)	Multi-scan (*CrysAlis RED*; Oxford Diffraction, 2009 ▸)
*T*_min_, *T*_max_	0.423, 0.603	0.542, 0.722	0.898, 0.955
No. of measured, independent and observed [*I* > 2σ(*I*)] reflections	7512, 3816, 2690	13774, 7653, 5048	6456, 3467, 2081
*R*_int_	0.015	0.025	0.025
(sin θ/λ)_max_ (Å^−1^)	0.651	0.650	0.659

Refinement			
*R*[*F* ^2^ > 2σ(*F* ^2^)], *wR*(*F* ^2^), *S*	0.035, 0.092, 1.05	0.048, 0.116, 1.04	0.049, 0.128, 1.02
No. of reflections	3816	7653	3467
No. of parameters	208	417	208
No. of restraints	0	0	0
H-atom treatment	H-atom parameters constrained	H-atom parameters constrained	H-atom parameters constrained
Δρ_max_, Δρ_min_ (e Å^−3^)	0.43, −0.91	1.12, −0.69	0.17, −0.17
Absolute structure	–	Flack *x* determined using 1728 quotients [(*I* ^+^)−(*I* ^−^)]/[(*I* ^+^)+(*I* ^−^)] (Parsons *et al.*, 2013 ▸)	–
Absolute structure parameter	–	0.456 (12)	–

Computer programs: *CrysAlis CCD* and *CrysAlis RED* (Oxford Diffraction, 2009 ▸), *SHELXT* (Sheldrick, 2015*a* ▸), *SHELXL2014* (Sheldrick, 2015*b* ▸) and *PLATON* (Spek, 2020 ▸).

## supplementary crystallographic information

### 1-(4-Fluorobenzoyl)-4-(2-methoxyphenyl)piperazine (I) Crystal data

**Table:**

C_18_H_19_FN_2_O_2_	*D*_x_ = 1.238 Mg m^−^^3^
*M**_r_* = 314.35	Mo *K*α radiation, λ = 0.71073 Å
Orthorhombic, *P*2_1_2_1_2_1_	Cell parameters from 3523 reflections
*a* = 7.3286 (6) Å	θ = 3.0–27.8°
*b* = 11.3388 (7) Å	µ = 0.09 mm^−^^1^
*c* = 20.304 (1) Å	*T* = 296 K
*V* = 1687.21 (19) Å^3^	Needle, yellow
*Z* = 4	0.44 × 0.14 × 0.14 mm
*F*(000) = 664	

### 1-(4-Fluorobenzoyl)-4-(2-methoxyphenyl)piperazine (I) Data collection

**Table:**

Oxford Diffraction Xcalibur CCD diffractometer	3377 independent reflections
Radiation source: Enhance (Mo) X-ray Source	1918 reflections with *I* > 2σ(*I*)
Graphite monochromator	*R*_int_ = 0.036
ω scans	θ_max_ = 26.5°, θ_min_ = 3.0°
Absorption correction: multi-scan (CrysalisRed; Oxford Diffraction, 2009)	*h* = −8→9
*T*_min_ = 0.938, *T*_max_ = 0.988	*k* = −10→14
6377 measured reflections	*l* = −16→25

### 1-(4-Fluorobenzoyl)-4-(2-methoxyphenyl)piperazine (I) Refinement

**Table:**

Refinement on *F*^2^	Primary atom site location: difference Fourier map
Least-squares matrix: full	Hydrogen site location: inferred from neighbouring sites
*R*[*F*^2^ > 2σ(*F*^2^)] = 0.044	H-atom parameters constrained
*wR*(*F*^2^) = 0.088	*w* = 1/[σ^2^(*F*_o_^2^) + (0.0379*P*)^2^] where *P* = (*F*_o_^2^ + 2*F*_c_^2^)/3
*S* = 0.92	(Δ/σ)_max_ < 0.001
3377 reflections	Δρ_max_ = 0.12 e Å^−^^3^
209 parameters	Δρ_min_ = −0.14 e Å^−^^3^
0 restraints	

### 1-(4-Fluorobenzoyl)-4-(2-methoxyphenyl)piperazine (I) Special details

**Table:**

Geometry. All esds (except the esd in the dihedral angle between two l.s. planes) are estimated using the full covariance matrix. The cell esds are taken into account individually in the estimation of esds in distances, angles and torsion angles; correlations between esds in cell parameters are only used when they are defined by crystal symmetry. An approximate (isotropic) treatment of cell esds is used for estimating esds involving l.s. planes.

### 1-(4-Fluorobenzoyl)-4-(2-methoxyphenyl)piperazine (I) Fractional atomic coordinates and isotropic or equivalent isotropic displacement parameters (Å^2^)

**Table:**

	*x*	*y*	*z*	*U*_iso_*/*U*_eq_	
N1	0.6727 (4)	0.2259 (2)	0.20993 (12)	0.0536 (7)	
C2	0.8380 (5)	0.2651 (3)	0.24390 (16)	0.0595 (9)	
H2A	0.9060	0.1970	0.2591	0.071*	
H2B	0.8046	0.3118	0.2821	0.071*	
C3	0.9566 (4)	0.3381 (3)	0.19839 (14)	0.0538 (9)	
H3A	1.0612	0.3678	0.2225	0.065*	
H3B	1.0010	0.2890	0.1627	0.065*	
N4	0.8517 (3)	0.4376 (2)	0.17130 (11)	0.0465 (7)	
C5	0.6898 (4)	0.3946 (3)	0.13597 (15)	0.0525 (9)	
H5A	0.7276	0.3458	0.0992	0.063*	
H5B	0.6219	0.4612	0.1186	0.063*	
C6	0.5689 (4)	0.3238 (3)	0.18136 (16)	0.0563 (9)	
H6A	0.5229	0.3741	0.2163	0.068*	
H6B	0.4654	0.2932	0.1569	0.068*	
C17	0.6446 (5)	0.1113 (3)	0.19657 (14)	0.0478 (8)	
O17	0.7472 (3)	0.03302 (19)	0.21678 (12)	0.0737 (7)	
C11	0.4817 (4)	0.0807 (2)	0.15457 (14)	0.0425 (7)	
C12	0.5086 (5)	0.0571 (3)	0.08810 (16)	0.0624 (10)	
H12	0.6255	0.0608	0.0704	0.075*	
C13	0.3629 (6)	0.0284 (4)	0.04822 (17)	0.0740 (11)	
H13	0.3799	0.0132	0.0036	0.089*	
C14	0.1935 (5)	0.0228 (3)	0.07572 (19)	0.0656 (10)	
F14	0.0480 (3)	−0.0036 (2)	0.03598 (11)	0.1066 (9)	
C15	0.1623 (5)	0.0423 (3)	0.14120 (19)	0.0620 (9)	
H15	0.0455	0.0361	0.1588	0.074*	
C16	0.3097 (5)	0.0716 (3)	0.18063 (16)	0.0530 (8)	
H16	0.2918	0.0852	0.2253	0.064*	
C41	0.9543 (4)	0.5233 (3)	0.13506 (13)	0.0445 (7)	
C42	0.8745 (4)	0.6348 (3)	0.12306 (14)	0.0450 (8)	
C43	0.9689 (5)	0.7194 (3)	0.08778 (15)	0.0568 (9)	
H43	0.9146	0.7918	0.0791	0.068*	
C44	1.1429 (6)	0.6969 (3)	0.06546 (16)	0.0696 (11)	
H44	1.2055	0.7544	0.0419	0.083*	
C45	1.2242 (5)	0.5907 (4)	0.07779 (17)	0.0719 (11)	
H45	1.3425	0.5763	0.0633	0.086*	
C46	1.1291 (4)	0.5041 (3)	0.11218 (16)	0.0609 (9)	
H46	1.1847	0.4317	0.1199	0.073*	
O42	0.7039 (3)	0.65179 (17)	0.14915 (10)	0.0576 (6)	
C47	0.6206 (5)	0.7644 (3)	0.14019 (17)	0.0716 (11)	
H47A	0.5031	0.7649	0.1611	0.107*	
H47B	0.6063	0.7797	0.0940	0.107*	
H47C	0.6962	0.8242	0.1595	0.107*	

### 1-(4-Fluorobenzoyl)-4-(2-methoxyphenyl)piperazine (I) Atomic displacement parameters (Å^2^)

**Table:**

	*U*^11^	*U*^22^	*U*^33^	*U*^12^	*U*^13^	*U*^23^
N1	0.0602 (19)	0.0310 (14)	0.0697 (18)	−0.0018 (14)	−0.0176 (15)	0.0067 (12)
C2	0.064 (2)	0.0433 (18)	0.071 (2)	−0.0053 (18)	−0.0241 (19)	0.0046 (17)
C3	0.053 (2)	0.0427 (19)	0.066 (2)	0.0021 (17)	−0.0190 (17)	0.0030 (16)
N4	0.0440 (16)	0.0376 (14)	0.0580 (15)	0.0012 (13)	−0.0101 (14)	0.0038 (12)
C5	0.052 (2)	0.0403 (17)	0.065 (2)	0.0001 (16)	−0.0163 (18)	0.0060 (15)
C6	0.055 (2)	0.0352 (18)	0.079 (2)	0.0003 (17)	−0.0104 (18)	0.0036 (16)
C17	0.051 (2)	0.0403 (19)	0.0524 (19)	0.0035 (18)	0.0005 (16)	0.0064 (14)
O17	0.0726 (17)	0.0409 (13)	0.108 (2)	0.0047 (13)	−0.0297 (14)	0.0048 (12)
C11	0.048 (2)	0.0314 (16)	0.0484 (19)	−0.0022 (14)	−0.0011 (16)	0.0033 (13)
C12	0.061 (2)	0.070 (2)	0.056 (2)	−0.0033 (19)	0.0045 (19)	−0.0008 (17)
C13	0.085 (3)	0.085 (3)	0.051 (2)	−0.010 (3)	−0.006 (2)	−0.0045 (19)
C14	0.072 (3)	0.055 (2)	0.070 (3)	−0.016 (2)	−0.029 (2)	0.0045 (19)
F14	0.1058 (18)	0.1026 (19)	0.1113 (17)	−0.0293 (16)	−0.0557 (15)	0.0085 (15)
C15	0.047 (2)	0.053 (2)	0.086 (3)	−0.0059 (18)	0.000 (2)	0.0089 (18)
C16	0.054 (2)	0.051 (2)	0.0537 (19)	−0.0048 (17)	0.0025 (18)	−0.0015 (15)
C41	0.0445 (18)	0.0440 (19)	0.0451 (17)	−0.0018 (17)	−0.0018 (15)	−0.0028 (15)
C42	0.044 (2)	0.0434 (19)	0.0480 (17)	−0.0050 (16)	−0.0010 (16)	−0.0031 (15)
C43	0.059 (2)	0.051 (2)	0.061 (2)	−0.0106 (19)	−0.0007 (19)	0.0067 (17)
C44	0.062 (3)	0.074 (3)	0.072 (2)	−0.015 (2)	0.005 (2)	0.0121 (19)
C45	0.046 (2)	0.092 (3)	0.078 (3)	−0.002 (2)	0.013 (2)	0.000 (2)
C46	0.051 (2)	0.060 (2)	0.071 (2)	0.0048 (19)	−0.0026 (18)	0.0027 (19)
O42	0.0617 (16)	0.0372 (12)	0.0739 (15)	0.0044 (11)	0.0126 (13)	0.0058 (10)
C47	0.076 (3)	0.045 (2)	0.093 (3)	0.0152 (19)	0.010 (2)	−0.0019 (18)

### 1-(4-Fluorobenzoyl)-4-(2-methoxyphenyl)piperazine (I) Geometric parameters (Å, º)

**Table:**

N1—C17	1.343 (4)	C13—C14	1.363 (5)
N1—C2	1.463 (4)	C13—H13	0.9300
N1—C6	1.466 (4)	C14—C15	1.367 (5)
C2—C3	1.515 (4)	C14—F14	1.370 (4)
C2—H2A	0.9700	C15—C16	1.385 (4)
C2—H2B	0.9700	C15—H15	0.9300
C3—N4	1.472 (3)	C16—H16	0.9300
C3—H3A	0.9700	C41—C46	1.380 (4)
C3—H3B	0.9700	C41—C42	1.414 (4)
N4—C41	1.432 (4)	C42—O42	1.372 (3)
N4—C5	1.470 (4)	C42—C43	1.383 (4)
C5—C6	1.510 (4)	C43—C44	1.377 (5)
C5—H5A	0.9700	C43—H43	0.9300
C5—H5B	0.9700	C44—C45	1.367 (5)
C6—H6A	0.9700	C44—H44	0.9300
C6—H6B	0.9700	C45—C46	1.392 (4)
C17—O17	1.233 (3)	C45—H45	0.9300
C17—C11	1.508 (4)	C46—H46	0.9300
C11—C16	1.371 (4)	O42—C47	1.427 (4)
C11—C12	1.390 (4)	C47—H47A	0.9600
C12—C13	1.379 (5)	C47—H47B	0.9600
C12—H12	0.9300	C47—H47C	0.9600
			
C17—N1—C2	121.1 (3)	C11—C12—H12	119.8
C17—N1—C6	125.0 (3)	C14—C13—C12	118.4 (3)
C2—N1—C6	112.7 (2)	C14—C13—H13	120.8
N1—C2—C3	110.7 (3)	C12—C13—H13	120.8
N1—C2—H2A	109.5	C13—C14—C15	122.9 (3)
C3—C2—H2A	109.5	C13—C14—F14	118.5 (3)
N1—C2—H2B	109.5	C15—C14—F14	118.6 (4)
C3—C2—H2B	109.5	C14—C15—C16	118.1 (3)
H2A—C2—H2B	108.1	C14—C15—H15	121.0
N4—C3—C2	110.3 (3)	C16—C15—H15	121.0
N4—C3—H3A	109.6	C11—C16—C15	120.8 (3)
C2—C3—H3A	109.6	C11—C16—H16	119.6
N4—C3—H3B	109.6	C15—C16—H16	119.6
C2—C3—H3B	109.6	C46—C41—C42	117.8 (3)
H3A—C3—H3B	108.1	C46—C41—N4	123.6 (3)
C41—N4—C5	113.4 (2)	C42—C41—N4	118.6 (3)
C41—N4—C3	116.0 (2)	O42—C42—C43	124.0 (3)
C5—N4—C3	110.5 (2)	O42—C42—C41	115.8 (2)
N4—C5—C6	110.6 (2)	C43—C42—C41	120.2 (3)
N4—C5—H5A	109.5	C44—C43—C42	120.4 (3)
C6—C5—H5A	109.5	C44—C43—H43	119.8
N4—C5—H5B	109.5	C42—C43—H43	119.8
C6—C5—H5B	109.5	C45—C44—C43	120.4 (3)
H5A—C5—H5B	108.1	C45—C44—H44	119.8
N1—C6—C5	109.9 (3)	C43—C44—H44	119.8
N1—C6—H6A	109.7	C44—C45—C46	119.7 (3)
C5—C6—H6A	109.7	C44—C45—H45	120.2
N1—C6—H6B	109.7	C46—C45—H45	120.2
C5—C6—H6B	109.7	C41—C46—C45	121.5 (3)
H6A—C6—H6B	108.2	C41—C46—H46	119.2
O17—C17—N1	122.4 (3)	C45—C46—H46	119.2
O17—C17—C11	120.4 (3)	C42—O42—C47	117.8 (2)
N1—C17—C11	117.3 (3)	O42—C47—H47A	109.5
C16—C11—C12	119.4 (3)	O42—C47—H47B	109.5
C16—C11—C17	121.8 (3)	H47A—C47—H47B	109.5
C12—C11—C17	118.8 (3)	O42—C47—H47C	109.5
C13—C12—C11	120.4 (3)	H47A—C47—H47C	109.5
C13—C12—H12	119.8	H47B—C47—H47C	109.5
			
C17—N1—C2—C3	112.8 (3)	C13—C14—C15—C16	1.5 (5)
C6—N1—C2—C3	−55.1 (3)	F14—C14—C15—C16	−178.6 (3)
N1—C2—C3—N4	55.4 (3)	C12—C11—C16—C15	−1.7 (4)
C2—C3—N4—C41	171.3 (2)	C17—C11—C16—C15	−179.4 (3)
C2—C3—N4—C5	−57.8 (3)	C14—C15—C16—C11	0.0 (5)
C41—N4—C5—C6	−168.9 (2)	C5—N4—C41—C46	−114.2 (3)
C3—N4—C5—C6	58.9 (3)	C3—N4—C41—C46	15.3 (4)
C17—N1—C6—C5	−111.8 (3)	C5—N4—C41—C42	67.2 (3)
C2—N1—C6—C5	55.5 (3)	C3—N4—C41—C42	−163.3 (2)
N4—C5—C6—N1	−56.9 (3)	C46—C41—C42—O42	−177.2 (2)
C2—N1—C17—O17	6.3 (5)	N4—C41—C42—O42	1.4 (4)
C6—N1—C17—O17	172.6 (3)	C46—C41—C42—C43	1.9 (4)
C2—N1—C17—C11	−173.0 (3)	N4—C41—C42—C43	−179.5 (3)
C6—N1—C17—C11	−6.7 (4)	O42—C42—C43—C44	177.4 (3)
O17—C17—C11—C16	98.8 (4)	C41—C42—C43—C44	−1.6 (5)
N1—C17—C11—C16	−81.9 (3)	C42—C43—C44—C45	0.2 (5)
O17—C17—C11—C12	−79.0 (4)	C43—C44—C45—C46	1.1 (5)
N1—C17—C11—C12	100.3 (3)	C42—C41—C46—C45	−0.7 (4)
C16—C11—C12—C13	2.0 (5)	N4—C41—C46—C45	−179.3 (3)
C17—C11—C12—C13	179.7 (3)	C44—C45—C46—C41	−0.8 (5)
C11—C12—C13—C14	−0.5 (5)	C43—C42—O42—C47	−1.1 (4)
C12—C13—C14—C15	−1.3 (6)	C41—C42—O42—C47	177.9 (3)
C12—C13—C14—F14	178.8 (3)		

### 1-(4-Fluorobenzoyl)-4-(2-methoxyphenyl)piperazine (I) Hydrogen-bond geometry (Å, º)

**Table:**

*D*—H···*A*	*D*—H	H···*A*	*D*···*A*	*D*—H···*A*
C15—H15···O17^i^	0.93	2.48	3.409 (4)	173
C13—H13···*Cg*1^ii^	0.93	2.82	3.559 (4)	151

Symmetry codes: (i) *x*−1, *y*, *z*; (ii) *x*−1/2, −*y*+1/2, −*z*.

### 1-(4-Chlorobenzoyl)-4-(2-methoxyphenyl)piperazine (II) Crystal data

**Table:**

C_18_H_19_ClN_2_O_2_	*D*_x_ = 1.279 Mg m^−^^3^
*M**_r_* = 330.80	Mo *K*α radiation, λ = 0.71073 Å
Orthorhombic, *P**c**a*2_1_	Cell parameters from 9521 reflections
*a* = 29.769 (1) Å	θ = 2.7–28.0°
*b* = 11.3173 (4) Å	µ = 0.23 mm^−^^1^
*c* = 20.4028 (8) Å	*T* = 296 K
*V* = 6873.8 (4) Å^3^	Block, yellow
*Z* = 16	0.48 × 0.38 × 0.28 mm
*F*(000) = 2784	

### 1-(4-Chlorobenzoyl)-4-(2-methoxyphenyl)piperazine (II) Data collection

**Table:**

Oxford Diffraction Xcalibur CCD diffractometer	8328 independent reflections
Radiation source: Enhance (Mo) X-ray Source	4822 reflections with *I* > 2σ(*I*)
Graphite monochromator	*R*_int_ = 0.030
ω scans	θ_max_ = 25.5°, θ_min_ = 2.7°
Absorption correction: multi-scan (CrysalisRed; Oxford Diffraction, 2009)	*h* = −36→16
*T*_min_ = 0.874, *T*_max_ = 0.937	*k* = −13→10
17985 measured reflections	*l* = −24→14

### 1-(4-Chlorobenzoyl)-4-(2-methoxyphenyl)piperazine (II) Refinement

**Table:**

Refinement on *F*^2^	Hydrogen site location: inferred from neighbouring sites
Least-squares matrix: full	H-atom parameters constrained
*R*[*F*^2^ > 2σ(*F*^2^)] = 0.055	*w* = 1/[σ^2^(*F*_o_^2^) + (0.0848*P*)^2^] where *P* = (*F*_o_^2^ + 2*F*_c_^2^)/3
*wR*(*F*^2^) = 0.143	(Δ/σ)_max_ < 0.001
*S* = 0.95	Δρ_max_ = 0.52 e Å^−^^3^
8328 reflections	Δρ_min_ = −0.19 e Å^−^^3^
833 parameters	Absolute structure: Flack *x* determined using 1164 quotients [(*I*^+^)-(*I*^-^)]/[(*I*^+^)+(*I*^-^)] (Parsons *et al.*, 2013)
1 restraint	Absolute structure parameter: 0.22 (6)
Primary atom site location: difference Fourier map	

### 1-(4-Chlorobenzoyl)-4-(2-methoxyphenyl)piperazine (II) Special details

**Table:**

Geometry. All esds (except the esd in the dihedral angle between two l.s. planes) are estimated using the full covariance matrix. The cell esds are taken into account individually in the estimation of esds in distances, angles and torsion angles; correlations between esds in cell parameters are only used when they are defined by crystal symmetry. An approximate (isotropic) treatment of cell esds is used for estimating esds involving l.s. planes.

### 1-(4-Chlorobenzoyl)-4-(2-methoxyphenyl)piperazine (II) Fractional atomic coordinates and isotropic or equivalent isotropic displacement parameters (Å^2^)

**Table:**

	*x*	*y*	*z*	*U*_iso_*/*U*_eq_	
N11	0.60851 (17)	0.2852 (4)	0.6321 (3)	0.0554 (15)	
C12	0.5808 (2)	0.1870 (5)	0.6056 (4)	0.056 (2)	
H12A	0.5682	0.1417	0.6416	0.068*	
H12B	0.5561	0.2191	0.5802	0.068*	
C13	0.6084 (2)	0.1091 (6)	0.5639 (3)	0.0554 (19)	
H13A	0.5902	0.0428	0.5494	0.067*	
H13B	0.6178	0.1525	0.5252	0.067*	
N14	0.64833 (16)	0.0640 (4)	0.5981 (3)	0.0459 (14)	
C15	0.6758 (2)	0.1638 (6)	0.6203 (4)	0.0590 (19)	
H15A	0.6862	0.2084	0.5827	0.071*	
H15B	0.7019	0.1344	0.6436	0.071*	
C16	0.6492 (2)	0.2432 (5)	0.6646 (3)	0.0583 (19)	
H16A	0.6410	0.2004	0.7040	0.070*	
H16B	0.6675	0.3102	0.6772	0.070*	
C117	0.6019 (2)	0.4008 (6)	0.6206 (3)	0.0529 (17)	
O117	0.62648 (18)	0.4766 (4)	0.6393 (3)	0.0773 (18)	
C111	0.5609 (2)	0.4314 (5)	0.5810 (3)	0.0476 (16)	
C112	0.5649 (2)	0.4468 (7)	0.5130 (4)	0.069 (2)	
H112	0.5928	0.4405	0.4930	0.082*	
C113	0.5280 (3)	0.4709 (7)	0.4764 (4)	0.071 (2)	
H113	0.5303	0.4765	0.4311	0.085*	
C114	0.4866 (2)	0.4871 (6)	0.5067 (4)	0.0528 (19)	
Cl14	0.44067 (7)	0.5176 (2)	0.45801 (12)	0.0904 (7)	
C115	0.4828 (2)	0.4822 (5)	0.5729 (4)	0.0481 (19)	
H115	0.4554	0.4975	0.5931	0.058*	
C116	0.5202 (2)	0.4541 (5)	0.6099 (3)	0.0522 (18)	
H116	0.5178	0.4505	0.6553	0.063*	
C141	0.6709 (2)	−0.0286 (5)	0.5655 (4)	0.0430 (17)	
C142	0.6498 (2)	−0.1368 (6)	0.5593 (4)	0.0547 (19)	
C143	0.6705 (2)	−0.2321 (6)	0.5287 (4)	0.067 (2)	
H143	0.6552	−0.3031	0.5232	0.081*	
C144	0.7142 (3)	−0.2195 (7)	0.5063 (4)	0.077 (2)	
H144	0.7291	−0.2830	0.4871	0.092*	
C145	0.7351 (2)	−0.1137 (8)	0.5128 (4)	0.071 (2)	
H145	0.7641	−0.1045	0.4965	0.085*	
C146	0.7139 (2)	−0.0171 (6)	0.5437 (4)	0.054 (2)	
H146	0.7291	0.0539	0.5492	0.065*	
O142	0.60683 (14)	−0.1474 (3)	0.5834 (2)	0.0616 (13)	
C147	0.5844 (2)	−0.2559 (6)	0.5776 (4)	0.072 (2)	
H17A	0.6016	−0.3164	0.5990	0.108*	
H17B	0.5809	−0.2753	0.5321	0.108*	
H17C	0.5554	−0.2501	0.5978	0.108*	
N21	0.36031 (17)	0.3352 (4)	0.6249 (3)	0.0517 (15)	
C22	0.3338 (2)	0.2403 (5)	0.5947 (3)	0.0533 (19)	
H22A	0.3109	0.2741	0.5666	0.064*	
H22B	0.3190	0.1944	0.6286	0.064*	
C23	0.3638 (2)	0.1617 (5)	0.5553 (3)	0.0516 (18)	
H23A	0.3464	0.0975	0.5367	0.062*	
H23B	0.3770	0.2063	0.5196	0.062*	
N24	0.39956 (17)	0.1136 (4)	0.5972 (3)	0.0491 (14)	
C25	0.4273 (2)	0.2089 (5)	0.6228 (4)	0.0578 (19)	
H25A	0.4409	0.2518	0.5868	0.069*	
H25B	0.4511	0.1765	0.6498	0.069*	
C26	0.3984 (2)	0.2924 (5)	0.6634 (3)	0.0552 (19)	
H26A	0.3875	0.2515	0.7020	0.066*	
H26B	0.4165	0.3589	0.6777	0.066*	
C217	0.3525 (2)	0.4513 (5)	0.6176 (3)	0.0495 (18)	
O217	0.37517 (19)	0.5257 (4)	0.6404 (3)	0.0794 (18)	
C211	0.3121 (2)	0.4872 (5)	0.5767 (3)	0.0415 (15)	
C212	0.3152 (2)	0.5063 (6)	0.5109 (4)	0.0514 (19)	
H212	0.3425	0.4934	0.4898	0.062*	
C213	0.2792 (2)	0.5440 (7)	0.4754 (4)	0.067 (2)	
H213	0.2821	0.5592	0.4308	0.081*	
C214	0.2386 (2)	0.5591 (6)	0.5065 (4)	0.0553 (19)	
Cl24	0.19252 (7)	0.6062 (2)	0.46025 (13)	0.1053 (8)	
C215	0.2338 (2)	0.5356 (6)	0.5718 (4)	0.058 (2)	
H215	0.2058	0.5426	0.5917	0.069*	
C216	0.2704 (2)	0.5016 (5)	0.6078 (4)	0.0474 (18)	
H216	0.2676	0.4880	0.6526	0.057*	
C241	0.4218 (2)	0.0166 (5)	0.5688 (4)	0.0412 (18)	
C242	0.3997 (2)	−0.0895 (5)	0.5609 (4)	0.0537 (19)	
C243	0.4200 (3)	−0.1866 (6)	0.5307 (4)	0.066 (2)	
H243	0.4040	−0.2563	0.5247	0.079*	
C244	0.4635 (3)	−0.1785 (7)	0.5101 (4)	0.085 (3)	
H244	0.4772	−0.2433	0.4905	0.102*	
C245	0.4873 (2)	−0.0742 (7)	0.5184 (4)	0.078 (2)	
H245	0.5172	−0.0691	0.5054	0.093*	
C246	0.4661 (3)	0.0219 (6)	0.5462 (4)	0.065 (2)	
H246	0.4818	0.0926	0.5501	0.078*	
O242	0.35673 (15)	−0.0912 (3)	0.5854 (2)	0.0653 (14)	
C247	0.3316 (3)	−0.1973 (6)	0.5820 (5)	0.086 (3)	
H27A	0.3477	−0.2595	0.6037	0.129*	
H27B	0.3269	−0.2183	0.5369	0.129*	
H27C	0.3030	−0.1861	0.6031	0.129*	
N31	0.51402 (18)	0.7839 (4)	0.7264 (3)	0.0543 (15)	
C32	0.5403 (2)	0.6882 (5)	0.7510 (4)	0.0553 (19)	
H32A	0.5660	0.7190	0.7748	0.066*	
H32B	0.5514	0.6411	0.7147	0.066*	
C33	0.51241 (19)	0.6115 (5)	0.7960 (3)	0.0499 (18)	
H33A	0.5301	0.5444	0.8104	0.060*	
H33B	0.5038	0.6566	0.8345	0.060*	
N34	0.47232 (17)	0.5698 (4)	0.7625 (3)	0.0479 (14)	
C35	0.4458 (2)	0.6681 (5)	0.7395 (4)	0.0536 (18)	
H35A	0.4358	0.7147	0.7767	0.064*	
H35B	0.4194	0.6388	0.7168	0.064*	
C36	0.4730 (2)	0.7450 (5)	0.6937 (4)	0.061 (2)	
H36A	0.4805	0.7008	0.6545	0.073*	
H36B	0.4553	0.8132	0.6808	0.073*	
C317	0.5217 (2)	0.8983 (5)	0.7386 (3)	0.0463 (16)	
O317	0.49520 (18)	0.9776 (4)	0.7174 (3)	0.0718 (16)	
C311	0.5620 (2)	0.9302 (5)	0.7773 (3)	0.0469 (16)	
C312	0.5590 (2)	0.9389 (7)	0.8444 (4)	0.068 (2)	
H312	0.5314	0.9256	0.8646	0.082*	
C313	0.5957 (2)	0.9669 (6)	0.8828 (4)	0.064 (2)	
H313	0.5929	0.9738	0.9280	0.077*	
C314	0.6350 (2)	0.9836 (5)	0.8536 (4)	0.0483 (18)	
Cl34	0.68265 (6)	1.01261 (19)	0.90174 (13)	0.0937 (8)	
C315	0.6405 (2)	0.9784 (5)	0.7873 (4)	0.058 (2)	
H315	0.6683	0.9928	0.7681	0.069*	
C316	0.6028 (2)	0.9503 (6)	0.7492 (4)	0.0535 (19)	
H316	0.6057	0.9454	0.7039	0.064*	
C341	0.4485 (2)	0.4783 (6)	0.7950 (4)	0.0440 (18)	
C342	0.4697 (2)	0.3656 (6)	0.8040 (3)	0.0525 (18)	
C343	0.4473 (3)	0.2761 (5)	0.8370 (4)	0.063 (2)	
H343	0.4607	0.2024	0.8421	0.076*	
C344	0.4048 (3)	0.2968 (8)	0.8623 (4)	0.079 (3)	
H344	0.3902	0.2374	0.8854	0.094*	
C345	0.3841 (3)	0.4037 (8)	0.8537 (4)	0.076 (2)	
H345	0.3552	0.4159	0.8697	0.091*	
C346	0.4063 (2)	0.4928 (6)	0.8216 (4)	0.056 (2)	
H346	0.3922	0.5658	0.8175	0.068*	
O342	0.51047 (15)	0.3541 (3)	0.7760 (2)	0.0627 (13)	
C347	0.5334 (2)	0.2433 (5)	0.7824 (4)	0.073 (2)	
H37A	0.5616	0.2470	0.7598	0.109*	
H37B	0.5153	0.1815	0.7639	0.109*	
H37C	0.5386	0.2271	0.8280	0.109*	
N41	0.76184 (16)	0.8298 (4)	0.7384 (3)	0.0496 (14)	
C42	0.7884 (2)	0.7361 (5)	0.7680 (4)	0.0513 (18)	
H42A	0.8099	0.7701	0.7984	0.062*	
H42B	0.8050	0.6944	0.7343	0.062*	
C43	0.7579 (2)	0.6502 (5)	0.8042 (3)	0.0493 (17)	
H43A	0.7758	0.5852	0.8211	0.059*	
H43B	0.7442	0.6902	0.8412	0.059*	
N44	0.72332 (15)	0.6044 (4)	0.7622 (3)	0.0421 (12)	
C45	0.69544 (19)	0.6998 (5)	0.7359 (3)	0.0505 (17)	
H45A	0.6804	0.7405	0.7716	0.061*	
H45B	0.6727	0.6672	0.7072	0.061*	
C46	0.7242 (2)	0.7854 (5)	0.6985 (3)	0.0533 (17)	
H46A	0.7360	0.7468	0.6597	0.064*	
H46B	0.7059	0.8516	0.6843	0.064*	
C417	0.7706 (2)	0.9443 (6)	0.7429 (3)	0.0517 (17)	
O417	0.74624 (19)	1.0202 (4)	0.7176 (3)	0.0764 (18)	
C411	0.8114 (2)	0.9795 (5)	0.7815 (3)	0.0437 (16)	
C412	0.8091 (3)	1.0004 (6)	0.8471 (4)	0.065 (2)	
H412	0.7817	0.9911	0.8686	0.078*	
C413	0.8463 (3)	1.0350 (7)	0.8826 (4)	0.074 (2)	
H413	0.8445	1.0484	0.9275	0.088*	
C414	0.8860 (2)	1.0489 (7)	0.8495 (4)	0.061 (2)	
Cl44	0.93398 (6)	1.0942 (2)	0.89333 (12)	0.1122 (9)	
C415	0.8895 (2)	1.0318 (6)	0.7838 (4)	0.059 (2)	
H415	0.9167	1.0436	0.7623	0.071*	
C416	0.8525 (3)	0.9972 (5)	0.7503 (4)	0.053 (2)	
H416	0.8546	0.9849	0.7053	0.064*	
C441	0.7014 (2)	0.5048 (5)	0.7872 (4)	0.0427 (18)	
C442	0.7250 (2)	0.3989 (5)	0.7907 (3)	0.0452 (16)	
C443	0.7041 (2)	0.3003 (6)	0.8165 (3)	0.0619 (19)	
H443	0.7202	0.2303	0.8206	0.074*	
C444	0.6596 (3)	0.3034 (6)	0.8362 (4)	0.0650 (19)	
H444	0.6457	0.2360	0.8527	0.078*	
C445	0.6364 (2)	0.4066 (7)	0.8312 (4)	0.069 (2)	
H445	0.6066	0.4094	0.8447	0.082*	
C446	0.6563 (2)	0.5062 (6)	0.8068 (4)	0.057 (2)	
H446	0.6397	0.5755	0.8031	0.069*	
O442	0.76813 (15)	0.4015 (3)	0.7700 (3)	0.0609 (13)	
C447	0.7927 (2)	0.2936 (6)	0.7641 (5)	0.083 (3)	
H47A	0.8213	0.3092	0.7441	0.124*	
H47B	0.7761	0.2391	0.7374	0.124*	
H57C	0.7973	0.2600	0.8068	0.124*	

### 1-(4-Chlorobenzoyl)-4-(2-methoxyphenyl)piperazine (II) Atomic displacement parameters (Å^2^)

**Table:**

	*U*^11^	*U*^22^	*U*^33^	*U*^12^	*U*^13^	*U*^23^
N11	0.062 (3)	0.037 (3)	0.067 (4)	−0.003 (3)	−0.022 (3)	−0.003 (3)
C12	0.048 (4)	0.045 (4)	0.076 (5)	−0.003 (3)	−0.009 (4)	−0.012 (4)
C13	0.050 (4)	0.054 (4)	0.062 (5)	−0.007 (3)	−0.016 (4)	−0.001 (4)
N14	0.041 (3)	0.045 (3)	0.051 (4)	−0.007 (2)	−0.015 (3)	0.007 (3)
C15	0.053 (4)	0.057 (4)	0.067 (5)	−0.003 (3)	−0.023 (4)	0.017 (4)
C16	0.069 (5)	0.048 (4)	0.058 (5)	−0.005 (3)	−0.030 (4)	0.005 (4)
C117	0.056 (4)	0.053 (4)	0.049 (4)	0.005 (3)	−0.008 (4)	−0.003 (4)
O117	0.080 (4)	0.047 (3)	0.105 (5)	−0.020 (3)	−0.047 (4)	−0.009 (3)
C111	0.051 (4)	0.055 (4)	0.038 (4)	0.006 (3)	−0.008 (3)	−0.009 (3)
C112	0.045 (4)	0.119 (7)	0.042 (5)	0.012 (4)	0.003 (4)	−0.012 (5)
C113	0.065 (6)	0.102 (6)	0.045 (5)	0.019 (5)	−0.007 (5)	0.000 (5)
C114	0.041 (4)	0.071 (5)	0.047 (5)	0.000 (3)	−0.005 (4)	0.001 (4)
Cl14	0.0625 (12)	0.1277 (18)	0.0809 (16)	0.0193 (11)	−0.0213 (12)	−0.0112 (14)
C115	0.039 (4)	0.050 (4)	0.055 (6)	0.006 (3)	0.001 (4)	−0.006 (4)
C116	0.064 (5)	0.057 (4)	0.035 (4)	−0.002 (4)	0.012 (4)	−0.002 (4)
C141	0.048 (4)	0.045 (4)	0.036 (4)	0.002 (3)	−0.005 (4)	0.008 (3)
C142	0.039 (4)	0.061 (4)	0.064 (5)	0.010 (3)	0.006 (4)	0.010 (4)
C143	0.073 (5)	0.061 (4)	0.068 (5)	0.006 (4)	−0.004 (4)	−0.009 (4)
C144	0.074 (5)	0.080 (6)	0.077 (6)	0.027 (5)	0.020 (5)	0.007 (5)
C145	0.046 (4)	0.106 (6)	0.061 (5)	0.013 (5)	0.012 (4)	0.026 (5)
C146	0.041 (4)	0.072 (5)	0.049 (5)	0.000 (3)	0.007 (4)	0.018 (4)
O142	0.054 (3)	0.047 (2)	0.084 (4)	−0.008 (2)	0.011 (3)	0.001 (3)
C147	0.064 (5)	0.056 (4)	0.095 (7)	−0.014 (4)	−0.002 (5)	−0.001 (4)
N21	0.057 (3)	0.038 (3)	0.060 (4)	−0.005 (3)	−0.021 (3)	−0.010 (3)
C22	0.056 (4)	0.039 (3)	0.065 (5)	−0.001 (3)	−0.018 (4)	0.003 (3)
C23	0.058 (4)	0.040 (3)	0.057 (5)	0.008 (3)	−0.024 (4)	−0.009 (3)
N24	0.055 (3)	0.040 (3)	0.053 (4)	0.002 (2)	−0.007 (3)	−0.006 (3)
C25	0.067 (5)	0.045 (4)	0.061 (5)	−0.003 (3)	−0.022 (4)	−0.001 (4)
C26	0.066 (4)	0.043 (4)	0.056 (5)	−0.002 (3)	−0.030 (4)	−0.005 (3)
C217	0.066 (5)	0.035 (4)	0.047 (4)	−0.003 (3)	−0.001 (4)	−0.011 (3)
O217	0.088 (4)	0.043 (3)	0.107 (5)	0.005 (3)	−0.046 (4)	−0.016 (3)
C211	0.048 (4)	0.038 (3)	0.039 (4)	−0.002 (3)	−0.011 (3)	−0.006 (3)
C212	0.041 (4)	0.074 (5)	0.039 (5)	0.000 (3)	0.010 (4)	0.003 (4)
C213	0.058 (5)	0.098 (6)	0.046 (5)	−0.010 (4)	−0.004 (4)	0.007 (5)
C214	0.055 (5)	0.056 (4)	0.054 (5)	0.003 (3)	−0.011 (4)	0.003 (4)
Cl24	0.0828 (14)	0.138 (2)	0.0947 (17)	0.0210 (14)	−0.0322 (13)	0.0055 (17)
C215	0.049 (5)	0.067 (4)	0.057 (6)	0.004 (3)	0.002 (4)	−0.004 (4)
C216	0.052 (4)	0.047 (4)	0.043 (5)	0.005 (3)	0.004 (4)	0.000 (3)
C241	0.046 (4)	0.040 (3)	0.038 (4)	0.004 (3)	−0.007 (3)	0.008 (3)
C242	0.058 (5)	0.044 (4)	0.060 (5)	−0.001 (3)	−0.009 (4)	0.003 (4)
C243	0.070 (5)	0.047 (4)	0.080 (6)	0.010 (4)	−0.010 (5)	−0.011 (4)
C244	0.097 (7)	0.064 (5)	0.093 (7)	0.037 (5)	−0.005 (6)	−0.006 (5)
C245	0.044 (4)	0.092 (6)	0.097 (7)	0.007 (4)	0.000 (4)	−0.004 (5)
C246	0.062 (5)	0.058 (4)	0.075 (6)	0.010 (4)	−0.009 (5)	−0.007 (4)
O242	0.064 (3)	0.048 (3)	0.084 (4)	−0.015 (2)	0.016 (3)	0.006 (3)
C247	0.086 (6)	0.051 (4)	0.122 (8)	−0.012 (4)	−0.005 (5)	0.003 (5)
N31	0.057 (3)	0.038 (3)	0.067 (4)	−0.004 (3)	−0.018 (3)	0.000 (3)
C32	0.059 (4)	0.041 (4)	0.066 (5)	0.005 (3)	−0.010 (4)	0.006 (4)
C33	0.047 (4)	0.036 (3)	0.066 (5)	−0.002 (3)	−0.016 (4)	−0.002 (3)
N34	0.051 (3)	0.042 (3)	0.051 (4)	0.006 (3)	−0.009 (3)	0.001 (3)
C35	0.051 (4)	0.047 (4)	0.063 (5)	0.007 (3)	−0.019 (4)	−0.013 (4)
C36	0.074 (5)	0.045 (4)	0.064 (5)	0.006 (4)	−0.018 (4)	0.001 (4)
C317	0.052 (4)	0.043 (4)	0.043 (4)	0.011 (3)	−0.006 (3)	0.000 (3)
O317	0.079 (4)	0.045 (3)	0.091 (4)	−0.004 (3)	−0.037 (3)	−0.003 (3)
C311	0.061 (5)	0.037 (3)	0.042 (4)	0.004 (3)	0.007 (4)	−0.002 (3)
C312	0.048 (5)	0.099 (6)	0.057 (5)	−0.015 (4)	0.009 (4)	−0.004 (5)
C313	0.047 (4)	0.110 (6)	0.036 (5)	−0.012 (4)	0.005 (4)	0.008 (5)
C314	0.048 (4)	0.049 (4)	0.048 (5)	0.000 (3)	−0.012 (4)	0.012 (3)
Cl34	0.0615 (12)	0.1306 (19)	0.0890 (18)	−0.0178 (11)	−0.0287 (11)	0.0360 (15)
C315	0.053 (5)	0.051 (4)	0.069 (6)	0.002 (3)	0.017 (4)	0.013 (4)
C316	0.061 (5)	0.057 (4)	0.042 (5)	−0.010 (4)	0.006 (4)	0.002 (4)
C341	0.046 (4)	0.051 (4)	0.035 (4)	−0.010 (3)	−0.009 (3)	−0.015 (3)
C342	0.068 (5)	0.048 (4)	0.042 (4)	−0.002 (4)	−0.005 (4)	−0.007 (3)
C343	0.081 (5)	0.038 (4)	0.070 (5)	−0.011 (4)	0.006 (5)	0.003 (4)
C344	0.063 (5)	0.102 (7)	0.072 (6)	−0.037 (5)	−0.004 (4)	0.001 (5)
C345	0.063 (5)	0.090 (6)	0.074 (6)	−0.010 (5)	−0.001 (5)	−0.020 (5)
C346	0.035 (4)	0.062 (5)	0.072 (6)	−0.005 (3)	−0.003 (4)	−0.015 (4)
O342	0.066 (3)	0.049 (3)	0.073 (3)	0.011 (2)	0.007 (3)	−0.001 (3)
C347	0.069 (5)	0.046 (4)	0.104 (7)	0.014 (4)	−0.011 (4)	−0.012 (4)
N41	0.049 (3)	0.044 (3)	0.057 (4)	−0.001 (2)	−0.020 (3)	0.005 (3)
C42	0.051 (4)	0.042 (3)	0.061 (5)	0.001 (3)	−0.017 (4)	0.018 (3)
C43	0.058 (4)	0.041 (3)	0.050 (4)	0.003 (3)	−0.006 (4)	0.009 (3)
N44	0.041 (3)	0.034 (3)	0.051 (3)	−0.004 (2)	−0.011 (3)	0.006 (3)
C45	0.048 (4)	0.042 (3)	0.061 (5)	0.002 (3)	−0.013 (3)	0.003 (3)
C46	0.062 (4)	0.042 (3)	0.055 (4)	−0.004 (3)	−0.016 (4)	0.008 (3)
C417	0.052 (4)	0.045 (4)	0.058 (5)	−0.003 (3)	−0.006 (4)	0.002 (4)
O417	0.071 (3)	0.047 (3)	0.112 (5)	0.008 (3)	−0.041 (4)	0.030 (3)
C411	0.045 (4)	0.035 (3)	0.051 (5)	0.006 (3)	−0.001 (4)	0.001 (3)
C412	0.045 (5)	0.100 (6)	0.049 (5)	−0.007 (4)	0.002 (4)	−0.002 (4)
C413	0.056 (5)	0.131 (7)	0.034 (5)	−0.016 (4)	0.002 (4)	0.010 (5)
C414	0.047 (5)	0.086 (5)	0.050 (5)	−0.008 (4)	−0.011 (4)	−0.001 (4)
Cl44	0.0610 (13)	0.205 (3)	0.0701 (15)	−0.0411 (14)	−0.0115 (11)	−0.0034 (18)
C415	0.049 (5)	0.068 (4)	0.060 (6)	−0.016 (4)	0.017 (4)	−0.002 (4)
C416	0.068 (5)	0.058 (4)	0.034 (4)	−0.008 (4)	0.009 (4)	−0.007 (3)
C441	0.041 (4)	0.042 (4)	0.046 (5)	−0.006 (3)	−0.002 (4)	−0.001 (3)
C442	0.049 (4)	0.037 (3)	0.050 (4)	−0.002 (3)	−0.005 (3)	−0.004 (3)
C443	0.084 (5)	0.046 (4)	0.056 (5)	−0.005 (4)	−0.005 (4)	0.006 (4)
C444	0.073 (5)	0.064 (5)	0.058 (5)	−0.020 (4)	−0.001 (4)	0.003 (4)
C445	0.052 (4)	0.078 (5)	0.076 (6)	−0.015 (4)	0.015 (4)	−0.013 (4)
C446	0.059 (5)	0.046 (4)	0.066 (6)	−0.005 (3)	−0.004 (5)	−0.001 (4)
O442	0.059 (3)	0.038 (2)	0.086 (4)	0.001 (2)	0.003 (3)	−0.002 (3)
C447	0.076 (5)	0.052 (4)	0.121 (7)	0.026 (4)	0.004 (5)	−0.017 (5)

### 1-(4-Chlorobenzoyl)-4-(2-methoxyphenyl)piperazine (II) Geometric parameters (Å, º)

**Table:**

N11—C117	1.344 (7)	N31—C317	1.337 (7)
N11—C16	1.459 (7)	N31—C32	1.428 (7)
N11—C12	1.485 (7)	N31—C36	1.461 (8)
C12—C13	1.476 (9)	C32—C33	1.513 (8)
C12—H12A	0.9700	C32—H32A	0.9700
C12—H12B	0.9700	C32—H32B	0.9700
C13—N14	1.471 (7)	C33—N34	1.455 (7)
C13—H13A	0.9700	C33—H33A	0.9700
C13—H13B	0.9700	C33—H33B	0.9700
N14—C141	1.412 (8)	N34—C341	1.420 (8)
N14—C15	1.465 (7)	N34—C35	1.442 (7)
C15—C16	1.499 (9)	C35—C36	1.512 (9)
C15—H15A	0.9700	C35—H35A	0.9700
C15—H15B	0.9700	C35—H35B	0.9700
C16—H16A	0.9700	C36—H36A	0.9700
C16—H16B	0.9700	C36—H36B	0.9700
C117—O117	1.191 (7)	C317—O317	1.270 (7)
C117—C111	1.503 (8)	C317—C311	1.481 (9)
C111—C116	1.371 (8)	C311—C316	1.363 (8)
C111—C112	1.404 (9)	C311—C312	1.377 (9)
C112—C113	1.355 (10)	C312—C313	1.380 (10)
C112—H112	0.9300	C312—H312	0.9300
C113—C114	1.389 (10)	C313—C314	1.328 (9)
C113—H113	0.9300	C313—H313	0.9300
C114—C115	1.356 (10)	C314—C315	1.363 (10)
C114—Cl14	1.726 (7)	C314—Cl34	1.756 (7)
C115—C116	1.382 (9)	C315—C316	1.402 (9)
C115—H115	0.9300	C315—H315	0.9300
C116—H116	0.9300	C316—H316	0.9300
C141—C146	1.360 (9)	C341—C346	1.379 (9)
C141—C142	1.382 (9)	C341—C342	1.436 (9)
C142—O142	1.375 (7)	C342—O342	1.347 (8)
C142—C143	1.390 (9)	C342—C343	1.388 (9)
C143—C144	1.388 (9)	C343—C344	1.385 (9)
C143—H143	0.9300	C343—H343	0.9300
C144—C145	1.356 (10)	C344—C345	1.369 (10)
C144—H144	0.9300	C344—H344	0.9300
C145—C146	1.412 (10)	C345—C346	1.372 (10)
C145—H145	0.9300	C345—H345	0.9300
C146—H146	0.9300	C346—H346	0.9300
O142—C147	1.403 (7)	O342—C347	1.433 (7)
C147—H17A	0.9600	C347—H37A	0.9600
C147—H17B	0.9600	C347—H37B	0.9600
C147—H17C	0.9600	C347—H37C	0.9600
N21—C217	1.342 (7)	N41—C417	1.326 (7)
N21—C26	1.462 (7)	N41—C42	1.454 (7)
N21—C22	1.469 (7)	N41—C46	1.473 (7)
C22—C23	1.495 (8)	C42—C43	1.521 (8)
C22—H22A	0.9700	C42—H42A	0.9700
C22—H22B	0.9700	C42—H42B	0.9700
C23—N24	1.470 (7)	C43—N44	1.437 (7)
C23—H23A	0.9700	C43—H43A	0.9700
C23—H23B	0.9700	C43—H43B	0.9700
N24—C241	1.408 (8)	N44—C441	1.398 (7)
N24—C25	1.454 (7)	N44—C45	1.464 (7)
C25—C26	1.522 (9)	C45—C46	1.501 (8)
C25—H25A	0.9700	C45—H45A	0.9700
C25—H25B	0.9700	C45—H45B	0.9700
C26—H26A	0.9700	C46—H46A	0.9700
C26—H26B	0.9700	C46—H46B	0.9700
C217—O217	1.175 (7)	C417—O417	1.236 (7)
C217—C211	1.518 (9)	C417—C411	1.503 (8)
C211—C212	1.362 (9)	C411—C412	1.361 (9)
C211—C216	1.405 (8)	C411—C416	1.395 (9)
C212—C213	1.362 (9)	C412—C413	1.381 (10)
C212—H212	0.9300	C412—H412	0.9300
C213—C214	1.377 (9)	C413—C414	1.371 (10)
C213—H213	0.9300	C413—H413	0.9300
C214—C215	1.366 (10)	C414—C415	1.359 (10)
C214—Cl24	1.748 (7)	C414—Cl44	1.760 (7)
C215—C216	1.369 (9)	C415—C416	1.354 (9)
C215—H215	0.9300	C415—H415	0.9300
C216—H216	0.9300	C416—H416	0.9300
C241—C242	1.379 (8)	C441—C442	1.391 (8)
C241—C246	1.395 (10)	C441—C446	1.402 (9)
C242—O242	1.374 (8)	C442—O442	1.351 (7)
C242—C243	1.397 (9)	C442—C443	1.383 (8)
C243—C244	1.363 (9)	C443—C444	1.385 (8)
C243—H243	0.9300	C443—H443	0.9300
C244—C245	1.387 (10)	C444—C445	1.360 (9)
C244—H244	0.9300	C444—H444	0.9300
C245—C246	1.380 (10)	C445—C446	1.368 (9)
C245—H245	0.9300	C445—H445	0.9300
C246—H246	0.9300	C446—H446	0.9300
O242—C247	1.417 (7)	O442—C447	1.429 (7)
C247—H27A	0.9600	C447—H47A	0.9600
C247—H27B	0.9600	C447—H47B	0.9600
C247—H27C	0.9600	C447—H57C	0.9600
			
C117—N11—C16	121.2 (5)	C317—N31—C32	125.2 (6)
C117—N11—C12	125.7 (6)	C317—N31—C36	121.3 (5)
C16—N11—C12	112.4 (5)	C32—N31—C36	113.0 (5)
C13—C12—N11	110.4 (5)	N31—C32—C33	110.3 (5)
C13—C12—H12A	109.6	N31—C32—H32A	109.6
N11—C12—H12A	109.6	C33—C32—H32A	109.6
C13—C12—H12B	109.6	N31—C32—H32B	109.6
N11—C12—H12B	109.6	C33—C32—H32B	109.6
H12A—C12—H12B	108.1	H32A—C32—H32B	108.1
N14—C13—C12	112.5 (6)	N34—C33—C32	110.5 (5)
N14—C13—H13A	109.1	N34—C33—H33A	109.5
C12—C13—H13A	109.1	C32—C33—H33A	109.5
N14—C13—H13B	109.1	N34—C33—H33B	109.5
C12—C13—H13B	109.1	C32—C33—H33B	109.5
H13A—C13—H13B	107.8	H33A—C33—H33B	108.1
C141—N14—C15	116.9 (5)	C341—N34—C35	116.2 (5)
C141—N14—C13	114.7 (5)	C341—N34—C33	115.2 (5)
C15—N14—C13	109.3 (5)	C35—N34—C33	110.6 (4)
N14—C15—C16	110.7 (5)	N34—C35—C36	110.6 (5)
N14—C15—H15A	109.5	N34—C35—H35A	109.5
C16—C15—H15A	109.5	C36—C35—H35A	109.5
N14—C15—H15B	109.5	N34—C35—H35B	109.5
C16—C15—H15B	109.5	C36—C35—H35B	109.5
H15A—C15—H15B	108.1	H35A—C35—H35B	108.1
N11—C16—C15	111.1 (5)	N31—C36—C35	109.8 (6)
N11—C16—H16A	109.4	N31—C36—H36A	109.7
C15—C16—H16A	109.4	C35—C36—H36A	109.7
N11—C16—H16B	109.4	N31—C36—H36B	109.7
C15—C16—H16B	109.4	C35—C36—H36B	109.7
H16A—C16—H16B	108.0	H36A—C36—H36B	108.2
O117—C117—N11	123.7 (6)	O317—C317—N31	121.0 (6)
O117—C117—C111	120.3 (6)	O317—C317—C311	120.7 (6)
N11—C117—C111	116.0 (6)	N31—C317—C311	118.3 (5)
C116—C111—C112	118.4 (6)	C316—C311—C312	117.6 (7)
C116—C111—C117	121.9 (6)	C316—C311—C317	122.7 (6)
C112—C111—C117	119.5 (6)	C312—C311—C317	119.7 (7)
C113—C112—C111	120.1 (7)	C311—C312—C313	122.0 (7)
C113—C112—H112	119.9	C311—C312—H312	119.0
C111—C112—H112	119.9	C313—C312—H312	119.0
C112—C113—C114	119.9 (8)	C314—C313—C312	118.4 (8)
C112—C113—H113	120.0	C314—C313—H313	120.8
C114—C113—H113	120.0	C312—C313—H313	120.8
C115—C114—C113	120.8 (8)	C313—C314—C315	123.0 (7)
C115—C114—Cl14	121.0 (6)	C313—C314—Cl34	119.2 (6)
C113—C114—Cl14	118.2 (7)	C315—C314—Cl34	117.8 (6)
C114—C115—C116	119.0 (7)	C314—C315—C316	117.6 (7)
C114—C115—H115	120.5	C314—C315—H315	121.2
C116—C115—H115	120.5	C316—C315—H315	121.2
C111—C116—C115	121.4 (7)	C311—C316—C315	121.2 (7)
C111—C116—H116	119.3	C311—C316—H316	119.4
C115—C116—H116	119.3	C315—C316—H316	119.4
C146—C141—C142	118.8 (6)	C346—C341—N34	123.5 (6)
C146—C141—N14	122.0 (6)	C346—C341—C342	117.2 (7)
C142—C141—N14	119.0 (6)	N34—C341—C342	119.2 (6)
O142—C142—C141	117.9 (6)	O342—C342—C343	124.7 (6)
O142—C142—C143	120.3 (6)	O342—C342—C341	115.4 (6)
C141—C142—C143	121.8 (7)	C343—C342—C341	119.9 (7)
C144—C143—C142	119.0 (7)	C344—C343—C342	119.7 (7)
C144—C143—H143	120.5	C344—C343—H343	120.1
C142—C143—H143	120.5	C342—C343—H343	120.1
C145—C144—C143	119.2 (7)	C345—C344—C343	120.9 (7)
C145—C144—H144	120.4	C345—C344—H344	119.5
C143—C144—H144	120.4	C343—C344—H344	119.5
C144—C145—C146	121.5 (7)	C344—C345—C346	119.6 (8)
C144—C145—H145	119.3	C344—C345—H345	120.2
C146—C145—H145	119.3	C346—C345—H345	120.2
C141—C146—C145	119.6 (7)	C345—C346—C341	122.6 (7)
C141—C146—H146	120.2	C345—C346—H346	118.7
C145—C146—H146	120.2	C341—C346—H346	118.7
C142—O142—C147	119.3 (5)	C342—O342—C347	118.3 (5)
O142—C147—H17A	109.5	O342—C347—H37A	109.5
O142—C147—H17B	109.5	O342—C347—H37B	109.5
H17A—C147—H17B	109.5	H37A—C347—H37B	109.5
O142—C147—H17C	109.5	O342—C347—H37C	109.5
H17A—C147—H17C	109.5	H37A—C347—H37C	109.5
H17B—C147—H17C	109.5	H37B—C347—H37C	109.5
C217—N21—C26	121.3 (5)	C417—N41—C42	125.3 (5)
C217—N21—C22	125.2 (6)	C417—N41—C46	121.4 (5)
C26—N21—C22	113.5 (5)	C42—N41—C46	113.3 (5)
N21—C22—C23	109.9 (5)	N41—C42—C43	110.1 (5)
N21—C22—H22A	109.7	N41—C42—H42A	109.7
C23—C22—H22A	109.7	C43—C42—H42A	109.7
N21—C22—H22B	109.7	N41—C42—H42B	109.7
C23—C22—H22B	109.7	C43—C42—H42B	109.7
H22A—C22—H22B	108.2	H42A—C42—H42B	108.2
N24—C23—C22	109.8 (5)	N44—C43—C42	111.7 (5)
N24—C23—H23A	109.7	N44—C43—H43A	109.3
C22—C23—H23A	109.7	C42—C43—H43A	109.3
N24—C23—H23B	109.7	N44—C43—H43B	109.3
C22—C23—H23B	109.7	C42—C43—H43B	109.3
H23A—C23—H23B	108.2	H43A—C43—H43B	107.9
C241—N24—C25	117.3 (5)	C441—N44—C43	114.1 (5)
C241—N24—C23	112.9 (5)	C441—N44—C45	117.7 (5)
C25—N24—C23	110.2 (5)	C43—N44—C45	111.0 (4)
N24—C25—C26	109.6 (5)	N44—C45—C46	109.8 (5)
N24—C25—H25A	109.7	N44—C45—H45A	109.7
C26—C25—H25A	109.7	C46—C45—H45A	109.7
N24—C25—H25B	109.7	N44—C45—H45B	109.7
C26—C25—H25B	109.7	C46—C45—H45B	109.7
H25A—C25—H25B	108.2	H45A—C45—H45B	108.2
N21—C26—C25	110.6 (5)	N41—C46—C45	111.9 (5)
N21—C26—H26A	109.5	N41—C46—H46A	109.2
C25—C26—H26A	109.5	C45—C46—H46A	109.2
N21—C26—H26B	109.5	N41—C46—H46B	109.2
C25—C26—H26B	109.5	C45—C46—H46B	109.2
H26A—C26—H26B	108.1	H46A—C46—H46B	107.9
O217—C217—N21	123.9 (7)	O417—C417—N41	122.4 (6)
O217—C217—C211	118.7 (6)	O417—C417—C411	120.6 (6)
N21—C217—C211	117.4 (6)	N41—C417—C411	117.0 (5)
C212—C211—C216	119.1 (6)	C412—C411—C416	118.0 (7)
C212—C211—C217	122.1 (6)	C412—C411—C417	121.3 (7)
C216—C211—C217	118.8 (6)	C416—C411—C417	120.6 (6)
C213—C212—C211	121.4 (7)	C411—C412—C413	121.6 (8)
C213—C212—H212	119.3	C411—C412—H412	119.2
C211—C212—H212	119.3	C413—C412—H412	119.2
C212—C213—C214	119.1 (7)	C414—C413—C412	117.9 (8)
C212—C213—H213	120.5	C414—C413—H413	121.1
C214—C213—H213	120.5	C412—C413—H413	121.1
C215—C214—C213	121.1 (7)	C415—C414—C413	122.4 (8)
C215—C214—Cl24	120.3 (6)	C415—C414—Cl44	118.8 (6)
C213—C214—Cl24	118.6 (6)	C413—C414—Cl44	118.8 (7)
C214—C215—C216	119.7 (7)	C416—C415—C414	118.5 (7)
C214—C215—H215	120.2	C416—C415—H415	120.7
C216—C215—H215	120.2	C414—C415—H415	120.7
C215—C216—C211	119.6 (7)	C415—C416—C411	121.7 (7)
C215—C216—H216	120.2	C415—C416—H416	119.2
C211—C216—H216	120.2	C411—C416—H416	119.2
C242—C241—C246	116.8 (7)	C442—C441—N44	118.6 (6)
C242—C241—N24	120.1 (7)	C442—C441—C446	118.6 (6)
C246—C241—N24	123.1 (6)	N44—C441—C446	122.8 (6)
O242—C242—C241	114.5 (6)	O442—C442—C443	124.4 (6)
O242—C242—C243	123.5 (6)	O442—C442—C441	116.4 (6)
C241—C242—C243	121.9 (7)	C443—C442—C441	119.2 (6)
C244—C243—C242	119.6 (7)	C442—C443—C444	121.4 (6)
C244—C243—H243	120.2	C442—C443—H443	119.3
C242—C243—H243	120.2	C444—C443—H443	119.3
C243—C244—C245	120.3 (7)	C445—C444—C443	119.0 (7)
C243—C244—H244	119.9	C445—C444—H444	120.5
C245—C244—H244	119.9	C443—C444—H444	120.5
C246—C245—C244	119.1 (8)	C444—C445—C446	121.0 (7)
C246—C245—H245	120.4	C444—C445—H445	119.5
C244—C245—H245	120.4	C446—C445—H445	119.5
C245—C246—C241	122.2 (7)	C445—C446—C441	120.7 (7)
C245—C246—H246	118.9	C445—C446—H446	119.7
C241—C246—H246	118.9	C441—C446—H446	119.7
C242—O242—C247	119.1 (5)	C442—O442—C447	119.7 (5)
O242—C247—H27A	109.5	O442—C447—H47A	109.5
O242—C247—H27B	109.5	O442—C447—H47B	109.5
H27A—C247—H27B	109.5	H47A—C447—H47B	109.5
O242—C247—H27C	109.5	O442—C447—H57C	109.5
H27A—C247—H27C	109.5	H47A—C447—H57C	109.5
H27B—C247—H27C	109.5	H47B—C447—H57C	109.5
			
C117—N11—C12—C13	118.3 (7)	C317—N31—C32—C33	116.0 (7)
C16—N11—C12—C13	−52.6 (8)	C36—N31—C32—C33	−55.4 (7)
N11—C12—C13—N14	55.1 (7)	N31—C32—C33—N34	56.0 (7)
C12—C13—N14—C141	167.9 (6)	C32—C33—N34—C341	167.6 (5)
C12—C13—N14—C15	−58.5 (7)	C32—C33—N34—C35	−58.2 (7)
C141—N14—C15—C16	−169.4 (5)	C341—N34—C35—C36	−167.7 (5)
C13—N14—C15—C16	58.1 (7)	C33—N34—C35—C36	58.5 (7)
C117—N11—C16—C15	−117.6 (7)	C317—N31—C36—C35	−116.4 (7)
C12—N11—C16—C15	53.7 (7)	C32—N31—C36—C35	55.3 (7)
N14—C15—C16—N11	−56.7 (7)	N34—C35—C36—N31	−55.9 (7)
C16—N11—C117—O117	−4.9 (11)	C32—N31—C317—O317	−176.4 (7)
C12—N11—C117—O117	−175.0 (7)	C36—N31—C317—O317	−5.7 (10)
C16—N11—C117—C111	174.2 (6)	C32—N31—C317—C311	3.8 (10)
C12—N11—C117—C111	4.1 (10)	C36—N31—C317—C311	174.5 (6)
O117—C117—C111—C116	−90.4 (9)	O317—C317—C311—C316	−91.3 (8)
N11—C117—C111—C116	90.5 (8)	N31—C317—C311—C316	88.5 (8)
O117—C117—C111—C112	84.4 (9)	O317—C317—C311—C312	89.6 (9)
N11—C117—C111—C112	−94.7 (8)	N31—C317—C311—C312	−90.6 (8)
C116—C111—C112—C113	−7.1 (11)	C316—C311—C312—C313	0.0 (11)
C117—C111—C112—C113	178.0 (7)	C317—C311—C312—C313	179.1 (6)
C111—C112—C113—C114	3.7 (12)	C311—C312—C313—C314	−1.2 (12)
C112—C113—C114—C115	1.7 (11)	C312—C313—C314—C315	2.3 (11)
C112—C113—C114—Cl14	180.0 (6)	C312—C313—C314—Cl34	−177.1 (6)
C113—C114—C115—C116	−3.6 (10)	C313—C314—C315—C316	−2.0 (10)
Cl14—C114—C115—C116	178.1 (5)	Cl34—C314—C315—C316	177.4 (5)
C112—C111—C116—C115	5.2 (10)	C312—C311—C316—C315	0.3 (10)
C117—C111—C116—C115	180.0 (6)	C317—C311—C316—C315	−178.8 (6)
C114—C115—C116—C111	0.1 (10)	C314—C315—C316—C311	0.7 (10)
C15—N14—C141—C146	−12.9 (9)	C35—N34—C341—C346	−19.2 (9)
C13—N14—C141—C146	117.1 (7)	C33—N34—C341—C346	112.5 (7)
C15—N14—C141—C142	162.7 (6)	C35—N34—C341—C342	163.9 (6)
C13—N14—C141—C142	−67.3 (8)	C33—N34—C341—C342	−64.4 (8)
C146—C141—C142—O142	178.2 (6)	C346—C341—C342—O342	178.5 (6)
N14—C141—C142—O142	2.4 (10)	N34—C341—C342—O342	−4.4 (9)
C146—C141—C142—C143	−3.2 (11)	C346—C341—C342—C343	1.4 (10)
N14—C141—C142—C143	−179.0 (6)	N34—C341—C342—C343	178.5 (6)
O142—C142—C143—C144	−178.5 (6)	O342—C342—C343—C344	−178.1 (7)
C141—C142—C143—C144	2.9 (11)	C341—C342—C343—C344	−1.3 (11)
C142—C143—C144—C145	−2.3 (11)	C342—C343—C344—C345	1.7 (12)
C143—C144—C145—C146	2.1 (12)	C343—C344—C345—C346	−2.1 (12)
C142—C141—C146—C145	2.9 (11)	C344—C345—C346—C341	2.3 (12)
N14—C141—C146—C145	178.5 (6)	N34—C341—C346—C345	−178.9 (7)
C144—C145—C146—C141	−2.4 (12)	C342—C341—C346—C345	−1.9 (11)
C141—C142—O142—C147	179.7 (6)	C343—C342—O342—C347	−2.4 (10)
C143—C142—O142—C147	1.1 (10)	C341—C342—O342—C347	−179.4 (6)
C217—N21—C22—C23	124.0 (7)	C417—N41—C42—C43	131.7 (6)
C26—N21—C22—C23	−53.9 (7)	C46—N41—C42—C43	−51.3 (7)
N21—C22—C23—N24	57.3 (7)	N41—C42—C43—N44	54.9 (7)
C22—C23—N24—C241	164.5 (5)	C42—C43—N44—C441	165.0 (5)
C22—C23—N24—C25	−62.2 (7)	C42—C43—N44—C45	−59.2 (7)
C241—N24—C25—C26	−168.6 (5)	C441—N44—C45—C46	−167.6 (6)
C23—N24—C25—C26	60.4 (7)	C43—N44—C45—C46	58.4 (7)
C217—N21—C26—C25	−125.3 (6)	C417—N41—C46—C45	−130.4 (6)
C22—N21—C26—C25	52.6 (7)	C42—N41—C46—C45	52.5 (7)
N24—C25—C26—N21	−55.1 (7)	N44—C45—C46—N41	−54.3 (7)
C26—N21—C217—O217	0.7 (11)	C42—N41—C417—O417	−178.7 (7)
C22—N21—C217—O217	−177.0 (7)	C46—N41—C417—O417	4.6 (10)
C26—N21—C217—C211	179.8 (6)	C42—N41—C417—C411	0.4 (10)
C22—N21—C217—C211	2.1 (10)	C46—N41—C417—C411	−176.4 (5)
O217—C217—C211—C212	88.5 (9)	O417—C417—C411—C412	89.6 (9)
N21—C217—C211—C212	−90.6 (8)	N41—C417—C411—C412	−89.4 (8)
O217—C217—C211—C216	−91.2 (8)	O417—C417—C411—C416	−87.2 (8)
N21—C217—C211—C216	89.6 (7)	N41—C417—C411—C416	93.7 (7)
C216—C211—C212—C213	2.9 (9)	C416—C411—C412—C413	−1.7 (10)
C217—C211—C212—C213	−176.9 (6)	C417—C411—C412—C413	−178.7 (6)
C211—C212—C213—C214	−2.2 (11)	C411—C412—C413—C414	0.6 (12)
C212—C213—C214—C215	−0.9 (11)	C412—C413—C414—C415	1.1 (13)
C212—C213—C214—Cl24	−179.5 (5)	C412—C413—C414—Cl44	179.5 (6)
C213—C214—C215—C216	3.0 (11)	C413—C414—C415—C416	−1.5 (12)
Cl24—C214—C215—C216	−178.4 (5)	Cl44—C414—C415—C416	−179.9 (5)
C214—C215—C216—C211	−2.2 (10)	C414—C415—C416—C411	0.3 (11)
C212—C211—C216—C215	−0.7 (9)	C412—C411—C416—C415	1.3 (10)
C217—C211—C216—C215	179.1 (6)	C417—C411—C416—C415	178.3 (6)
C25—N24—C241—C242	161.3 (7)	C43—N44—C441—C442	−69.9 (8)
C23—N24—C241—C242	−68.9 (8)	C45—N44—C441—C442	157.4 (6)
C25—N24—C241—C246	−19.9 (10)	C43—N44—C441—C446	112.5 (8)
C23—N24—C241—C246	109.8 (8)	C45—N44—C441—C446	−20.2 (10)
C246—C241—C242—O242	178.3 (6)	N44—C441—C442—O442	0.9 (10)
N24—C241—C242—O242	−2.8 (10)	C446—C441—C442—O442	178.7 (6)
C246—C241—C242—C243	−1.2 (11)	N44—C441—C442—C443	178.8 (6)
N24—C241—C242—C243	177.6 (7)	C446—C441—C442—C443	−3.5 (11)
O242—C242—C243—C244	−177.4 (7)	O442—C442—C443—C444	−179.5 (6)
C241—C242—C243—C244	2.2 (11)	C441—C442—C443—C444	2.9 (10)
C242—C243—C244—C245	−0.7 (12)	C442—C443—C444—C445	−1.3 (11)
C243—C244—C245—C246	−1.6 (13)	C443—C444—C445—C446	0.4 (11)
C244—C245—C246—C241	2.5 (13)	C444—C445—C446—C441	−1.1 (12)
C242—C241—C246—C245	−1.1 (12)	C442—C441—C446—C445	2.6 (12)
N24—C241—C246—C245	−180.0 (7)	N44—C441—C446—C445	−179.7 (7)
C241—C242—O242—C247	−177.7 (6)	C443—C442—O442—C447	10.3 (10)
C243—C242—O242—C247	1.8 (10)	C441—C442—O442—C447	−172.0 (7)

### 1-(4-Chlorobenzoyl)-4-(2-methoxyphenyl)piperazine (II) Hydrogen-bond geometry (Å, º)

**Table:**

*D*—H···*A*	*D*—H	H···*A*	*D*···*A*	*D*—H···*A*
C15—H15*B*···O417^i^	0.97	2.39	3.314 (9)	160
C35—H35*B*···O217	0.97	2.41	3.333 (9)	159
C115—H115···O217	0.93	2.60	3.522 (9)	174
C215—H215···O117^ii^	0.93	2.56	3.482 (8)	170
C315—H315···O417	0.93	2.56	3.486 (9)	177
C415—H415···O317^iii^	0.93	2.52	3.428 (8)	165
C213—H213···*Cg*2^iv^	0.93	2.71	3.604 (8)	161
C313—H313···*Cg*3^v^	0.93	2.79	3.633 (8)	151

Symmetry codes: (i) *x*, *y*−1, *z*; (ii) *x*−1/2, −*y*+1, *z*; (iii) *x*+1/2, −*y*+2, *z*; (iv) −*x*+1, −*y*+1, *z*−1/2; (v) −*x*+1, −*y*+1, *z*+1/2.

### 1-(4-Bromobenzoyl)-4-(2-methoxyphenyl)piperazine (III) Crystal data

**Table:**

C_18_H_19_BrN_2_O_2_	*D*_x_ = 1.427 Mg m^−^^3^
*M**_r_* = 375.26	Mo *K*α radiation, λ = 0.71073 Å
Orthorhombic, *P**c**a*2_1_	Cell parameters from 6870 reflections
*a* = 15.0779 (7) Å	θ = 2.7–27.8°
*b* = 11.2868 (6) Å	µ = 2.36 mm^−^^1^
*c* = 20.5297 (9) Å	*T* = 293 K
*V* = 3493.8 (3) Å^3^	Plate, yellow
*Z* = 8	0.50 × 0.48 × 0.24 mm
*F*(000) = 1536	

### 1-(4-Bromobenzoyl)-4-(2-methoxyphenyl)piperazine (III) Data collection

**Table:**

Oxford Diffraction Xcalibur CCD diffractometer	5910 independent reflections
Radiation source: Enhance (Mo) X-ray Source	3300 reflections with *I* > 2σ(*I*)
Graphite monochromator	*R*_int_ = 0.031
ω scans	θ_max_ = 25.5°, θ_min_ = 2.7°
Absorption correction: multi-scan (CrysalisRed; Oxford Diffraction, 2009)	*h* = −15→18
*T*_min_ = 0.294, *T*_max_ = 0.567	*k* = −6→13
13342 measured reflections	*l* = −23→24

### 1-(4-Bromobenzoyl)-4-(2-methoxyphenyl)piperazine (III) Refinement

**Table:**

Refinement on *F*^2^	Hydrogen site location: inferred from neighbouring sites
Least-squares matrix: full	H-atom parameters constrained
*R*[*F*^2^ > 2σ(*F*^2^)] = 0.051	*w* = 1/[σ^2^(*F*_o_^2^) + (0.0736*P*)^2^] where *P* = (*F*_o_^2^ + 2*F*_c_^2^)/3
*wR*(*F*^2^) = 0.130	(Δ/σ)_max_ < 0.001
*S* = 0.93	Δρ_max_ = 0.85 e Å^−^^3^
5910 reflections	Δρ_min_ = −0.49 e Å^−^^3^
445 parameters	Absolute structure: Flack *x* determined using 1109 quotients [(*I*^+^)-(*I*^-^)]/[(*I*^+^)+(*I*^-^)] (Parsons *et al.*, 2013)
21 restraints	Absolute structure parameter: 0.300 (6)
Primary atom site location: difference Fourier map	

### 1-(4-Bromobenzoyl)-4-(2-methoxyphenyl)piperazine (III) Special details

**Table:**

Geometry. All esds (except the esd in the dihedral angle between two l.s. planes) are estimated using the full covariance matrix. The cell esds are taken into account individually in the estimation of esds in distances, angles and torsion angles; correlations between esds in cell parameters are only used when they are defined by crystal symmetry. An approximate (isotropic) treatment of cell esds is used for estimating esds involving l.s. planes.

### 1-(4-Bromobenzoyl)-4-(2-methoxyphenyl)piperazine (III) Fractional atomic coordinates and isotropic or equivalent isotropic displacement parameters (Å^2^)

**Table:**

	*x*	*y*	*z*	*U*_iso_*/*U*_eq_	Occ. (<1)
N11	0.4662 (4)	0.8287 (6)	0.4090 (3)	0.0484 (17)	
C12	0.4124 (5)	0.7342 (7)	0.3813 (4)	0.051 (2)	
H12A	0.3689	0.7676	0.3517	0.061*	
H12B	0.3810	0.6931	0.4157	0.061*	
C13	0.4698 (5)	0.6491 (7)	0.3455 (4)	0.041 (2)	
H13A	0.4338	0.5840	0.3296	0.050*	
H13B	0.4958	0.6886	0.3081	0.050*	
N14	0.5405 (4)	0.6024 (5)	0.3866 (3)	0.0420 (16)	
C15	0.5982 (5)	0.6980 (7)	0.4117 (4)	0.048 (2)	
H15A	0.6279	0.7374	0.3758	0.058*	
H15B	0.6430	0.6652	0.4404	0.058*	
C16	0.5414 (5)	0.7856 (7)	0.4484 (4)	0.052 (2)	
H16A	0.5188	0.7482	0.4876	0.062*	
H16B	0.5776	0.8525	0.4615	0.062*	
C117	0.4531 (6)	0.9435 (7)	0.3982 (4)	0.043 (2)	
O117	0.5025 (5)	1.0208 (7)	0.4197 (4)	0.080 (2)	
C111	0.3746 (5)	0.9789 (7)	0.3586 (4)	0.038 (2)	
C112	0.2909 (6)	0.9914 (7)	0.3872 (4)	0.047 (2)	
H112	0.2853	0.9791	0.4318	0.057*	
C113	0.2174 (5)	1.0208 (7)	0.3525 (4)	0.046 (2)	
H113	0.1620	1.0245	0.3722	0.056*	
C114	0.2277 (5)	1.0449 (7)	0.2872 (4)	0.048 (2)	
Br14	0.12850 (7)	1.09205 (11)	0.23607 (7)	0.0933 (5)	
C115	0.3084 (6)	1.0379 (8)	0.2579 (4)	0.062 (3)	
H115	0.3147	1.0560	0.2139	0.074*	
C116	0.3820 (5)	1.0030 (7)	0.2949 (4)	0.047 (2)	
H116	0.4371	0.9966	0.2749	0.056*	
C141	0.5860 (6)	0.5030 (8)	0.3584 (4)	0.042 (2)	
C142	0.5363 (6)	0.4000 (7)	0.3524 (4)	0.051 (2)	
C143	0.5777 (8)	0.2964 (8)	0.3230 (5)	0.075 (3)	
H143	0.5466	0.2259	0.3182	0.090*	
C144	0.6638 (8)	0.3059 (11)	0.3028 (6)	0.083 (4)	
H144	0.6896	0.2421	0.2813	0.100*	
C145	0.7122 (8)	0.4031 (12)	0.3126 (5)	0.079 (3)	
H145	0.7723	0.4033	0.3025	0.094*	
C146	0.6722 (6)	0.5049 (9)	0.3382 (5)	0.058 (3)	
H146	0.7048	0.5746	0.3414	0.070*	
O142	0.4522 (4)	0.3980 (5)	0.3738 (3)	0.0636 (17)	
C147	0.4020 (7)	0.2964 (9)	0.3674 (7)	0.097 (4)	
H17A	0.3444	0.3091	0.3862	0.146*	
H17B	0.4308	0.2322	0.3895	0.146*	
H17C	0.3957	0.2772	0.3221	0.146*	
N21	0.7612 (4)	0.3202 (5)	0.5239 (3)	0.0449 (17)	
C22	0.8178 (5)	0.2274 (6)	0.5513 (4)	0.045 (2)	
H22A	0.8617	0.2626	0.5800	0.054*	
H22B	0.8489	0.1867	0.5165	0.054*	
C23	0.7622 (5)	0.1406 (7)	0.5887 (4)	0.0415 (19)	
H23A	0.7993	0.0768	0.6047	0.050*	
H23B	0.7356	0.1799	0.6260	0.050*	
N24	0.6921 (4)	0.0919 (5)	0.5470 (3)	0.0427 (16)	
C25	0.6329 (4)	0.1868 (7)	0.5259 (4)	0.041 (2)	
H25A	0.6071	0.2256	0.5636	0.050*	
H25B	0.5850	0.1541	0.4999	0.050*	
C26	0.6847 (5)	0.2759 (7)	0.4859 (4)	0.047 (2)	
H26A	0.7056	0.2388	0.4462	0.056*	
H26B	0.6465	0.3416	0.4741	0.056*	
C217	0.7746 (7)	0.4395 (11)	0.5319 (6)	0.042 (2)	0.939 (4)
O217	0.7231 (4)	0.5117 (7)	0.5111 (3)	0.054 (2)	0.939 (4)
C211	0.8552 (6)	0.4745 (15)	0.5681 (6)	0.042 (2)	0.939 (4)
C212	0.9355 (6)	0.4928 (7)	0.5353 (4)	0.038 (3)	0.939 (4)
H212	0.9375	0.4831	0.4904	0.045*	0.939 (4)
C213	1.0114 (5)	0.5248 (8)	0.5681 (4)	0.044 (2)	0.939 (4)
H213	1.0648	0.5340	0.5460	0.053*	0.939 (4)
C214	1.0065 (5)	0.5430 (9)	0.6344 (4)	0.046 (2)	0.939 (4)
Br24	1.11227 (7)	0.5820 (2)	0.67954 (7)	0.0915 (7)	0.939 (4)
C215	0.9303 (5)	0.5269 (12)	0.6674 (5)	0.059 (4)	0.939 (4)
H215	0.9289	0.5393	0.7122	0.071*	0.939 (4)
C216	0.8535 (7)	0.492 (3)	0.6350 (6)	0.057 (3)	0.939 (4)
H216	0.8012	0.4797	0.6582	0.068*	0.939 (4)
C317	0.784 (7)	0.435 (14)	0.527 (7)	0.042 (2)	0.061 (4)
O317	0.752 (7)	0.501 (12)	0.488 (5)	0.054 (2)	0.061 (4)
C311	0.864 (5)	0.47 (2)	0.565 (5)	0.042 (2)	0.061 (4)
C312	0.949 (6)	0.451 (14)	0.538 (4)	0.038 (3)	0.061 (4)
H312	0.9556	0.4474	0.4930	0.045*	0.061 (4)
C313	1.022 (5)	0.439 (11)	0.577 (3)	0.044 (2)	0.061 (4)
H313	1.0749	0.4073	0.5617	0.053*	0.061 (4)
C314	1.015 (2)	0.477 (12)	0.641 (3)	0.046 (2)	0.061 (4)
Br34	1.1196 (11)	0.486 (4)	0.6922 (10)	0.0915 (7)	0.061 (4)
C315	0.935 (2)	0.49 (2)	0.669 (4)	0.059 (4)	0.061 (4)
H315	0.9299	0.4948	0.7144	0.071*	0.061 (4)
C316	0.859 (4)	0.50 (4)	0.631 (7)	0.057 (3)	0.061 (4)
H316	0.8048	0.5180	0.6488	0.068*	0.061 (4)
C241	0.6512 (5)	−0.0094 (7)	0.5745 (4)	0.039 (2)	
C242	0.6990 (6)	−0.1170 (8)	0.5741 (4)	0.048 (2)	
C243	0.6623 (6)	−0.2178 (8)	0.6005 (4)	0.059 (2)	
H243	0.6946	−0.2880	0.6006	0.071*	
C244	0.5793 (8)	−0.2160 (9)	0.6263 (5)	0.069 (3)	
H244	0.5554	−0.2848	0.6441	0.083*	
C245	0.5290 (6)	−0.1107 (9)	0.6265 (5)	0.062 (2)	
H245	0.4729	−0.1080	0.6451	0.074*	
C246	0.5658 (6)	−0.0125 (8)	0.5982 (4)	0.055 (2)	
H246	0.5314	0.0556	0.5949	0.066*	
O242	0.7811 (4)	−0.1095 (5)	0.5471 (3)	0.0621 (17)	
C247	0.8328 (6)	−0.2165 (8)	0.5463 (6)	0.075 (3)	
H27A	0.8858	−0.2038	0.5214	0.112*	
H27B	0.7987	−0.2790	0.5268	0.112*	
H27C	0.8483	−0.2380	0.5901	0.112*	

### 1-(4-Bromobenzoyl)-4-(2-methoxyphenyl)piperazine (III) Atomic displacement parameters (Å^2^)

**Table:**

	*U*^11^	*U*^22^	*U*^33^	*U*^12^	*U*^13^	*U*^23^
N11	0.050 (4)	0.045 (4)	0.050 (4)	−0.002 (4)	−0.019 (3)	−0.003 (4)
C12	0.040 (4)	0.049 (6)	0.063 (6)	−0.006 (4)	−0.012 (4)	0.001 (5)
C13	0.042 (5)	0.035 (4)	0.048 (5)	0.004 (4)	−0.012 (4)	−0.003 (4)
N14	0.050 (4)	0.032 (4)	0.043 (4)	−0.002 (3)	−0.004 (4)	0.000 (3)
C15	0.040 (4)	0.053 (5)	0.052 (5)	−0.008 (4)	−0.006 (4)	0.003 (4)
C16	0.057 (5)	0.046 (5)	0.053 (5)	−0.001 (4)	−0.007 (4)	0.007 (4)
C117	0.045 (5)	0.035 (5)	0.049 (5)	−0.010 (4)	−0.002 (4)	−0.002 (4)
O117	0.080 (4)	0.064 (5)	0.097 (6)	−0.002 (4)	−0.035 (4)	−0.007 (4)
C111	0.037 (5)	0.030 (4)	0.049 (5)	−0.006 (4)	−0.003 (4)	−0.010 (4)
C112	0.058 (5)	0.047 (6)	0.037 (5)	0.010 (5)	0.014 (5)	−0.002 (4)
C113	0.044 (5)	0.044 (5)	0.050 (6)	0.009 (4)	0.010 (4)	0.002 (4)
C114	0.041 (5)	0.053 (5)	0.049 (5)	0.011 (4)	−0.005 (4)	−0.001 (5)
Br14	0.0623 (6)	0.1423 (11)	0.0755 (8)	0.0258 (6)	−0.0143 (6)	0.0010 (8)
C115	0.065 (6)	0.080 (7)	0.039 (5)	0.006 (5)	0.007 (5)	0.009 (5)
C116	0.027 (4)	0.067 (6)	0.046 (6)	0.007 (4)	0.002 (4)	0.000 (4)
C141	0.048 (5)	0.057 (6)	0.022 (4)	0.007 (5)	−0.007 (4)	0.005 (4)
C142	0.068 (6)	0.036 (5)	0.047 (5)	0.010 (5)	−0.001 (5)	−0.009 (4)
C143	0.091 (8)	0.058 (6)	0.076 (8)	0.007 (6)	−0.013 (6)	−0.004 (5)
C144	0.087 (9)	0.088 (9)	0.075 (8)	0.051 (7)	−0.006 (7)	−0.022 (7)
C145	0.067 (7)	0.121 (10)	0.048 (6)	0.044 (7)	0.004 (5)	0.014 (7)
C146	0.054 (6)	0.062 (7)	0.059 (6)	0.009 (5)	−0.004 (5)	0.010 (5)
O142	0.058 (4)	0.040 (4)	0.093 (5)	−0.002 (3)	0.003 (4)	0.001 (3)
C147	0.085 (7)	0.052 (7)	0.154 (12)	−0.006 (6)	0.010 (8)	−0.004 (7)
N21	0.053 (4)	0.038 (4)	0.045 (4)	0.000 (3)	−0.012 (3)	0.003 (3)
C22	0.038 (4)	0.037 (5)	0.059 (5)	0.002 (4)	−0.011 (4)	0.011 (4)
C23	0.040 (4)	0.038 (5)	0.047 (5)	0.001 (4)	−0.010 (4)	−0.001 (4)
N24	0.042 (4)	0.034 (4)	0.051 (4)	0.004 (3)	−0.011 (3)	0.003 (3)
C25	0.043 (4)	0.038 (4)	0.044 (5)	−0.001 (4)	−0.017 (4)	−0.009 (4)
C26	0.055 (5)	0.036 (5)	0.050 (5)	−0.003 (4)	−0.015 (4)	0.002 (4)
C217	0.044 (5)	0.045 (6)	0.037 (5)	−0.011 (5)	0.007 (4)	−0.001 (4)
O217	0.060 (4)	0.031 (4)	0.071 (5)	0.011 (4)	−0.023 (4)	0.004 (4)
C211	0.050 (5)	0.033 (5)	0.044 (5)	−0.007 (4)	−0.004 (5)	−0.001 (4)
C212	0.039 (5)	0.047 (7)	0.028 (4)	0.002 (5)	0.001 (4)	−0.001 (4)
C213	0.050 (5)	0.048 (6)	0.034 (5)	−0.008 (5)	0.009 (4)	−0.005 (5)
C214	0.042 (5)	0.056 (6)	0.039 (5)	−0.006 (4)	0.001 (4)	0.008 (5)
Br24	0.0498 (5)	0.168 (2)	0.0568 (7)	−0.0298 (8)	−0.0039 (6)	−0.0131 (11)
C215	0.040 (5)	0.110 (13)	0.029 (5)	−0.009 (5)	−0.002 (4)	0.001 (5)
C216	0.043 (5)	0.090 (9)	0.037 (5)	−0.008 (6)	0.014 (5)	0.002 (6)
C317	0.044 (5)	0.045 (6)	0.037 (5)	−0.011 (5)	0.007 (4)	−0.001 (4)
O317	0.060 (4)	0.031 (4)	0.071 (5)	0.011 (4)	−0.023 (4)	0.004 (4)
C311	0.050 (5)	0.033 (5)	0.044 (5)	−0.007 (4)	−0.004 (5)	−0.001 (4)
C312	0.039 (5)	0.047 (7)	0.028 (4)	0.002 (5)	0.001 (4)	−0.001 (4)
C313	0.050 (5)	0.048 (6)	0.034 (5)	−0.008 (5)	0.009 (4)	−0.005 (5)
C314	0.042 (5)	0.056 (6)	0.039 (5)	−0.006 (4)	0.001 (4)	0.008 (5)
Br34	0.0498 (5)	0.168 (2)	0.0568 (7)	−0.0298 (8)	−0.0039 (6)	−0.0131 (11)
C315	0.040 (5)	0.110 (13)	0.029 (5)	−0.009 (5)	−0.002 (4)	0.001 (5)
C316	0.043 (5)	0.090 (9)	0.037 (5)	−0.008 (6)	0.014 (5)	0.002 (6)
C241	0.038 (4)	0.043 (6)	0.036 (5)	0.000 (5)	−0.007 (4)	0.002 (4)
C242	0.055 (5)	0.045 (6)	0.044 (5)	−0.004 (5)	−0.004 (4)	−0.008 (4)
C243	0.075 (7)	0.044 (6)	0.058 (6)	−0.004 (5)	0.004 (5)	−0.007 (5)
C244	0.099 (8)	0.055 (7)	0.053 (6)	−0.032 (6)	−0.002 (6)	−0.004 (5)
C245	0.059 (5)	0.067 (7)	0.059 (6)	−0.023 (5)	−0.001 (5)	−0.010 (5)
C246	0.066 (6)	0.043 (6)	0.057 (6)	−0.002 (5)	−0.011 (5)	−0.008 (5)
O242	0.063 (4)	0.048 (4)	0.075 (4)	0.012 (3)	0.006 (4)	0.001 (3)
C247	0.073 (6)	0.055 (6)	0.096 (8)	0.019 (5)	−0.006 (6)	−0.022 (6)

### 1-(4-Bromobenzoyl)-4-(2-methoxyphenyl)piperazine (III) Geometric parameters (Å, º)

**Table:**

N11—C117	1.330 (9)	C23—N24	1.467 (9)
N11—C12	1.456 (9)	C23—H23A	0.9700
N11—C16	1.475 (9)	C23—H23B	0.9700
C12—C13	1.487 (10)	N24—C241	1.418 (9)
C12—H12A	0.9700	N24—C25	1.461 (9)
C12—H12B	0.9700	C25—C26	1.515 (10)
C13—N14	1.459 (9)	C25—H25A	0.9700
C13—H13A	0.9700	C25—H25B	0.9700
C13—H13B	0.9700	C26—H26A	0.9700
N14—C141	1.437 (10)	C26—H26B	0.9700
N14—C15	1.478 (9)	C217—O217	1.203 (10)
C15—C16	1.510 (11)	C217—C211	1.479 (11)
C15—H15A	0.9700	C211—C216	1.389 (12)
C15—H15B	0.9700	C211—C212	1.399 (12)
C16—H16A	0.9700	C212—C213	1.377 (11)
C16—H16B	0.9700	C212—H212	0.9300
C117—O117	1.229 (9)	C213—C214	1.378 (12)
C117—C111	1.491 (11)	C213—H213	0.9300
C111—C116	1.340 (12)	C214—C215	1.347 (11)
C111—C112	1.398 (10)	C214—Br24	1.896 (8)
C112—C113	1.358 (11)	C215—C216	1.393 (14)
C112—H112	0.9300	C215—H215	0.9300
C113—C114	1.376 (12)	C216—H216	0.9300
C113—H113	0.9300	C317—O317	1.203 (17)
C114—C115	1.360 (11)	C317—C311	1.480 (17)
C114—Br14	1.904 (8)	C311—C316	1.390 (17)
C115—C116	1.402 (11)	C311—C312	1.402 (17)
C115—H115	0.9300	C312—C313	1.378 (18)
C116—H116	0.9300	C312—H312	0.9300
C141—C146	1.365 (12)	C313—C314	1.380 (18)
C141—C142	1.389 (11)	C313—H313	0.9300
C142—O142	1.342 (10)	C314—C315	1.348 (17)
C142—C143	1.456 (12)	C314—Br34	1.896 (15)
C143—C144	1.367 (15)	C315—C316	1.39 (2)
C143—H143	0.9300	C315—H315	0.9300
C144—C145	1.332 (13)	C316—H316	0.9300
C144—H144	0.9300	C241—C246	1.377 (11)
C145—C146	1.399 (13)	C241—C242	1.412 (11)
C145—H145	0.9300	C242—O242	1.359 (9)
C146—H146	0.9300	C242—C243	1.377 (12)
O142—C147	1.381 (10)	C243—C244	1.359 (13)
C147—H17A	0.9600	C243—H243	0.9300
C147—H17B	0.9600	C244—C245	1.409 (12)
C147—H17C	0.9600	C244—H244	0.9300
N21—C317	1.35 (15)	C245—C246	1.368 (11)
N21—C217	1.371 (13)	C245—H245	0.9300
N21—C22	1.464 (9)	C246—H246	0.9300
N21—C26	1.479 (9)	O242—C247	1.437 (9)
C22—C23	1.502 (10)	C247—H27A	0.9600
C22—H22A	0.9700	C247—H27B	0.9600
C22—H22B	0.9700	C247—H27C	0.9600
			
C117—N11—C12	124.5 (7)	N24—C23—H23A	109.6
C117—N11—C16	121.8 (7)	C22—C23—H23A	109.6
C12—N11—C16	113.6 (6)	N24—C23—H23B	109.6
N11—C12—C13	110.0 (6)	C22—C23—H23B	109.6
N11—C12—H12A	109.7	H23A—C23—H23B	108.1
C13—C12—H12A	109.7	C241—N24—C25	116.3 (6)
N11—C12—H12B	109.7	C241—N24—C23	112.5 (6)
C13—C12—H12B	109.7	C25—N24—C23	109.8 (5)
H12A—C12—H12B	108.2	N24—C25—C26	109.4 (6)
N14—C13—C12	111.9 (7)	N24—C25—H25A	109.8
N14—C13—H13A	109.2	C26—C25—H25A	109.8
C12—C13—H13A	109.2	N24—C25—H25B	109.8
N14—C13—H13B	109.2	C26—C25—H25B	109.8
C12—C13—H13B	109.2	H25A—C25—H25B	108.3
H13A—C13—H13B	107.9	N21—C26—C25	110.0 (6)
C141—N14—C13	113.4 (6)	N21—C26—H26A	109.7
C141—N14—C15	115.4 (6)	C25—C26—H26A	109.7
C13—N14—C15	111.6 (6)	N21—C26—H26B	109.7
N14—C15—C16	108.5 (6)	C25—C26—H26B	109.7
N14—C15—H15A	110.0	H26A—C26—H26B	108.2
C16—C15—H15A	110.0	O217—C217—N21	121.9 (8)
N14—C15—H15B	110.0	O217—C217—C211	121.8 (8)
C16—C15—H15B	110.0	N21—C217—C211	116.4 (9)
H15A—C15—H15B	108.4	C216—C211—C212	118.1 (8)
N11—C16—C15	112.2 (7)	C216—C211—C217	121.3 (8)
N11—C16—H16A	109.2	C212—C211—C217	120.6 (8)
C15—C16—H16A	109.2	C213—C212—C211	121.5 (8)
N11—C16—H16B	109.2	C213—C212—H212	119.2
C15—C16—H16B	109.2	C211—C212—H212	119.2
H16A—C16—H16B	107.9	C212—C213—C214	118.5 (8)
O117—C117—N11	122.8 (8)	C212—C213—H213	120.7
O117—C117—C111	119.1 (8)	C214—C213—H213	120.7
N11—C117—C111	118.0 (7)	C215—C214—C213	121.5 (8)
C116—C111—C112	117.6 (8)	C215—C214—Br24	120.2 (7)
C116—C111—C117	121.3 (7)	C213—C214—Br24	118.2 (6)
C112—C111—C117	121.0 (8)	C214—C215—C216	120.5 (8)
C113—C112—C111	122.7 (8)	C214—C215—H215	119.7
C113—C112—H112	118.6	C216—C215—H215	119.7
C111—C112—H112	118.6	C211—C216—C215	119.8 (10)
C112—C113—C114	117.8 (8)	C211—C216—H216	120.1
C112—C113—H113	121.1	C215—C216—H216	120.1
C114—C113—H113	121.1	O317—C317—N21	117 (10)
C115—C114—C113	121.4 (8)	O317—C317—C311	122 (3)
C115—C114—Br14	118.4 (7)	N21—C317—C311	119 (8)
C113—C114—Br14	120.2 (6)	C316—C311—C312	117.8 (17)
C114—C115—C116	118.9 (8)	C316—C311—C317	121 (2)
C114—C115—H115	120.5	C312—C311—C317	120 (3)
C116—C115—H115	120.5	C313—C312—C311	121 (2)
C111—C116—C115	121.4 (8)	C313—C312—H312	119.5
C111—C116—H116	119.3	C311—C312—H312	119.5
C115—C116—H116	119.3	C312—C313—C314	118 (2)
C146—C141—C142	120.0 (9)	C312—C313—H313	121.2
C146—C141—N14	124.4 (9)	C314—C313—H313	121.2
C142—C141—N14	115.6 (8)	C315—C314—C313	121 (2)
O142—C142—C141	119.6 (7)	C315—C314—Br34	120.3 (19)
O142—C142—C143	121.8 (8)	C313—C314—Br34	118.3 (19)
C141—C142—C143	118.5 (9)	C314—C315—C316	120 (3)
C144—C143—C142	118.1 (10)	C314—C315—H315	120.1
C144—C143—H143	121.0	C316—C315—H315	120.1
C142—C143—H143	121.0	C311—C316—C315	120 (3)
C145—C144—C143	122.5 (10)	C311—C316—H316	120.2
C145—C144—H144	118.7	C315—C316—H316	120.2
C143—C144—H144	118.7	C246—C241—C242	117.2 (8)
C144—C145—C146	119.8 (10)	C246—C241—N24	124.6 (8)
C144—C145—H145	120.1	C242—C241—N24	118.0 (7)
C146—C145—H145	120.1	O242—C242—C243	125.3 (8)
C141—C146—C145	120.8 (10)	O242—C242—C241	114.5 (8)
C141—C146—H146	119.6	C243—C242—C241	120.2 (8)
C145—C146—H146	119.6	C244—C243—C242	120.7 (9)
C142—O142—C147	120.1 (7)	C244—C243—H243	119.6
O142—C147—H17A	109.5	C242—C243—H243	119.6
O142—C147—H17B	109.5	C243—C244—C245	120.6 (9)
H17A—C147—H17B	109.5	C243—C244—H244	119.7
O142—C147—H17C	109.5	C245—C244—H244	119.7
H17A—C147—H17C	109.5	C246—C245—C244	117.7 (9)
H17B—C147—H17C	109.5	C246—C245—H245	121.2
C317—N21—C22	121 (3)	C244—C245—H245	121.2
C217—N21—C22	124.8 (7)	C245—C246—C241	123.3 (9)
C317—N21—C26	124 (2)	C245—C246—H246	118.3
C217—N21—C26	120.7 (6)	C241—C246—H246	118.3
C22—N21—C26	114.5 (6)	C242—O242—C247	116.5 (7)
N21—C22—C23	109.7 (6)	O242—C247—H27A	109.5
N21—C22—H22A	109.7	O242—C247—H27B	109.5
C23—C22—H22A	109.7	H27A—C247—H27B	109.5
N21—C22—H22B	109.7	O242—C247—H27C	109.5
C23—C22—H22B	109.7	H27A—C247—H27C	109.5
H22A—C22—H22B	108.2	H27B—C247—H27C	109.5
N24—C23—C22	110.4 (6)		
			
C117—N11—C12—C13	124.5 (8)	C241—N24—C25—C26	−168.7 (7)
C16—N11—C12—C13	−52.3 (9)	C23—N24—C25—C26	62.0 (8)
N11—C12—C13—N14	55.3 (9)	C317—N21—C26—C25	−135 (10)
C12—C13—N14—C141	168.1 (7)	C217—N21—C26—C25	−127.2 (9)
C12—C13—N14—C15	−59.6 (8)	C22—N21—C26—C25	52.3 (9)
C141—N14—C15—C16	−171.5 (6)	N24—C25—C26—N21	−55.8 (8)
C13—N14—C15—C16	57.1 (9)	C22—N21—C217—O217	−176.0 (9)
C117—N11—C16—C15	−123.9 (8)	C26—N21—C217—O217	3.5 (15)
C12—N11—C16—C15	53.0 (9)	C22—N21—C217—C211	3.3 (13)
N14—C15—C16—N11	−53.5 (9)	C26—N21—C217—C211	−177.2 (7)
C12—N11—C117—O117	−176.2 (8)	O217—C217—C211—C216	87.7 (14)
C16—N11—C117—O117	0.4 (12)	N21—C217—C211—C216	−91.6 (16)
C12—N11—C117—C111	4.4 (11)	O217—C217—C211—C212	−91.5 (13)
C16—N11—C117—C111	−179.1 (7)	N21—C217—C211—C212	89.2 (15)
O117—C117—C111—C116	82.5 (11)	C216—C211—C212—C213	1.2 (15)
N11—C117—C111—C116	−98.0 (9)	C217—C211—C212—C213	−179.6 (9)
O117—C117—C111—C112	−95.6 (10)	C211—C212—C213—C214	−2.3 (15)
N11—C117—C111—C112	83.9 (10)	C212—C213—C214—C215	1.9 (16)
C116—C111—C112—C113	3.4 (12)	C212—C213—C214—Br24	178.0 (6)
C117—C111—C112—C113	−178.4 (8)	C213—C214—C215—C216	0 (2)
C111—C112—C113—C114	−3.4 (13)	Br24—C214—C215—C216	−176.4 (14)
C112—C113—C114—C115	1.0 (13)	C212—C211—C216—C215	0 (2)
C112—C113—C114—Br14	−177.8 (6)	C217—C211—C216—C215	−178.8 (17)
C113—C114—C115—C116	1.3 (13)	C214—C215—C216—C211	−1 (2)
Br14—C114—C115—C116	−179.9 (7)	C22—N21—C317—O317	157 (12)
C112—C111—C116—C115	−1.0 (13)	C26—N21—C317—O317	−15 (20)
C117—C111—C116—C115	−179.1 (8)	C22—N21—C317—C311	−6 (13)
C114—C115—C116—C111	−1.3 (14)	C26—N21—C317—C311	−178 (4)
C13—N14—C141—C146	112.4 (9)	O317—C317—C311—C312	−85 (17)
C15—N14—C141—C146	−18.1 (11)	N21—C317—C311—C312	77 (21)
C13—N14—C141—C142	−67.6 (9)	C317—C311—C312—C313	−156 (14)
C15—N14—C141—C142	161.9 (7)	C311—C312—C313—C314	−17 (18)
C146—C141—C142—O142	178.5 (8)	C312—C313—C314—C315	20 (17)
N14—C141—C142—O142	−1.5 (11)	C312—C313—C314—Br34	−170 (11)
C146—C141—C142—C143	−1.2 (12)	C25—N24—C241—C246	−18.1 (11)
N14—C141—C142—C143	178.8 (7)	C23—N24—C241—C246	109.8 (9)
O142—C142—C143—C144	−179.7 (9)	C25—N24—C241—C242	157.8 (7)
C141—C142—C143—C144	−0.1 (13)	C23—N24—C241—C242	−74.2 (9)
C142—C143—C144—C145	4.3 (16)	C246—C241—C242—O242	176.8 (7)
C143—C144—C145—C146	−7.1 (17)	N24—C241—C242—O242	0.6 (10)
C142—C141—C146—C145	−1.5 (13)	C246—C241—C242—C243	−4.0 (12)
N14—C141—C146—C145	178.5 (8)	N24—C241—C242—C243	179.8 (7)
C144—C145—C146—C141	5.6 (15)	O242—C242—C243—C244	−179.9 (8)
C141—C142—O142—C147	179.3 (9)	C241—C242—C243—C244	0.9 (13)
C143—C142—O142—C147	−1.0 (13)	C242—C243—C244—C245	0.2 (14)
C317—N21—C22—C23	135 (9)	C243—C244—C245—C246	1.8 (13)
C217—N21—C22—C23	127.4 (9)	C244—C245—C246—C241	−5.2 (13)
C26—N21—C22—C23	−52.1 (8)	C242—C241—C246—C245	6.2 (13)
N21—C22—C23—N24	56.0 (8)	N24—C241—C246—C245	−177.8 (8)
C22—C23—N24—C241	166.1 (6)	C243—C242—O242—C247	0.2 (13)
C22—C23—N24—C25	−62.6 (8)	C241—C242—O242—C247	179.3 (7)

### 1-(4-Bromobenzoyl)-4-(2-methoxyphenyl)piperazine (III) Hydrogen-bond geometry (Å, º)

**Table:**

*D*—H···*A*	*D*—H	H···*A*	*D*···*A*	*D*—H···*A*
C15—H15*B*···O217	0.97	2.56	3.483 (10)	159
C25—H25*B*···O117^i^	0.97	2.55	3.483 (11)	160
C213—H213···O217^ii^	0.93	2.54	3.425 (10)	158
C312—H312···N14^ii^	0.93	2.59	3.45 (9)	154
C115—H115···*Cg*4^iii^	0.93	2.65	3.549 (9)	162
C315—H315···*Cg*5^iv^	0.93	2.74	3.59 (10)	151

Symmetry codes: (i) *x*, *y*−1, *z*; (ii) *x*+1/2, −*y*+1, *z*; (iii) −*x*+1, −*y*+1, *z*−1/2; (iv) −*x*+3/2, *y*, *z*+1/2.

### 1-(4-Iodobenzoyl)-4-(2-methoxyphenyl)piperazine (IV) Crystal data

**Table:**

C_18_H_19_IN_2_O_2_	*F*(000) = 840
*M**_r_* = 422.25	*D*_x_ = 1.574 Mg m^−^^3^
Monoclinic, *P*2_1_/*c*	Mo *K*α radiation, λ = 0.71073 Å
*a* = 10.9626 (5) Å	Cell parameters from 3824 reflections
*b* = 11.3258 (6) Å	θ = 2.6–27.8°
*c* = 14.8234 (7) Å	µ = 1.81 mm^−^^1^
β = 104.520 (5)°	*T* = 296 K
*V* = 1781.69 (16) Å^3^	Block, yellow
*Z* = 4	0.42 × 0.40 × 0.28 mm

### 1-(4-Iodobenzoyl)-4-(2-methoxyphenyl)piperazine (IV) Data collection

**Table:**

Oxford Diffraction Xcalibur CCD diffractometer	3816 independent reflections
Radiation source: Enhance (Mo) X-ray Source	2690 reflections with *I* > 2σ(*I*)
Graphite monochromator	*R*_int_ = 0.015
ω scans	θ_max_ = 27.6°, θ_min_ = 2.6°
Absorption correction: multi-scan (CrysalisRed; Oxford Diffraction, 2009)	*h* = −13→14
*T*_min_ = 0.423, *T*_max_ = 0.603	*k* = −13→14
7512 measured reflections	*l* = −19→9

### 1-(4-Iodobenzoyl)-4-(2-methoxyphenyl)piperazine (IV) Refinement

**Table:**

Refinement on *F*^2^	Primary atom site location: difference Fourier map
Least-squares matrix: full	Hydrogen site location: inferred from neighbouring sites
*R*[*F*^2^ > 2σ(*F*^2^)] = 0.035	H-atom parameters constrained
*wR*(*F*^2^) = 0.092	*w* = 1/[σ^2^(*F*_o_^2^) + (0.0499*P*)^2^ + 0.0876*P*] where *P* = (*F*_o_^2^ + 2*F*_c_^2^)/3
*S* = 1.05	(Δ/σ)_max_ = 0.001
3816 reflections	Δρ_max_ = 0.43 e Å^−^^3^
208 parameters	Δρ_min_ = −0.91 e Å^−^^3^
0 restraints	

### 1-(4-Iodobenzoyl)-4-(2-methoxyphenyl)piperazine (IV) Special details

**Table:**

Geometry. All esds (except the esd in the dihedral angle between two l.s. planes) are estimated using the full covariance matrix. The cell esds are taken into account individually in the estimation of esds in distances, angles and torsion angles; correlations between esds in cell parameters are only used when they are defined by crystal symmetry. An approximate (isotropic) treatment of cell esds is used for estimating esds involving l.s. planes.

### 1-(4-Iodobenzoyl)-4-(2-methoxyphenyl)piperazine (IV) Fractional atomic coordinates and isotropic or equivalent isotropic displacement parameters (Å^2^)

**Table:**

	*x*	*y*	*z*	*U*_iso_*/*U*_eq_	
N1	0.3873 (2)	0.4258 (2)	0.37476 (16)	0.0455 (6)	
C2	0.4605 (3)	0.4724 (3)	0.3125 (2)	0.0498 (7)	
H2A	0.4840	0.4081	0.2770	0.060*	
H2B	0.5372	0.5085	0.3493	0.060*	
C3	0.3855 (3)	0.5627 (2)	0.2466 (2)	0.0450 (7)	
H3A	0.4373	0.5967	0.2090	0.054*	
H3B	0.3133	0.5252	0.2052	0.054*	
N4	0.3431 (2)	0.65533 (19)	0.29992 (16)	0.0417 (5)	
C5	0.2621 (3)	0.6056 (2)	0.3557 (2)	0.0455 (7)	
H5A	0.1898	0.5673	0.3151	0.055*	
H5B	0.2318	0.6683	0.3890	0.055*	
C6	0.3355 (3)	0.5175 (2)	0.4240 (2)	0.0484 (7)	
H6A	0.4035	0.5573	0.4680	0.058*	
H6B	0.2808	0.4817	0.4586	0.058*	
C17	0.3711 (3)	0.3096 (3)	0.3832 (2)	0.0479 (7)	
O17	0.4138 (3)	0.23600 (19)	0.3391 (2)	0.0806 (8)	
C11	0.2936 (3)	0.2704 (2)	0.4481 (2)	0.0432 (6)	
C12	0.1691 (3)	0.2393 (3)	0.4107 (2)	0.0545 (8)	
H12	0.1349	0.2448	0.3468	0.065*	
C13	0.0952 (3)	0.2003 (3)	0.4677 (2)	0.0558 (8)	
H13	0.0115	0.1802	0.4421	0.067*	
C14	0.1450 (3)	0.1910 (3)	0.5617 (2)	0.0444 (7)	
I14	0.02671 (2)	0.13596 (2)	0.64562 (2)	0.06954 (12)	
C15	0.2690 (3)	0.2190 (2)	0.6004 (2)	0.0452 (7)	
H15	0.3028	0.2120	0.6643	0.054*	
C16	0.3432 (3)	0.2578 (2)	0.5431 (2)	0.0456 (7)	
H16	0.4276	0.2756	0.5689	0.055*	
C41	0.2909 (2)	0.7580 (2)	0.2498 (2)	0.0428 (7)	
C42	0.2875 (3)	0.8622 (2)	0.3012 (2)	0.0469 (7)	
C43	0.2366 (3)	0.9643 (3)	0.2570 (3)	0.0602 (9)	
H43	0.2338	1.0324	0.2913	0.072*	
C44	0.1895 (3)	0.9651 (3)	0.1611 (3)	0.0685 (10)	
H44	0.1541	1.0339	0.1314	0.082*	
C45	0.1944 (3)	0.8660 (3)	0.1098 (3)	0.0667 (10)	
H45	0.1638	0.8679	0.0454	0.080*	
C46	0.2448 (3)	0.7628 (3)	0.1535 (2)	0.0548 (8)	
H46	0.2480	0.6957	0.1180	0.066*	
O42	0.3380 (2)	0.85339 (17)	0.39492 (16)	0.0572 (6)	
C47	0.3405 (3)	0.9558 (3)	0.4503 (3)	0.0708 (10)	
H47A	0.3783	0.9371	0.5144	0.106*	
H47B	0.3889	1.0162	0.4299	0.106*	
H47C	0.2561	0.9834	0.4441	0.106*	

### 1-(4-Iodobenzoyl)-4-(2-methoxyphenyl)piperazine (IV) Atomic displacement parameters (Å^2^)

**Table:**

	*U*^11^	*U*^22^	*U*^33^	*U*^12^	*U*^13^	*U*^23^
N1	0.0542 (13)	0.0294 (13)	0.0607 (15)	0.0040 (11)	0.0290 (12)	0.0009 (11)
C2	0.0506 (16)	0.0394 (17)	0.0663 (18)	0.0016 (13)	0.0277 (14)	−0.0025 (15)
C3	0.0513 (15)	0.0349 (16)	0.0567 (17)	−0.0043 (13)	0.0280 (13)	−0.0043 (13)
N4	0.0455 (13)	0.0318 (13)	0.0524 (14)	−0.0003 (10)	0.0208 (11)	−0.0002 (10)
C5	0.0481 (16)	0.0333 (15)	0.0615 (18)	0.0013 (12)	0.0256 (14)	0.0011 (14)
C6	0.0654 (18)	0.0284 (15)	0.0604 (17)	0.0045 (13)	0.0328 (15)	−0.0014 (14)
C17	0.0566 (17)	0.0328 (16)	0.0586 (18)	0.0029 (14)	0.0225 (14)	−0.0005 (15)
O17	0.120 (2)	0.0344 (12)	0.114 (2)	0.0046 (13)	0.0793 (17)	−0.0058 (13)
C11	0.0488 (16)	0.0260 (14)	0.0577 (17)	0.0045 (12)	0.0187 (13)	0.0013 (13)
C12	0.0504 (18)	0.062 (2)	0.0482 (16)	0.0070 (15)	0.0073 (14)	0.0055 (16)
C13	0.0400 (15)	0.060 (2)	0.066 (2)	−0.0020 (15)	0.0103 (14)	0.0016 (17)
C14	0.0450 (15)	0.0344 (15)	0.0560 (18)	0.0027 (13)	0.0167 (13)	0.0037 (14)
I14	0.05888 (16)	0.0820 (2)	0.07440 (18)	−0.00787 (11)	0.02923 (12)	0.00640 (13)
C15	0.0475 (16)	0.0401 (17)	0.0455 (16)	−0.0031 (13)	0.0068 (13)	0.0017 (13)
C16	0.0424 (15)	0.0332 (16)	0.0593 (18)	−0.0055 (12)	0.0091 (13)	−0.0011 (14)
C41	0.0382 (14)	0.0327 (15)	0.0612 (18)	−0.0036 (11)	0.0192 (13)	0.0059 (14)
C42	0.0418 (15)	0.0348 (16)	0.068 (2)	−0.0022 (12)	0.0208 (14)	0.0052 (15)
C43	0.0592 (19)	0.0376 (18)	0.089 (3)	0.0042 (15)	0.0273 (18)	0.0115 (18)
C44	0.059 (2)	0.051 (2)	0.096 (3)	0.0032 (17)	0.0199 (19)	0.030 (2)
C45	0.059 (2)	0.072 (3)	0.067 (2)	−0.0078 (18)	0.0117 (17)	0.026 (2)
C46	0.0558 (18)	0.052 (2)	0.0573 (19)	−0.0096 (15)	0.0164 (15)	0.0025 (16)
O42	0.0707 (14)	0.0366 (12)	0.0635 (14)	0.0050 (10)	0.0157 (11)	−0.0054 (10)
C47	0.073 (2)	0.051 (2)	0.088 (3)	−0.0028 (17)	0.018 (2)	−0.022 (2)

### 1-(4-Iodobenzoyl)-4-(2-methoxyphenyl)piperazine (IV) Geometric parameters (Å, º)

**Table:**

N1—C17	1.338 (4)	C13—C14	1.366 (4)
N1—C6	1.464 (3)	C13—H13	0.9300
N1—C2	1.464 (4)	C14—C15	1.373 (4)
C2—C3	1.509 (4)	C14—I14	2.104 (3)
C2—H2A	0.9700	C15—C16	1.386 (4)
C2—H2B	0.9700	C15—H15	0.9300
C3—N4	1.457 (3)	C16—H16	0.9300
C3—H3A	0.9700	C41—C46	1.391 (4)
C3—H3B	0.9700	C41—C42	1.410 (4)
N4—C41	1.421 (3)	C42—O42	1.364 (4)
N4—C5	1.469 (3)	C42—C43	1.377 (4)
C5—C6	1.502 (4)	C43—C44	1.385 (5)
C5—H5A	0.9700	C43—H43	0.9300
C5—H5B	0.9700	C44—C45	1.364 (5)
C6—H6A	0.9700	C44—H44	0.9300
C6—H6B	0.9700	C45—C46	1.383 (4)
C17—O17	1.223 (3)	C45—H45	0.9300
C17—C11	1.502 (4)	C46—H46	0.9300
C11—C12	1.384 (4)	O42—C47	1.417 (4)
C11—C16	1.384 (4)	C47—H47A	0.9600
C12—C13	1.381 (4)	C47—H47B	0.9600
C12—H12	0.9300	C47—H47C	0.9600
			
C17—N1—C6	125.0 (2)	C11—C12—H12	119.8
C17—N1—C2	121.4 (2)	C14—C13—C12	120.2 (3)
C6—N1—C2	113.6 (2)	C14—C13—H13	119.9
N1—C2—C3	110.9 (2)	C12—C13—H13	119.9
N1—C2—H2A	109.5	C13—C14—C15	120.6 (3)
C3—C2—H2A	109.5	C13—C14—I14	118.5 (2)
N1—C2—H2B	109.5	C15—C14—I14	120.9 (2)
C3—C2—H2B	109.5	C14—C15—C16	119.2 (3)
H2A—C2—H2B	108.0	C14—C15—H15	120.4
N4—C3—C2	109.5 (2)	C16—C15—H15	120.4
N4—C3—H3A	109.8	C11—C16—C15	121.0 (3)
C2—C3—H3A	109.8	C11—C16—H16	119.5
N4—C3—H3B	109.8	C15—C16—H16	119.5
C2—C3—H3B	109.8	C46—C41—C42	118.1 (3)
H3A—C3—H3B	108.2	C46—C41—N4	124.3 (3)
C41—N4—C3	116.5 (2)	C42—C41—N4	117.6 (3)
C41—N4—C5	112.8 (2)	O42—C42—C43	124.0 (3)
C3—N4—C5	110.4 (2)	O42—C42—C41	115.5 (2)
N4—C5—C6	109.7 (2)	C43—C42—C41	120.5 (3)
N4—C5—H5A	109.7	C42—C43—C44	119.8 (3)
C6—C5—H5A	109.7	C42—C43—H43	120.1
N4—C5—H5B	109.7	C44—C43—H43	120.1
C6—C5—H5B	109.7	C45—C44—C43	120.6 (3)
H5A—C5—H5B	108.2	C45—C44—H44	119.7
N1—C6—C5	110.2 (2)	C43—C44—H44	119.7
N1—C6—H6A	109.6	C44—C45—C46	120.2 (3)
C5—C6—H6A	109.6	C44—C45—H45	119.9
N1—C6—H6B	109.6	C46—C45—H45	119.9
C5—C6—H6B	109.6	C45—C46—C41	120.8 (3)
H6A—C6—H6B	108.1	C45—C46—H46	119.6
O17—C17—N1	122.8 (3)	C41—C46—H46	119.6
O17—C17—C11	119.7 (3)	C42—O42—C47	118.3 (2)
N1—C17—C11	117.4 (2)	O42—C47—H47A	109.5
C12—C11—C16	118.6 (3)	O42—C47—H47B	109.5
C12—C11—C17	118.6 (3)	H47A—C47—H47B	109.5
C16—C11—C17	122.8 (3)	O42—C47—H47C	109.5
C13—C12—C11	120.5 (3)	H47A—C47—H47C	109.5
C13—C12—H12	119.8	H47B—C47—H47C	109.5
			
C17—N1—C2—C3	126.9 (3)	C13—C14—C15—C16	0.5 (5)
C6—N1—C2—C3	−52.7 (3)	I14—C14—C15—C16	−178.4 (2)
N1—C2—C3—N4	55.4 (3)	C12—C11—C16—C15	−2.3 (4)
C2—C3—N4—C41	168.9 (2)	C17—C11—C16—C15	−178.7 (3)
C2—C3—N4—C5	−60.7 (3)	C14—C15—C16—C11	1.0 (4)
C41—N4—C5—C6	−166.1 (2)	C3—N4—C41—C46	19.4 (4)
C3—N4—C5—C6	61.6 (3)	C5—N4—C41—C46	−109.9 (3)
C17—N1—C6—C5	−126.5 (3)	C3—N4—C41—C42	−159.7 (2)
C2—N1—C6—C5	53.1 (3)	C5—N4—C41—C42	71.1 (3)
N4—C5—C6—N1	−56.4 (3)	C46—C41—C42—O42	−177.7 (2)
C6—N1—C17—O17	178.3 (3)	N4—C41—C42—O42	1.4 (4)
C2—N1—C17—O17	−1.3 (5)	C46—C41—C42—C43	1.8 (4)
C6—N1—C17—C11	0.1 (4)	N4—C41—C42—C43	−179.1 (2)
C2—N1—C17—C11	−179.5 (3)	O42—C42—C43—C44	178.8 (3)
O17—C17—C11—C12	−78.4 (4)	C41—C42—C43—C44	−0.7 (4)
N1—C17—C11—C12	99.8 (3)	C42—C43—C44—C45	−0.8 (5)
O17—C17—C11—C16	97.9 (4)	C43—C44—C45—C46	1.1 (5)
N1—C17—C11—C16	−83.9 (4)	C44—C45—C46—C41	0.1 (5)
C16—C11—C12—C13	2.0 (4)	C42—C41—C46—C45	−1.5 (4)
C17—C11—C12—C13	178.6 (3)	N4—C41—C46—C45	179.4 (3)
C11—C12—C13—C14	−0.5 (5)	C43—C42—O42—C47	−1.1 (4)
C12—C13—C14—C15	−0.8 (5)	C41—C42—O42—C47	178.3 (3)
C12—C13—C14—I14	178.2 (2)		

### 1-(4-Iodobenzoyl)-4-(2-methoxyphenyl)piperazine (IV) Hydrogen-bond geometry (Å, º)

**Table:**

*D*—H···*A*	*D*—H	H···*A*	*D*···*A*	*D*—H···*A*
C3—H3*A*···O17^i^	0.97	2.50	3.422 (4)	159

Symmetry code: (i) −*x*+1, *y*+1/2, −*z*+1/2.

### 1-(3-Iodobenzoyl)-4-(2-methoxyphenyl)piperazine (V) Crystal data

**Table:**

C_18_H_19_IN_2_O_2_	*D*_x_ = 1.575 Mg m^−^^3^
*M**_r_* = 422.25	Mo *K*α radiation, λ = 0.71073 Å
Orthorhombic, *P*2_1_2_1_2_1_	Cell parameters from 7668 reflections
*a* = 7.4528 (4) Å	θ = 2.8–27.9°
*b* = 17.1306 (9) Å	µ = 1.81 mm^−^^1^
*c* = 27.903 (1) Å	*T* = 296 K
*V* = 3562.4 (3) Å^3^	Block, orange
*Z* = 8	0.36 × 0.22 × 0.18 mm
*F*(000) = 1680	

### 1-(3-Iodobenzoyl)-4-(2-methoxyphenyl)piperazine (V) Data collection

**Table:**

Oxford Diffraction Xcalibur CCD diffractometer	7653 independent reflections
Radiation source: Enhance (Mo) X-ray Source	5048 reflections with *I* > 2σ(*I*)
Graphite monochromator	*R*_int_ = 0.025
ω scans	θ_max_ = 27.5°, θ_min_ = 2.8°
Absorption correction: multi-scan (CrysalisRed; Oxford Diffraction, 2009)	*h* = −5→9
*T*_min_ = 0.542, *T*_max_ = 0.722	*k* = −22→15
13774 measured reflections	*l* = −29→36

### 1-(3-Iodobenzoyl)-4-(2-methoxyphenyl)piperazine (V) Refinement

**Table:**

Refinement on *F*^2^	Hydrogen site location: inferred from neighbouring sites
Least-squares matrix: full	H-atom parameters constrained
*R*[*F*^2^ > 2σ(*F*^2^)] = 0.048	*w* = 1/[σ^2^(*F*_o_^2^) + (0.0552*P*)^2^ + 0.7763*P*] where *P* = (*F*_o_^2^ + 2*F*_c_^2^)/3
*wR*(*F*^2^) = 0.116	(Δ/σ)_max_ = 0.001
*S* = 1.04	Δρ_max_ = 1.12 e Å^−^^3^
7653 reflections	Δρ_min_ = −0.69 e Å^−^^3^
417 parameters	Absolute structure: Flack *x* determined using 1728 quotients [(*I*^+^)-(*I*^-^)]/[(*I*^+^)+(*I*^-^)] (Parsons *et al.*, 2013)
0 restraints	Absolute structure parameter: 0.456 (12)
Primary atom site location: difference Fourier map	

### 1-(3-Iodobenzoyl)-4-(2-methoxyphenyl)piperazine (V) Special details

**Table:**

Geometry. All esds (except the esd in the dihedral angle between two l.s. planes) are estimated using the full covariance matrix. The cell esds are taken into account individually in the estimation of esds in distances, angles and torsion angles; correlations between esds in cell parameters are only used when they are defined by crystal symmetry. An approximate (isotropic) treatment of cell esds is used for estimating esds involving l.s. planes.

### 1-(3-Iodobenzoyl)-4-(2-methoxyphenyl)piperazine (V) Fractional atomic coordinates and isotropic or equivalent isotropic displacement parameters (Å^2^)

**Table:**

	*x*	*y*	*z*	*U*_iso_*/*U*_eq_	
N11	0.2358 (9)	0.4763 (4)	0.2308 (2)	0.0432 (17)	
C12	0.4162 (11)	0.5077 (5)	0.2385 (3)	0.046 (2)	
H12A	0.4546	0.4963	0.2710	0.055*	
H12B	0.4139	0.5640	0.2346	0.055*	
C13	0.5480 (11)	0.4728 (5)	0.2034 (3)	0.046 (2)	
H13A	0.6646	0.4971	0.2073	0.056*	
H13B	0.5608	0.4173	0.2095	0.056*	
N14	0.4820 (8)	0.4855 (3)	0.1547 (2)	0.0371 (15)	
C15	0.3113 (11)	0.4474 (5)	0.1485 (3)	0.047 (2)	
H15A	0.3233	0.3922	0.1555	0.057*	
H15B	0.2724	0.4529	0.1155	0.057*	
C16	0.1750 (11)	0.4825 (5)	0.1809 (3)	0.049 (2)	
H16A	0.1570	0.5369	0.1727	0.059*	
H16B	0.0614	0.4555	0.1772	0.059*	
C117	0.1183 (12)	0.4600 (4)	0.2664 (3)	0.0401 (18)	
O117	−0.0429 (8)	0.4514 (4)	0.2587 (2)	0.0657 (17)	
C111	0.1887 (10)	0.4497 (4)	0.3164 (3)	0.0406 (19)	
C112	0.1335 (10)	0.4990 (4)	0.3524 (3)	0.0345 (17)	
H112	0.0589	0.5411	0.3456	0.041*	
C113	0.1897 (10)	0.4853 (4)	0.3985 (3)	0.0386 (18)	
I113	0.09926 (11)	0.55646 (4)	0.45473 (2)	0.0712 (2)	
C114	0.3004 (11)	0.4230 (4)	0.4104 (3)	0.044 (2)	
H114	0.3372	0.4147	0.4419	0.053*	
C115	0.3544 (11)	0.3733 (5)	0.3732 (3)	0.051 (2)	
H115	0.4285	0.3310	0.3799	0.061*	
C116	0.2999 (11)	0.3861 (5)	0.3274 (3)	0.047 (2)	
H116	0.3365	0.3524	0.3032	0.056*	
C141	0.6137 (12)	0.4734 (4)	0.1186 (3)	0.0407 (18)	
C142	0.7486 (11)	0.5304 (5)	0.1124 (3)	0.048 (2)	
C143	0.8713 (13)	0.5237 (6)	0.0762 (3)	0.064 (3)	
H143	0.9604	0.5613	0.0728	0.077*	
C144	0.8645 (16)	0.4629 (7)	0.0452 (3)	0.078 (3)	
H144	0.9494	0.4590	0.0208	0.093*	
C145	0.7317 (15)	0.4064 (7)	0.0495 (4)	0.072 (3)	
H145	0.7257	0.3652	0.0278	0.087*	
C146	0.6085 (14)	0.4123 (5)	0.0863 (3)	0.054 (2)	
H146	0.5202	0.3742	0.0895	0.064*	
O142	0.7427 (8)	0.5911 (3)	0.1439 (2)	0.0599 (17)	
C147	0.8367 (13)	0.6603 (6)	0.1330 (4)	0.075 (3)	
H17A	0.7972	0.7014	0.1539	0.113*	
H17B	0.8140	0.6747	0.1004	0.113*	
H17C	0.9630	0.6519	0.1375	0.113*	
N21	0.2574 (9)	0.7192 (4)	0.2625 (2)	0.0410 (16)	
C22	0.3201 (11)	0.7090 (5)	0.3111 (3)	0.046 (2)	
H22A	0.4368	0.7335	0.3146	0.055*	
H22B	0.3337	0.6538	0.3178	0.055*	
C23	0.1894 (12)	0.7449 (5)	0.3471 (3)	0.050 (2)	
H23A	0.2306	0.7351	0.3795	0.059*	
H23B	0.1824	0.8009	0.3424	0.059*	
N24	0.0133 (8)	0.7098 (4)	0.3400 (2)	0.0370 (15)	
C25	−0.0519 (10)	0.7253 (5)	0.2915 (3)	0.0403 (19)	
H25A	−0.0619	0.7812	0.2867	0.048*	
H25B	−0.1702	0.7025	0.2876	0.048*	
C26	0.0736 (10)	0.6914 (5)	0.2548 (3)	0.043 (2)	
H26A	0.0712	0.6349	0.2568	0.052*	
H26B	0.0340	0.7062	0.2230	0.052*	
C217	0.3746 (11)	0.7340 (4)	0.2273 (3)	0.0384 (17)	
O217	0.5362 (8)	0.7387 (4)	0.2348 (2)	0.0592 (17)	
C211	0.3042 (10)	0.7458 (4)	0.1784 (3)	0.0360 (17)	
C212	0.3596 (11)	0.6946 (4)	0.1411 (3)	0.0387 (18)	
H212	0.4307	0.6513	0.1476	0.046*	
C213	0.3053 (11)	0.7109 (4)	0.0950 (3)	0.0422 (19)	
I213	0.38276 (10)	0.63124 (4)	0.04062 (2)	0.0694 (2)	
C214	0.2027 (12)	0.7747 (5)	0.0834 (3)	0.051 (2)	
H214	0.1679	0.7842	0.0520	0.062*	
C215	0.1535 (12)	0.8239 (5)	0.1198 (3)	0.053 (2)	
H215	0.0857	0.8679	0.1126	0.063*	
C216	0.2007 (11)	0.8103 (5)	0.1664 (3)	0.047 (2)	
H216	0.1633	0.8446	0.1902	0.057*	
C241	−0.1152 (11)	0.7211 (4)	0.3769 (2)	0.0396 (17)	
C242	−0.2523 (11)	0.6650 (5)	0.3815 (3)	0.046 (2)	
C243	−0.3767 (13)	0.6729 (6)	0.4185 (3)	0.063 (3)	
H243	−0.4674	0.6360	0.4217	0.075*	
C244	−0.3675 (14)	0.7364 (6)	0.4516 (3)	0.067 (3)	
H244	−0.4552	0.7438	0.4749	0.080*	
C245	−0.2271 (16)	0.7849 (7)	0.4476 (3)	0.077 (3)	
H245	−0.2116	0.8235	0.4707	0.093*	
C246	−0.1052 (14)	0.7798 (5)	0.4108 (3)	0.053 (2)	
H246	−0.0139	0.8166	0.4086	0.064*	
O242	−0.2497 (8)	0.6053 (3)	0.3494 (2)	0.0570 (17)	
C247	−0.3468 (13)	0.5376 (5)	0.3602 (4)	0.077 (3)	
H27A	−0.3146	0.4969	0.3382	0.115*	
H27B	−0.4729	0.5482	0.3577	0.115*	
H27C	−0.3194	0.5212	0.3923	0.115*	

### 1-(3-Iodobenzoyl)-4-(2-methoxyphenyl)piperazine (V) Atomic displacement parameters (Å^2^)

**Table:**

	*U*^11^	*U*^22^	*U*^33^	*U*^12^	*U*^13^	*U*^23^
N11	0.035 (4)	0.062 (4)	0.033 (4)	−0.011 (3)	−0.001 (3)	0.001 (3)
C12	0.037 (5)	0.061 (5)	0.039 (4)	−0.013 (4)	0.000 (4)	−0.005 (4)
C13	0.038 (5)	0.056 (5)	0.045 (5)	−0.005 (4)	−0.004 (4)	0.000 (4)
N14	0.035 (4)	0.044 (4)	0.032 (3)	−0.005 (3)	−0.002 (3)	−0.001 (3)
C15	0.047 (5)	0.059 (5)	0.037 (4)	−0.005 (4)	−0.004 (4)	−0.014 (4)
C16	0.026 (5)	0.073 (6)	0.049 (5)	−0.010 (4)	−0.008 (4)	0.006 (4)
C117	0.035 (5)	0.041 (4)	0.044 (4)	0.008 (4)	−0.001 (4)	0.003 (3)
O117	0.037 (4)	0.100 (5)	0.060 (4)	−0.005 (3)	−0.002 (3)	0.011 (3)
C111	0.029 (4)	0.039 (4)	0.054 (5)	−0.001 (3)	0.012 (4)	−0.003 (4)
C112	0.028 (4)	0.029 (4)	0.047 (4)	−0.002 (3)	−0.001 (4)	0.002 (3)
C113	0.031 (4)	0.038 (4)	0.047 (5)	0.001 (3)	0.004 (4)	−0.008 (3)
I113	0.0901 (5)	0.0754 (4)	0.0481 (3)	0.0307 (4)	−0.0073 (4)	−0.0164 (3)
C114	0.040 (5)	0.050 (5)	0.043 (5)	0.004 (4)	−0.003 (4)	0.012 (4)
C115	0.042 (5)	0.046 (4)	0.063 (5)	0.010 (4)	0.004 (5)	0.003 (4)
C116	0.047 (5)	0.048 (5)	0.046 (5)	0.007 (4)	0.011 (4)	0.007 (4)
C141	0.037 (5)	0.047 (4)	0.039 (4)	0.008 (4)	−0.003 (4)	0.001 (3)
C142	0.035 (5)	0.061 (6)	0.047 (5)	0.011 (4)	0.000 (4)	−0.001 (4)
C143	0.045 (6)	0.077 (6)	0.070 (6)	0.010 (5)	0.017 (6)	0.015 (5)
C144	0.070 (8)	0.123 (9)	0.040 (5)	0.047 (7)	0.014 (7)	0.012 (6)
C145	0.068 (7)	0.097 (8)	0.052 (6)	0.037 (6)	−0.013 (6)	−0.024 (6)
C146	0.056 (6)	0.062 (5)	0.042 (4)	0.010 (5)	−0.005 (5)	−0.005 (4)
O142	0.047 (4)	0.057 (4)	0.076 (4)	−0.008 (3)	0.009 (3)	−0.003 (3)
C147	0.056 (7)	0.081 (8)	0.089 (8)	−0.026 (5)	−0.011 (6)	0.019 (6)
N21	0.032 (4)	0.062 (4)	0.029 (3)	−0.007 (3)	0.002 (3)	0.003 (3)
C22	0.033 (5)	0.069 (5)	0.037 (4)	−0.005 (4)	−0.002 (4)	0.002 (4)
C23	0.041 (5)	0.059 (6)	0.048 (5)	−0.009 (4)	−0.008 (4)	0.002 (4)
N24	0.028 (4)	0.049 (4)	0.034 (4)	0.001 (3)	−0.001 (3)	0.005 (3)
C25	0.029 (4)	0.054 (5)	0.037 (4)	−0.003 (3)	−0.002 (4)	0.008 (4)
C26	0.036 (5)	0.061 (5)	0.033 (4)	−0.014 (4)	−0.001 (4)	0.000 (3)
C217	0.034 (5)	0.038 (4)	0.044 (4)	−0.007 (4)	0.005 (4)	0.001 (3)
O217	0.033 (4)	0.090 (5)	0.055 (4)	−0.011 (3)	−0.003 (3)	0.018 (3)
C211	0.027 (4)	0.040 (4)	0.040 (4)	−0.007 (3)	0.007 (4)	0.003 (3)
C212	0.031 (5)	0.046 (5)	0.039 (4)	−0.001 (4)	0.002 (4)	0.006 (3)
C213	0.032 (4)	0.047 (5)	0.048 (5)	0.002 (4)	0.004 (4)	−0.004 (4)
I213	0.0749 (5)	0.0825 (4)	0.0509 (4)	0.0113 (4)	−0.0022 (4)	−0.0170 (3)
C214	0.044 (5)	0.065 (6)	0.046 (5)	−0.002 (4)	−0.007 (4)	0.013 (4)
C215	0.052 (6)	0.046 (5)	0.059 (6)	0.021 (4)	0.006 (5)	0.012 (4)
C216	0.038 (5)	0.048 (5)	0.056 (6)	0.007 (4)	0.009 (4)	−0.006 (4)
C241	0.038 (5)	0.051 (4)	0.030 (4)	0.012 (4)	0.003 (4)	0.007 (3)
C242	0.034 (5)	0.054 (5)	0.049 (5)	0.003 (4)	0.006 (4)	0.012 (4)
C243	0.040 (5)	0.085 (6)	0.063 (6)	0.019 (5)	0.011 (5)	0.033 (5)
C244	0.050 (6)	0.106 (8)	0.044 (5)	0.023 (6)	0.014 (6)	0.022 (5)
C245	0.083 (9)	0.110 (9)	0.039 (5)	0.052 (7)	0.007 (6)	−0.012 (5)
C246	0.057 (6)	0.057 (5)	0.046 (4)	0.018 (5)	−0.008 (5)	−0.004 (4)
O242	0.049 (4)	0.048 (4)	0.074 (4)	−0.013 (3)	0.004 (3)	−0.001 (3)
C247	0.048 (6)	0.055 (6)	0.128 (10)	−0.022 (5)	−0.016 (6)	0.024 (6)

### 1-(3-Iodobenzoyl)-4-(2-methoxyphenyl)piperazine (V) Geometric parameters (Å, º)

**Table:**

N11—C117	1.352 (10)	N21—C217	1.339 (9)
N11—C12	1.464 (10)	N21—C22	1.445 (9)
N11—C16	1.468 (10)	N21—C26	1.466 (9)
C12—C13	1.511 (11)	C22—C23	1.529 (12)
C12—H12A	0.9700	C22—H22A	0.9700
C12—H12B	0.9700	C22—H22B	0.9700
C13—N14	1.462 (9)	C23—N24	1.457 (10)
C13—H13A	0.9700	C23—H23A	0.9700
C13—H13B	0.9700	C23—H23B	0.9700
N14—C141	1.422 (9)	N24—C241	1.420 (9)
N14—C15	1.440 (10)	N24—C25	1.461 (9)
C15—C16	1.487 (11)	C25—C26	1.504 (10)
C15—H15A	0.9700	C25—H25A	0.9700
C15—H15B	0.9700	C25—H25B	0.9700
C16—H16A	0.9700	C26—H26A	0.9700
C16—H16B	0.9700	C26—H26B	0.9700
C117—O117	1.229 (10)	C217—O217	1.225 (9)
C117—C111	1.503 (11)	C217—C211	1.476 (10)
C111—C112	1.374 (10)	C211—C216	1.389 (11)
C111—C116	1.403 (11)	C211—C212	1.423 (10)
C112—C113	1.373 (10)	C212—C213	1.375 (10)
C112—H112	0.9300	C212—H212	0.9300
C113—C114	1.389 (10)	C213—C214	1.372 (11)
C113—I113	2.098 (7)	C213—I213	2.121 (8)
C114—C115	1.404 (11)	C214—C215	1.369 (11)
C114—H114	0.9300	C214—H214	0.9300
C115—C116	1.357 (11)	C215—C216	1.366 (11)
C115—H115	0.9300	C215—H215	0.9300
C116—H116	0.9300	C216—H216	0.9300
C141—C146	1.381 (10)	C241—C246	1.382 (10)
C141—C142	1.412 (11)	C241—C242	1.409 (11)
C142—O142	1.362 (10)	C242—O242	1.359 (10)
C142—C143	1.368 (12)	C242—C243	1.395 (12)
C143—C144	1.355 (13)	C243—C244	1.429 (13)
C143—H143	0.9300	C243—H243	0.9300
C144—C145	1.390 (15)	C244—C245	1.340 (14)
C144—H144	0.9300	C244—H244	0.9300
C145—C146	1.383 (13)	C245—C246	1.375 (12)
C145—H145	0.9300	C245—H245	0.9300
C146—H146	0.9300	C246—H246	0.9300
O142—C147	1.410 (10)	O242—C247	1.400 (9)
C147—H17A	0.9600	C247—H27A	0.9600
C147—H17B	0.9600	C247—H27B	0.9600
C147—H17C	0.9600	C247—H27C	0.9600
			
C117—N11—C12	124.3 (7)	C217—N21—C22	120.0 (7)
C117—N11—C16	120.7 (7)	C217—N21—C26	124.3 (7)
C12—N11—C16	113.3 (6)	C22—N21—C26	113.6 (6)
N11—C12—C13	110.9 (6)	N21—C22—C23	111.2 (7)
N11—C12—H12A	109.5	N21—C22—H22A	109.4
C13—C12—H12A	109.5	C23—C22—H22A	109.4
N11—C12—H12B	109.5	N21—C22—H22B	109.4
C13—C12—H12B	109.5	C23—C22—H22B	109.4
H12A—C12—H12B	108.1	H22A—C22—H22B	108.0
N14—C13—C12	109.0 (7)	N24—C23—C22	108.6 (7)
N14—C13—H13A	109.9	N24—C23—H23A	110.0
C12—C13—H13A	109.9	C22—C23—H23A	110.0
N14—C13—H13B	109.9	N24—C23—H23B	110.0
C12—C13—H13B	109.9	C22—C23—H23B	110.0
H13A—C13—H13B	108.3	H23A—C23—H23B	108.3
C141—N14—C15	117.3 (6)	C241—N24—C23	116.9 (6)
C141—N14—C13	113.9 (6)	C241—N24—C25	115.0 (6)
C15—N14—C13	110.0 (6)	C23—N24—C25	110.5 (6)
N14—C15—C16	110.4 (6)	N24—C25—C26	110.7 (6)
N14—C15—H15A	109.6	N24—C25—H25A	109.5
C16—C15—H15A	109.6	C26—C25—H25A	109.5
N14—C15—H15B	109.6	N24—C25—H25B	109.5
C16—C15—H15B	109.6	C26—C25—H25B	109.5
H15A—C15—H15B	108.1	H25A—C25—H25B	108.1
N11—C16—C15	109.7 (7)	N21—C26—C25	110.9 (6)
N11—C16—H16A	109.7	N21—C26—H26A	109.5
C15—C16—H16A	109.7	C25—C26—H26A	109.5
N11—C16—H16B	109.7	N21—C26—H26B	109.5
C15—C16—H16B	109.7	C25—C26—H26B	109.5
H16A—C16—H16B	108.2	H26A—C26—H26B	108.1
O117—C117—N11	122.0 (7)	O217—C217—N21	121.9 (7)
O117—C117—C111	119.2 (7)	O217—C217—C211	120.0 (7)
N11—C117—C111	118.7 (7)	N21—C217—C211	118.1 (7)
C112—C111—C116	119.7 (8)	C216—C211—C212	118.3 (7)
C112—C111—C117	120.1 (7)	C216—C211—C217	122.0 (7)
C116—C111—C117	120.0 (7)	C212—C211—C217	119.3 (7)
C113—C112—C111	119.2 (7)	C213—C212—C211	118.3 (7)
C113—C112—H112	120.4	C213—C212—H212	120.9
C111—C112—H112	120.4	C211—C212—H212	120.9
C112—C113—C114	122.5 (7)	C214—C213—C212	123.1 (7)
C112—C113—I113	120.2 (5)	C214—C213—I213	119.7 (6)
C114—C113—I113	117.2 (6)	C212—C213—I213	117.2 (6)
C113—C114—C115	117.3 (8)	C215—C214—C213	117.7 (8)
C113—C114—H114	121.3	C215—C214—H214	121.1
C115—C114—H114	121.3	C213—C214—H214	121.1
C116—C115—C114	120.8 (8)	C216—C215—C214	122.0 (8)
C116—C115—H115	119.6	C216—C215—H215	119.0
C114—C115—H115	119.6	C214—C215—H215	119.0
C115—C116—C111	120.5 (8)	C215—C216—C211	120.6 (8)
C115—C116—H116	119.7	C215—C216—H216	119.7
C111—C116—H116	119.7	C211—C216—H216	119.7
C146—C141—C142	117.7 (8)	C246—C241—C242	118.3 (8)
C146—C141—N14	123.5 (8)	C246—C241—N24	124.0 (8)
C142—C141—N14	118.5 (7)	C242—C241—N24	117.5 (7)
O142—C142—C143	124.3 (8)	O242—C242—C243	124.8 (8)
O142—C142—C141	115.2 (7)	O242—C242—C241	116.3 (7)
C143—C142—C141	120.5 (8)	C243—C242—C241	118.9 (8)
C144—C143—C142	120.8 (10)	C242—C243—C244	121.4 (9)
C144—C143—H143	119.6	C242—C243—H243	119.3
C142—C143—H143	119.6	C244—C243—H243	119.3
C143—C144—C145	120.4 (10)	C245—C244—C243	117.1 (9)
C143—C144—H144	119.8	C245—C244—H244	121.5
C145—C144—H144	119.8	C243—C244—H244	121.5
C146—C145—C144	119.1 (10)	C244—C245—C246	122.6 (10)
C146—C145—H145	120.4	C244—C245—H245	118.7
C144—C145—H145	120.4	C246—C245—H245	118.7
C141—C146—C145	121.4 (10)	C245—C246—C241	121.5 (10)
C141—C146—H146	119.3	C245—C246—H246	119.3
C145—C146—H146	119.3	C241—C246—H246	119.3
C142—O142—C147	119.1 (8)	C242—O242—C247	118.3 (8)
O142—C147—H17A	109.5	O242—C247—H27A	109.5
O142—C147—H17B	109.5	O242—C247—H27B	109.5
H17A—C147—H17B	109.5	H27A—C247—H27B	109.5
O142—C147—H17C	109.5	O242—C247—H27C	109.5
H17A—C147—H17C	109.5	H27A—C247—H27C	109.5
H17B—C147—H17C	109.5	H27B—C247—H27C	109.5
			
C117—N11—C12—C13	142.6 (7)	C217—N21—C22—C23	142.6 (7)
C16—N11—C12—C13	−52.2 (9)	C26—N21—C22—C23	−53.0 (9)
N11—C12—C13—N14	55.0 (9)	N21—C22—C23—N24	56.7 (9)
C12—C13—N14—C141	164.7 (6)	C22—C23—N24—C241	165.3 (6)
C12—C13—N14—C15	−61.2 (8)	C22—C23—N24—C25	−60.6 (8)
C141—N14—C15—C16	−164.3 (6)	C241—N24—C25—C26	−164.5 (6)
C13—N14—C15—C16	63.4 (9)	C23—N24—C25—C26	60.4 (8)
C117—N11—C16—C15	−141.4 (7)	C217—N21—C26—C25	−145.0 (7)
C12—N11—C16—C15	52.8 (9)	C22—N21—C26—C25	51.4 (9)
N14—C15—C16—N11	−57.7 (9)	N24—C25—C26—N21	−54.1 (9)
C12—N11—C117—O117	162.6 (8)	C22—N21—C217—O217	0.6 (12)
C16—N11—C117—O117	−1.6 (11)	C26—N21—C217—O217	−162.1 (7)
C12—N11—C117—C111	−19.3 (11)	C22—N21—C217—C211	−178.6 (6)
C16—N11—C117—C111	176.5 (7)	C26—N21—C217—C211	18.8 (11)
O117—C117—C111—C112	−63.4 (10)	O217—C217—C211—C216	−111.5 (9)
N11—C117—C111—C112	118.4 (8)	N21—C217—C211—C216	67.6 (10)
O117—C117—C111—C116	111.6 (9)	O217—C217—C211—C212	61.3 (10)
N11—C117—C111—C116	−66.6 (10)	N21—C217—C211—C212	−119.5 (8)
C116—C111—C112—C113	0.6 (11)	C216—C211—C212—C213	−1.1 (11)
C117—C111—C112—C113	175.5 (7)	C217—C211—C212—C213	−174.2 (7)
C111—C112—C113—C114	−0.3 (12)	C211—C212—C213—C214	1.0 (12)
C111—C112—C113—I113	−177.4 (5)	C211—C212—C213—I213	−177.9 (5)
C112—C113—C114—C115	0.0 (12)	C212—C213—C214—C215	0.1 (13)
I113—C113—C114—C115	177.2 (6)	I213—C213—C214—C215	179.0 (6)
C113—C114—C115—C116	0.0 (13)	C213—C214—C215—C216	−1.1 (14)
C114—C115—C116—C111	0.3 (13)	C214—C215—C216—C211	1.0 (14)
C112—C111—C116—C115	−0.6 (12)	C212—C211—C216—C215	0.2 (12)
C117—C111—C116—C115	−175.6 (8)	C217—C211—C216—C215	173.0 (8)
C15—N14—C141—C146	−19.2 (11)	C23—N24—C241—C246	20.0 (10)
C13—N14—C141—C146	111.4 (8)	C25—N24—C241—C246	−112.1 (8)
C15—N14—C141—C142	155.1 (7)	C23—N24—C241—C242	−154.4 (7)
C13—N14—C141—C142	−74.3 (9)	C25—N24—C241—C242	73.5 (8)
C146—C141—C142—O142	177.3 (7)	C246—C241—C242—O242	−175.5 (7)
N14—C141—C142—O142	2.7 (10)	N24—C241—C242—O242	−0.8 (10)
C146—C141—C142—C143	−1.3 (12)	C246—C241—C242—C243	2.9 (11)
N14—C141—C142—C143	−175.9 (7)	N24—C241—C242—C243	177.6 (7)
O142—C142—C143—C144	−177.7 (8)	O242—C242—C243—C244	178.3 (8)
C141—C142—C143—C144	0.9 (14)	C241—C242—C243—C244	0.1 (12)
C142—C143—C144—C145	0.4 (15)	C242—C243—C244—C245	−4.7 (13)
C143—C144—C145—C146	−1.2 (15)	C243—C244—C245—C246	6.4 (14)
C142—C141—C146—C145	0.5 (13)	C244—C245—C246—C241	−3.6 (14)
N14—C141—C146—C145	174.9 (7)	C242—C241—C246—C245	−1.3 (12)
C144—C145—C146—C141	0.7 (14)	N24—C241—C246—C245	−175.7 (7)
C143—C142—O142—C147	16.0 (12)	C243—C242—O242—C247	−16.5 (12)
C141—C142—O142—C147	−162.5 (7)	C241—C242—O242—C247	161.7 (7)

### 1-(3-Iodobenzoyl)-4-(2-methoxyphenyl)piperazine (V) Hydrogen-bond geometry (Å, º)

**Table:**

*D*—H···*A*	*D*—H	H···*A*	*D*···*A*	*D*—H···*A*
C112—H112···O242	0.93	2.55	3.388 (9)	150
C116—H116···O217^i^	0.93	2.41	3.301 (10)	159
C212—H212···O142	0.93	2.55	3.363 (9)	147
C216—H216···O117^ii^	0.93	2.49	3.407 (11)	169
C115—H115···*Cg*6^i^	0.93	2.67	3.505 (9)	149
C215—H215···*Cg*7^ii^	0.93	2.81	3.566 (9)	140

Symmetry codes: (i) −*x*+1, *y*−1/2, −*z*+1/2; (ii) −*x*, *y*+1/2, −*z*+1/2.

### 1-(2-Fluorobenzoyl)-4-(2-methoxyphenyl)piperazine (VI) Crystal data

**Table:**

C_18_H_19_FN_2_O_2_	*F*(000) = 664
*M**_r_* = 314.35	*D*_x_ = 1.326 Mg m^−^^3^
Monoclinic, *P*2_1_/*n*	Mo *K*α radiation, λ = 0.71073 Å
*a* = 7.451 (1) Å	Cell parameters from 3467 reflections
*b* = 11.199 (3) Å	θ = 2.8–27.9°
*c* = 19.138 (5) Å	µ = 0.10 mm^−^^1^
β = 99.59 (2)°	*T* = 296 K
*V* = 1574.6 (6) Å^3^	Plate, yellow
*Z* = 4	0.48 × 0.48 × 0.24 mm

### 1-(2-Fluorobenzoyl)-4-(2-methoxyphenyl)piperazine (VI) Data collection

**Table:**

Oxford Diffraction Xcalibur CCD diffractometer	3467 independent reflections
Radiation source: Enhance (Mo) X-ray Source	2081 reflections with *I* > 2σ(*I*)
Graphite monochromator	*R*_int_ = 0.025
ω scans	θ_max_ = 27.9°, θ_min_ = 2.8°
Absorption correction: multi-scan (CrysalisRed; Oxford Diffraction, 2009)	*h* = −9→5
*T*_min_ = 0.898, *T*_max_ = 0.955	*k* = −14→11
6456 measured reflections	*l* = −16→25

### 1-(2-Fluorobenzoyl)-4-(2-methoxyphenyl)piperazine (VI) Refinement

**Table:**

Refinement on *F*^2^	Primary atom site location: difference Fourier map
Least-squares matrix: full	Hydrogen site location: inferred from neighbouring sites
*R*[*F*^2^ > 2σ(*F*^2^)] = 0.049	H-atom parameters constrained
*wR*(*F*^2^) = 0.128	*w* = 1/[σ^2^(*F*_o_^2^) + (0.0558*P*)^2^ + 0.1876*P*] where *P* = (*F*_o_^2^ + 2*F*_c_^2^)/3
*S* = 1.02	(Δ/σ)_max_ < 0.001
3467 reflections	Δρ_max_ = 0.17 e Å^−^^3^
208 parameters	Δρ_min_ = −0.17 e Å^−^^3^
0 restraints	

### 1-(2-Fluorobenzoyl)-4-(2-methoxyphenyl)piperazine (VI) Special details

**Table:**

Geometry. All esds (except the esd in the dihedral angle between two l.s. planes) are estimated using the full covariance matrix. The cell esds are taken into account individually in the estimation of esds in distances, angles and torsion angles; correlations between esds in cell parameters are only used when they are defined by crystal symmetry. An approximate (isotropic) treatment of cell esds is used for estimating esds involving l.s. planes.

### 1-(2-Fluorobenzoyl)-4-(2-methoxyphenyl)piperazine (VI) Fractional atomic coordinates and isotropic or equivalent isotropic displacement parameters (Å^2^)

**Table:**

	*x*	*y*	*z*	*U*_iso_*/*U*_eq_	
N1	0.39928 (19)	0.44822 (13)	0.20339 (9)	0.0579 (4)	
C2	0.2874 (2)	0.35004 (15)	0.17138 (11)	0.0612 (5)	
H2A	0.1763	0.3810	0.1436	0.073*	
H2B	0.2545	0.2988	0.2081	0.073*	
C3	0.3905 (2)	0.27991 (16)	0.12493 (10)	0.0579 (5)	
H3A	0.3172	0.2130	0.1045	0.069*	
H3B	0.4166	0.3300	0.0865	0.069*	
N4	0.56074 (18)	0.23587 (12)	0.16578 (7)	0.0474 (4)	
C5	0.6732 (2)	0.33501 (16)	0.19588 (10)	0.0563 (5)	
H5A	0.7048	0.3843	0.1580	0.068*	
H5B	0.7850	0.3047	0.2234	0.068*	
C6	0.5738 (2)	0.40891 (16)	0.24239 (11)	0.0594 (5)	
H6A	0.5543	0.3622	0.2831	0.071*	
H6B	0.6469	0.4779	0.2594	0.071*	
C17	0.3616 (2)	0.56321 (16)	0.19222 (10)	0.0542 (5)	
O17	0.46610 (18)	0.64224 (11)	0.21579 (8)	0.0794 (5)	
C11	0.1807 (2)	0.59256 (14)	0.14910 (10)	0.0501 (4)	
C12	0.1624 (3)	0.61463 (18)	0.07854 (12)	0.0662 (5)	
F12	0.3121 (2)	0.60631 (15)	0.04794 (8)	0.1099 (5)	
C13	−0.0009 (4)	0.6427 (2)	0.03739 (12)	0.0832 (7)	
H13	−0.0083	0.6570	−0.0109	0.100*	
C14	−0.1508 (3)	0.64914 (19)	0.06847 (14)	0.0803 (7)	
H14	−0.2630	0.6669	0.0413	0.096*	
C15	−0.1387 (3)	0.63003 (18)	0.13860 (14)	0.0716 (6)	
H15	−0.2421	0.6361	0.1597	0.086*	
C16	0.0262 (3)	0.60163 (16)	0.17912 (11)	0.0604 (5)	
H16	0.0330	0.5884	0.2275	0.073*	
C41	0.6480 (2)	0.14823 (15)	0.13071 (9)	0.0474 (4)	
C42	0.5643 (2)	0.03737 (15)	0.11612 (9)	0.0485 (4)	
C43	0.6482 (3)	−0.04929 (17)	0.08283 (10)	0.0619 (5)	
H43	0.5901	−0.1221	0.0719	0.074*	
C44	0.8164 (3)	−0.0300 (2)	0.06540 (12)	0.0757 (6)	
H44	0.8722	−0.0897	0.0429	0.091*	
C45	0.9016 (3)	0.0750 (2)	0.08078 (13)	0.0803 (7)	
H45	1.0172	0.0874	0.0699	0.096*	
C46	0.8171 (3)	0.16411 (19)	0.11264 (11)	0.0655 (5)	
H46	0.8760	0.2369	0.1222	0.079*	
O42	0.40191 (17)	0.02227 (10)	0.13810 (7)	0.0611 (4)	
C47	0.3216 (3)	−0.09241 (18)	0.13149 (14)	0.0790 (7)	
H47A	0.2080	−0.0905	0.1489	0.119*	
H47B	0.3004	−0.1158	0.0825	0.119*	
H47C	0.4018	−0.1488	0.1585	0.119*	

### 1-(2-Fluorobenzoyl)-4-(2-methoxyphenyl)piperazine (VI) Atomic displacement parameters (Å^2^)

**Table:**

	*U*^11^	*U*^22^	*U*^33^	*U*^12^	*U*^13^	*U*^23^
N1	0.0582 (9)	0.0372 (8)	0.0731 (11)	0.0040 (7)	−0.0045 (8)	−0.0065 (7)
C2	0.0549 (10)	0.0353 (9)	0.0878 (14)	0.0019 (8)	−0.0046 (9)	−0.0050 (9)
C3	0.0621 (11)	0.0386 (10)	0.0658 (12)	0.0001 (8)	−0.0100 (9)	−0.0035 (9)
N4	0.0506 (8)	0.0354 (7)	0.0535 (9)	0.0012 (6)	0.0005 (6)	−0.0030 (6)
C5	0.0556 (10)	0.0421 (10)	0.0666 (13)	−0.0011 (8)	−0.0035 (9)	−0.0039 (9)
C6	0.0627 (11)	0.0443 (10)	0.0646 (12)	0.0044 (8)	−0.0089 (9)	−0.0067 (9)
C17	0.0622 (11)	0.0374 (10)	0.0617 (12)	0.0019 (8)	0.0060 (9)	−0.0052 (8)
O17	0.0789 (9)	0.0420 (8)	0.1078 (12)	−0.0044 (7)	−0.0123 (8)	−0.0074 (8)
C11	0.0639 (11)	0.0300 (8)	0.0553 (12)	0.0017 (7)	0.0065 (9)	−0.0025 (8)
C12	0.0794 (14)	0.0551 (12)	0.0662 (14)	0.0059 (10)	0.0182 (11)	−0.0026 (10)
F12	0.1203 (11)	0.1322 (13)	0.0871 (10)	0.0165 (10)	0.0457 (9)	0.0106 (9)
C13	0.1147 (19)	0.0718 (15)	0.0559 (13)	0.0156 (14)	−0.0068 (13)	0.0054 (11)
C14	0.0832 (16)	0.0598 (14)	0.0884 (19)	0.0162 (11)	−0.0137 (13)	−0.0055 (12)
C15	0.0657 (13)	0.0527 (12)	0.0955 (18)	0.0073 (9)	0.0108 (12)	−0.0055 (12)
C16	0.0701 (13)	0.0493 (11)	0.0613 (12)	0.0049 (9)	0.0090 (10)	0.0003 (9)
C41	0.0557 (10)	0.0419 (9)	0.0439 (10)	0.0034 (8)	0.0065 (8)	0.0021 (8)
C42	0.0529 (10)	0.0431 (10)	0.0485 (10)	0.0050 (8)	0.0050 (8)	0.0011 (8)
C43	0.0756 (13)	0.0482 (11)	0.0609 (12)	0.0059 (9)	0.0089 (10)	−0.0102 (9)
C44	0.0900 (16)	0.0676 (15)	0.0767 (15)	0.0144 (12)	0.0351 (13)	−0.0088 (12)
C45	0.0776 (15)	0.0797 (16)	0.0935 (17)	0.0048 (13)	0.0429 (13)	−0.0002 (13)
C46	0.0706 (13)	0.0580 (12)	0.0722 (14)	−0.0045 (10)	0.0241 (10)	0.0009 (10)
O42	0.0608 (8)	0.0385 (7)	0.0858 (10)	−0.0039 (5)	0.0179 (7)	−0.0074 (6)
C47	0.0788 (14)	0.0439 (12)	0.114 (2)	−0.0097 (10)	0.0165 (13)	−0.0006 (12)

### 1-(2-Fluorobenzoyl)-4-(2-methoxyphenyl)piperazine (VI) Geometric parameters (Å, º)

**Table:**

N1—C17	1.328 (2)	C13—C14	1.352 (3)
N1—C2	1.452 (2)	C13—H13	0.9300
N1—C6	1.456 (2)	C14—C15	1.347 (3)
C2—C3	1.492 (3)	C14—H14	0.9300
C2—H2A	0.9700	C15—C16	1.376 (3)
C2—H2B	0.9700	C15—H15	0.9300
C3—N4	1.461 (2)	C16—H16	0.9300
C3—H3A	0.9700	C41—C46	1.372 (2)
C3—H3B	0.9700	C41—C42	1.396 (2)
N4—C41	1.408 (2)	C42—O42	1.356 (2)
N4—C5	1.451 (2)	C42—C43	1.368 (2)
C5—C6	1.498 (2)	C43—C44	1.367 (3)
C5—H5A	0.9700	C43—H43	0.9300
C5—H5B	0.9700	C44—C45	1.346 (3)
C6—H6A	0.9700	C44—H44	0.9300
C6—H6B	0.9700	C45—C46	1.375 (3)
C17—O17	1.215 (2)	C45—H45	0.9300
C17—C11	1.495 (3)	C46—H46	0.9300
C11—C12	1.357 (3)	O42—C47	1.414 (2)
C11—C16	1.374 (2)	C47—H47A	0.9600
C12—F12	1.347 (2)	C47—H47B	0.9600
C12—C13	1.371 (3)	C47—H47C	0.9600
			
C17—N1—C2	125.13 (15)	C11—C12—C13	123.1 (2)
C17—N1—C6	121.51 (15)	C14—C13—C12	118.6 (2)
C2—N1—C6	112.85 (14)	C14—C13—H13	120.7
N1—C2—C3	109.49 (15)	C12—C13—H13	120.7
N1—C2—H2A	109.8	C15—C14—C13	120.4 (2)
C3—C2—H2A	109.8	C15—C14—H14	119.8
N1—C2—H2B	109.8	C13—C14—H14	119.8
C3—C2—H2B	109.8	C14—C15—C16	120.2 (2)
H2A—C2—H2B	108.2	C14—C15—H15	119.9
N4—C3—C2	110.25 (15)	C16—C15—H15	119.9
N4—C3—H3A	109.6	C11—C16—C15	120.9 (2)
C2—C3—H3A	109.6	C11—C16—H16	119.6
N4—C3—H3B	109.6	C15—C16—H16	119.6
C2—C3—H3B	109.6	C46—C41—C42	117.56 (16)
H3A—C3—H3B	108.1	C46—C41—N4	123.10 (16)
C41—N4—C5	116.16 (14)	C42—C41—N4	119.27 (14)
C41—N4—C3	114.14 (14)	O42—C42—C43	123.81 (17)
C5—N4—C3	110.28 (13)	O42—C42—C41	116.13 (14)
N4—C5—C6	110.41 (14)	C43—C42—C41	120.04 (17)
N4—C5—H5A	109.6	C44—C43—C42	120.68 (19)
C6—C5—H5A	109.6	C44—C43—H43	119.7
N4—C5—H5B	109.6	C42—C43—H43	119.7
C6—C5—H5B	109.6	C45—C44—C43	120.19 (19)
H5A—C5—H5B	108.1	C45—C44—H44	119.9
N1—C6—C5	110.49 (15)	C43—C44—H44	119.9
N1—C6—H6A	109.6	C44—C45—C46	119.8 (2)
C5—C6—H6A	109.6	C44—C45—H45	120.1
N1—C6—H6B	109.6	C46—C45—H45	120.1
C5—C6—H6B	109.6	C41—C46—C45	121.7 (2)
H6A—C6—H6B	108.1	C41—C46—H46	119.2
O17—C17—N1	122.81 (17)	C45—C46—H46	119.2
O17—C17—C11	120.51 (16)	C42—O42—C47	118.15 (14)
N1—C17—C11	116.68 (15)	O42—C47—H47A	109.5
C12—C11—C16	116.74 (18)	O42—C47—H47B	109.5
C12—C11—C17	121.44 (17)	H47A—C47—H47B	109.5
C16—C11—C17	121.80 (17)	O42—C47—H47C	109.5
F12—C12—C11	117.80 (19)	H47A—C47—H47C	109.5
F12—C12—C13	119.1 (2)	H47B—C47—H47C	109.5
			
C17—N1—C2—C3	116.05 (19)	C11—C12—C13—C14	0.2 (3)
C6—N1—C2—C3	−55.8 (2)	C12—C13—C14—C15	0.9 (3)
N1—C2—C3—N4	57.7 (2)	C13—C14—C15—C16	−1.1 (3)
C2—C3—N4—C41	167.03 (14)	C12—C11—C16—C15	0.9 (3)
C2—C3—N4—C5	−60.08 (19)	C17—C11—C16—C15	179.28 (17)
C41—N4—C5—C6	−169.72 (14)	C14—C15—C16—C11	0.2 (3)
C3—N4—C5—C6	58.43 (19)	C5—N4—C41—C46	−12.8 (2)
C17—N1—C6—C5	−117.53 (19)	C3—N4—C41—C46	117.23 (19)
C2—N1—C6—C5	54.7 (2)	C5—N4—C41—C42	164.13 (15)
N4—C5—C6—N1	−55.2 (2)	C3—N4—C41—C42	−65.8 (2)
C2—N1—C17—O17	−174.09 (19)	C46—C41—C42—O42	176.51 (16)
C6—N1—C17—O17	−2.9 (3)	N4—C41—C42—O42	−0.6 (2)
C2—N1—C17—C11	6.5 (3)	C46—C41—C42—C43	−2.1 (3)
C6—N1—C17—C11	177.72 (17)	N4—C41—C42—C43	−179.23 (16)
O17—C17—C11—C12	84.1 (2)	O42—C42—C43—C44	−176.56 (18)
N1—C17—C11—C12	−96.5 (2)	C41—C42—C43—C44	2.0 (3)
O17—C17—C11—C16	−94.2 (2)	C42—C43—C44—C45	−0.1 (3)
N1—C17—C11—C16	85.2 (2)	C43—C44—C45—C46	−1.5 (4)
C16—C11—C12—F12	179.99 (17)	C42—C41—C46—C45	0.5 (3)
C17—C11—C12—F12	1.7 (3)	N4—C41—C46—C45	177.51 (19)
C16—C11—C12—C13	−1.2 (3)	C44—C45—C46—C41	1.3 (4)
C17—C11—C12—C13	−179.50 (19)	C43—C42—O42—C47	5.4 (3)
F12—C12—C13—C14	179.1 (2)	C41—C42—O42—C47	−173.24 (17)

### 1-(2-Fluorobenzoyl)-4-(2-methoxyphenyl)piperazine (VI) Hydrogen-bond geometry (Å, º)

**Table:**

*D*—H···*A*	*D*—H	H···*A*	*D*···*A*	*D*—H···*A*
C15—H15···O17^i^	0.93	2.58	3.510 (3)	177
C6—H6*B*···*Cg*1^ii^	0.97	2.87	3.565 (2)	130

Symmetry codes: (i) *x*−1, *y*, *z*; (ii) −*x*+3/2, *y*+1/2, −*z*+1/2.
